# Cobalt-based metallo-mesoionic carbene gold complexes with antiproliferative effects

**DOI:** 10.1039/d5dt02549d

**Published:** 2025-12-17

**Authors:** D. Menia, F. R. Neururer, K. Wurst, P. Lippmann, I. V. Esarev, M. Seidl, I. Ott, M. Podewitz, S. Hohloch, B. Bildstein

**Affiliations:** a Department of General, Inorganic and Theoretical Chemistry, University of Innsbruck Innrain 80–82 6020 Innsbruck Austria Stephan.Hohloch@uibk.ac.at Benno.Bildstein@uibk.ac.at; b Institute of Medicinal and Pharmaceutical Chemistry, Technische Universität Braunschweig Beethovenstraße 55 38106 Braunschweig Germany ingo.ott@tu-braunschweig.de; c Institute of Materials Chemistry, Technische Universität Wien Getreidemarkt 9 1060 Wien Austria maren.podewitz@tuwien.ac.at

## Abstract

We report the facile synthesis of new gold(i) carbene complexes based on a mesoionic cobaltocenylidene metallocarbene *via* a fluorinative desilylation reaction. The carbene has been characterized by a variety of spectroscopic methods, revealing that it has the highest HEP value reported for a MIC so far, suggesting that the carbene is highly electron donating. The properties of the new class of metallo-mesoionic carbenes is further investigated, revealing also exceptionally low TEP values. Electrochemical studies also suggest the cobaltocenium moiety to be further reducible. In addition, the cell growth inhibitory effects of the new metallocarbene complexes were explored in cancer cells and bacteria. The combination of electrochemical activity, exceptional electron donating properties and their putative application in medicinal chemistry makes these new metallo-MICs a highly interesting new class of ligands.

## Abstract 2

We report the facile synthesis of new gold(i) carbene complexes based on a mesoionic cobaltocenylidene metallocarbene *via* a fluorinative desilylation reaction.

## Graphical abstract

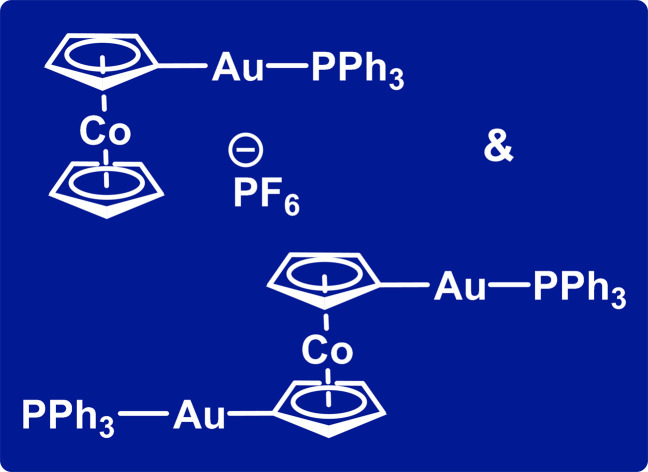

## Introduction

Undoubtedly, few ligand families have changed the chemical landscape as much as N-heterocyclic carbenes (NHCs).^1,2^ Since their first exploration by Wanzlick and Öfele^3^ and the subsequent isolation of the first free carbenes,^4^ NHCs have played a crucial role in the development and application of organometallic chemistry.^1,2,5^ It is therefore no surprise that over recent decades a plethora of subclasses have emerged, ranging from classical *n*NHCs to remote NHCs^6,7^ and cyclic alkyl/aryl-(amino) carbenes^8^ to abnormal or mesoionic carbenes.^6,7,9–12^ In particular, during the past two decades, the latter have emerged from laboratory curiosities to a valuable and important subclass of NHCs.^7,9,13^ This is partly related to the development of mesoionic carbenes (MICs) based on ubiquitously accessible triazoles (so-called triazolylidenes)^9–11,13^ as well as their unique electronic properties rating them as strongly electron donating carbenes. Given the modular approach of NHC synthesis, the donating abilities of the NHC ligands can be tuned quite easily. However, this approach requires the synthesis of new ligands, changing in most cases also the steric properties of the flanking groups. Thus, redox-active NHCs have emerged in the literature.^14–16^ These allow the tuning of the electronic structure of the NHC/MIC ligand by simple oxidation/reduction without the need for tedious synthetic remodeling. This has been achieved by either the introduction of redox-active groups into the backbone of the ligand (*e.g.* chinones or ferrocenes)^14,16^ or the introduction of redox-active wingtip groups, in most cases ferrocene or cobaltocene(ium) groups.^17^ However, in all these cases, the redox-active group is not an integral part of the NHC framework but covalently linked to it. In contrast to this, in 2018, we reported the synthesis of a purely metallocene-based mesoionic carbene termed 1-cobaltocenylidene 1a′ with a gold(iii) central metal.^18^ This carbene differs from previous redox-active carbenes because the carbene carbon is directly part of the redox-active group. However, the original synthesis lacked variability and only 1-cobaltocenylidene was accessible *via* this route while 1,1′-cobaltocenylidene was not.^18^ Furthermore, the reaction only proceeded from the 1-diazoniumcobaltocenium bis-hexafluorophosphate di-cation, which is tedious to prepare in a four-step synthesis and can only be stored for a prolonged time at low temperatures.^19^ Ultimately, the reaction starts from a gold(i) source, but a gold(iii) complex is finally isolated. Thus, we aimed to expand the synthesis to a more versatile and direct approach. Herein, we demonstrate the simple synthetic accessibility of 1-cobaltocenylidene and 1,1′-cobaltocenylidene making use of a fluorinative desilylation reaction starting from readily accessible 1,1′-trimethylsilylcobaltocenium hexafluorophosphate 1.^20^ A library of gold(i) complexes is reported and their respective redox, electronic and antiproliferative properties are explored.

## Results and discussion

Starting from readily accessible bis-TMS-cobaltocenium hexafluorophosphate [(Cp^TMS^)_2_Co][PF_6_] 1,^20^ we envisioned that in the presence of fluoride donors, C–Si bond cleavage would occur, which would allow trapping of the desired cobaltocenylidene species 2a or 2b using gold(i) precursors. Indeed, we found that after mixing CsF, 1 and (PPh_3_)AuCl (3 eq.) in acetonitrile, complexes 2a and 2b were obtained as air and moisture-stable yellow solids after chromatographic separation using an activated aluminum oxide column. Interestingly, the product distribution between 2a and 2b is highly dependent on the solvent. While in acetonitrile, 2a is the main product and 2b is a minor product, which can be isolated in 40% and 11% yields, respectively, in THF, 2b becomes the main product and can be isolated in 45% yield, while 2a is only accessible in yields of 20%. However, the reaction only proceeds well if 3 equivalents of (PPh_3_)AuCl are used, while lower amounts of the gold reagent lead to a high amount of hydrolysis with unsubstituted cobaltocenium hexafluorophosphate being the major product of the reaction. To further investigate the reaction mechanism for the formation of the desired cobaltocenylidene complexes, we performed the reaction in wet solvents, which almost exclusively led to the isolation of the protonated cobaltocenium salts. Furthermore, changing the fluoride source was also found to be detrimental to the formation of the cobaltocenylidene complexes. Thus, we propose that gold(i) in high concentration and a fluoride source of low solubility are needed to ensure that the concentration of the transient cobaltocenylidene species is as low as possible, and trapping it as rapidly as possible. However, a full mechanistic investigation of this reaction remains outstanding. Furthermore, the reaction also works with other gold(i) precursors such as (PMe_3_)AuCl; however, chromatographic separation of the corresponding trimethylphosphine complexes 3a and 3b was impossible. Formation of the new cobaltocenylidene complexes 2a and 2b is evident from several spectroscopic features. For the successful formation of 2a from 1, the first indication of formation is given by the observation of an “asymmetric” spin system in which one Cp ligand is completely protonated (*δ*^1^H = 5.52 ppm) while the other retains the typical AA′BB′ coupling pattern (Fig. S1).^18^ The expected *pseudo*-triplets appear at 5.76 and 5.42 ppm and are drastically shifted compared to those of 1 in which the Cp protons are overlayed to one multiplet at 5.72 ppm.^20^ This coupling pattern is retained in 2b for both Cp rings, but the signals are shifted to 5.61 and 5.32 ppm (Fig. S6). Furthermore, for both 2a and 2b, the typical phenyl protons are observed between 7.72 and 7.38 ppm. The presence of the triphenylphosphine moiety is also unambiguously indicated by the observation of a ^31^P NMR resonance at 42.9 and 43.0 ppm for 2a and 2b, respectively (Fig. S5/S10). Although the ^13^C carbene resonance was not directly observed *via* one-dimensional NMR spectroscopy (Fig. S2/S7), ^1^H–^13^C HMBC experiments showed a cross-peak at 5.76/120.0 and 5.42/120.0 ppm in 2a (Fig. S4) and at 5.61/118.0 and 5.32/118.0 ppm in 2b (Fig. S9), which unambiguously belong to the cobaltocenylidene carbon atom. Final proof for the successful 1- and 1,1′-metalation in 2a and 2b was given by X-ray diffraction analysis (Fig. 2, and S71, S72). The carbon–gold distances Au1–C1 and Au2–C10 are 2.038(3) Å in complex 2a and 2.070(2)/2.034(2) in complex 2b. Compared to classical gold(i) NHC complexes, such as (IPr)AuCl (C1–Au1 1.94 Å),^21^ (CAAC)AuCl (C1–Au1 1.98 Å),^22^ and even (MIC)AuCl (C1–Au1 1.97 Å),^23^ these distances are significantly longer, but comparable to that of cationic [(IPr)Au(PPh_3_)]^+^ (2.04 Å).^24^ The P1–Au1/P2–Au2 distances are comparable at 2.2941(8) Å in 2a and 2.2964(5)/2.2831(6) in 2b and similar to [(IPr)Au(PPh_3_)]^+^ (2.29 Å). The Au1–Au2 distance in 2b is 4.047(1) Å, which rules out any direct aurophilic Au–Au interactions in the complex.^25^ Although we were not able to completely separate the trimethylphosphine complexes 3a and 3b chromatographically from each other, unambiguous proof of their synthesis was given by X-ray diffraction analysis (Fig. S73, S74). Notably, while in 2b a torsion angle of 57.705(2)° was found between the two gold atoms, for 3b a torsion angle of 147.679(2)° was noted, resulting in an Au1–Au2 distance of 7.509(1) Å.

**Scheme 1:**
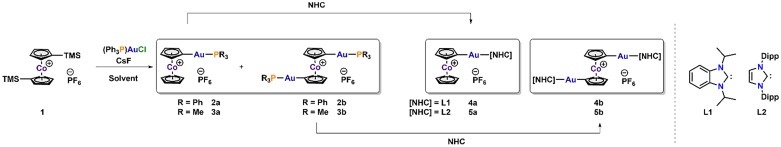
Synthetic procedure towards monometallic and bimetallic 1- and 1,1′-metallated cobaltocenylidene complexes of gold(i).

**Fig. 1:**
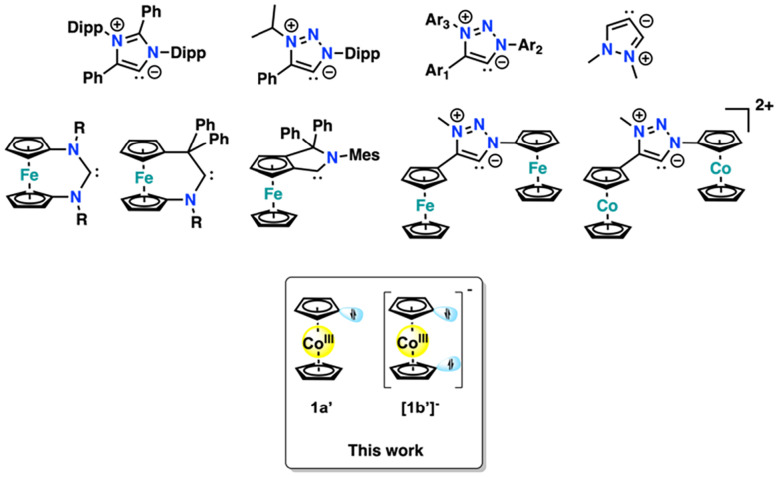
Selected overview of mesoionic carbenes (first row) and metallo-NHC/MIC ligands (middle row) known in the literature compared to the examples reported here. The first three examples of the first row correspond to ligands that are known to also exist in their free form.

**Fig. 2:**
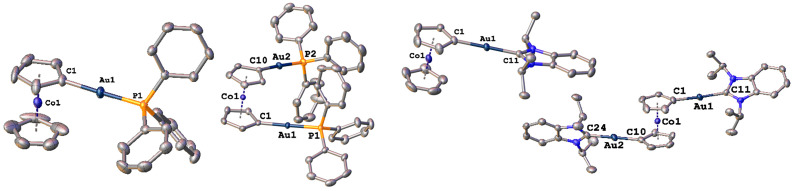
Molecular structures of complex 2a, 2b, 4a and 4b (from left to right). Hydrogen atoms, counter ions and lattice solvent molecules have been omitted for clarity. Ellipsoids are shown at a probability level of 50%.

**Fig. 3:**
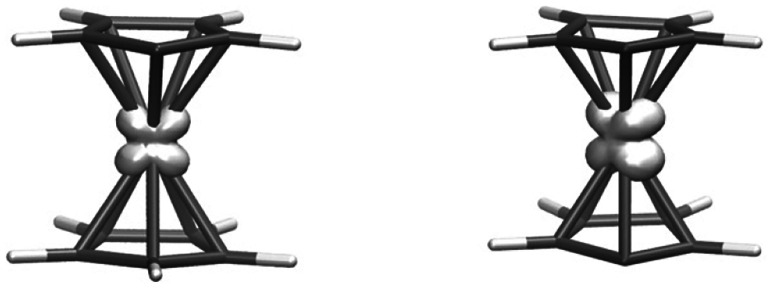
Spin density calculated with the TPSSh functional for [1a]^−^ and [1b]^2−^.

**Fig. 4:**
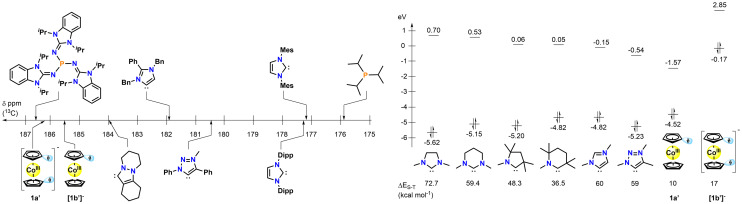
Cmparison of HEP values of different NHC, MIC and phosphine donors with 1a′ and 1b′ (left) and HOMO–LUMO gaps for different NHC/ligands including the (reduced) carbenes presented here (right).

The isolation of gold(i) complexes furthermore allowed the determination of the Huynh Electronic Parameter (HEP).^26^ Exchange of the triphenylphosphine ligands in 2a and 2b works well by simple addition of the free NHC ligands and gives access to the desired carbene complexes 4a and 4b in the case of NHC = BenziPr (L^1^Scheme 1) and 5a and 5b, if NHC = IPr (L^2^Scheme 1) is used. Successful ligand exchange is indicated by ^31^P NMR spectroscopy, which no longer shows any phosphine resonance. In contrast, the ^15^N NMR spectra show resonance at 244.8, 244.8, 244.8 and 244.9 ppm (Fig. S26, S35, S44, S50) indicative of the (benz)imidazolylidene ligands in 4a, 4b, 5a and 5b.^27^ Unambiguous proof for successful phosphine–NHC exchange is the appearance of a new singlet in the ^13^C NMR spectra of the new complexes at 193.7/194.4 ppm for 4a/4b (Fig. S28/S31) and at 193.0/193.9 ppm for 5a/5b (Fig. S40/S46), respectively. Using the formula recently reported by Huynh ([Pd] = 1.19[Au] − 45, with [Pd] and [Au] = chemical shift of the carbene resonance in ppm),^28^ the HEPs of 4a and 4b can be calculated to be 185.5 and 186.3 ppm, values that even exceed the highest HEP value of Pyry-C (Fig. 4),^29^ which was reported at 184.0 ppm, making 1a′ and [1b′]^−^ (see Fig. 1 or Fig. 4 for their structures) the most strongly donating MIC ligands reported so far.^30^ To place these values in more context, the HEP values of typical 2-imidazolylidenes, such as IPr, IMes, substituted benzimidazolylidenes and imidazolidines, appear in the range of 180.1–176.6 ppm, while HEPs of triazolylidenes (MIC) have been reported between 181.2 ppm and 179.5 ppm and even the more strongly donating 4-imidazolylidenes exhibit HEP values around 181.9 ppm. Thus, the new 1- and 1,1′-cobaltocenylidenes belong to the most strongly donating MICs reported so far and have (based on their HEP values) similar donor properties compared to Dielmann's electron-rich phosphines.^31^ The results from the HEP analysis are also in line with the calculated TEP parameters of the cobaltocenylidene moiety being 2037.1 cm^−1^ for the 1-cobaltocenylidene ligand 1a′^18^ and 1996.8 cm^−1^ for each carbene carbon of the 1,1′-cobaltocenylidene [1b′]^−^. The X-ray structures of the complexes 4a, 4b, 5a and 5b show the expected linear coordination geometries for gold(i) NHC/MIC complexes (Fig. 2 for 4a and 4b; Fig. S77 (5a) and S78 (5b)). The gold–cobaltocenylidene distances Au1–C1 and Au1–C10 are similar to those of the phosphine complexes 2,3a and 2,3b (*vide supra*) in the range between 2.016(6) Å and 2.025(3) Å, while those of the gold (benz)imidazolylidene ligands lie in between 2.022(2) Å and 2.041(6) Å and are comparable to those of [(IPr)Au(PPh_3_)]^+^.^24^ Given the redox-active nature of the cobaltocene(ylidene) moiety, we were further interested in their electrochemical properties. The phosphine complexes 2a and 2b show a reversible one-electron reduction at −1.55 and −1.82 V *vs.* Fc/[Fc]^+^ (Fig. S65 and S68), while the NHC congeners 4a,b and 5a,b exhibit reduction potentials of −1.68, −2.05, −1.75 and −2.05 V *vs.* Fc/[Fc]^+^. The lower reduction potentials of the NHC complexes 4 and 5 compared to 2 are in line with the higher donor strengths of the NHC ligands compared to phosphines. Unfortunately, we have not been able to chemically isolate any of these reduced complexes, but the values of the reduction potentials strongly suggest a cobalt-centred reduction process (Fig. 3).^32^ To further elucidate the electronic structure of the new carbenes 1a′ and [1b′]^−^ (Fig. 1 and 4), we turned to computational investigations. Computational investigations with density functional theory (PBE0/def2-TZVPP/D3BJ//BP86/def2-TZVPP/D3BJ in implicit polar solvent, see the SI for details) show that both complexes 2a and 2b adopt a singlet state with the corresponding triplet state energies being over 100 kJ mol^−1^ higher in energy (compare Table S3 and Fig. S79 and S80). Once reduced, the ground state of the species becomes a doublet, while the energy to the quartet state is 31.5 kJ mol^−1^ (7.5 kcal mol^−1^) and 6.7 kJ mol^−1^ (1.6 kcal mol^−1^, Table S3). To compare these data with other (mesoionic) carbenes (see Fig. 4), the HOMO–LUMO gaps in the free carbenes were calculated with B3LYP/def2-TZVPP and were found to be 2.95 eV and 3.02 eV for the isolated ligands 1a′ and [1b′]^−^, respectively. Calculating the singlet–triplet energy gaps in 1a′ and [1b′]^−^ with the same methodology yields 44.0 kJ mol^−1^ (10.5 kcal mol^−1^) and 72.2 kJ mol^−1^ (17.3 kcal mol^−1^) in favour of the singlet state. Since the reduced complexes are not chemically accessible, we computationally determined the TEP values of the reduced cobaltocenylidene ligands. In line with the reduction of the cobalt centre (and the enhancement of the electron density at the carbene moiety), the TEPs decrease from 2037.1 cm^−1^ in 1a′ to 2015.2 cm^−1^ in [1a′]^−^, and 1996.8 cm^−1^ (for each carbene carbon atom) in [1b′]^−^ to 1974.8 cm^−1^ in [1b′]^2−^. This proves that upon reduction, the cobaltocene(ii)–ylidene ligands become even stronger donors and would thus belong to the most strongly donating carbenes reported so far.

Finally, we performed preliminary investigations regarding the potential of these new MIC gold(i) complexes as prospective anticancer or antibacterial drug candidates. Due to their high stability and strong effects against cancer cells, gold NHC complexes have attracted increasing interest in inorganic medicinal chemistry.^33,34^ Given the fact that both gold(i) NHC^33,35^ and cobaltocene(ium) supported complexes^36^ have been shown to be quite potent against various tumorous and pathogenic cells,^33,37^ we envisioned the new complexes to be highly active. The growth inhibitory effects of the complexes were evaluated in several tumor cell lines, a non-tumor reference cell line, and two bacterial strains (Table 1). Complexes 2a, 2b, and 4a–5b generally triggered strong cytotoxic effects in MCF-7 (breast cancer), HT-29 (colon cancer) and A549 (lung cancer) cell lines with IC_50_ values in the low micromolar or sub-micromolar concentration range. Complexes 4a and 5a triggered stronger effects in these cancer cell lines than in the Vero-E6 non-tumor cell line (African green monkey kidney cells), which is a promising result regarding possible tumor selectivity. Interestingly, the cobaltocene derivatives with attached NHC complexes 5a and 5b triggered significantly stronger activity than a recently studied ferrocenyl-based NHC gold(i) complex in both cancer and non-tumor cells.^38^ As for their antibacterial activity, the complexes generally exhibited very low activity or complete inactivity against the Gram-negative *E. coli* bacterium; however, with the exception of 5b, all complexes demonstrated moderate to very strong activity against the Gram-positive *B. subtilis* strain. The exceptionally strong activity of 4b and 5a against *B. subtilis* and almost 100-fold selectivity of these two complexes in comparison with the results obtained with *E. coli* is noteworthy. Preference for Gram-positive bacteria over Gram-negative ones is a common effect of many gold compounds and can be attributed to the high sensitivity of Gram-positive bacteria towards gold-based thioredoxin reductase (TrxR) inhibitors.^33,39^ For a recently reported cobaltoceniumethynyl gold(i) complex, we had confirmed strong TrxR inhibition,^40^ indicating that inhibition of this essential enzyme is also a likely mechanism of action for the complexes of this report. Regarding structure–activity relationships for cytotoxic effects in cancer cells, there was a clear trend of the NHC ligands to increase bioactivity compared with the phosphine ligands. Importantly, the introduction of the cobaltocene core into the gold NHC unit tends to generally increase cytotoxic and antibacterial effects, as observed when compared to Auranofin. Thus, complex 5a was the most active complex against the investigated tumor cells. In summary, complex 5a emerged as the most promising compound of this study with strong and selective cell growth inhibitory effects against tumor cells as well as Gram-positive *B. subtilis*.

**Table 1:** Biological results: cytotoxic effects against cancer cells (MCF-7, HT-29, and A549) and one non-tumor cell line (Vero-E6) as well as antibacterial activity against one Gram-negative (*E. coli*) and one Gram-positive (*B. subtilis*) bacterial strain

	MCF-7	HT-29	A549	Vero-E6	*E. coli*	*B. subtilis*
Ciprofloxacin·HCl	n.d.	n.d.	n.d.	n.d.	0.021 (0.003)	0.15 (0.01)
Auranofin	2.2 (0.1)	3.0 (0.8)	3.6 (0.8)	2.2 (0.6)	n.d.	n.d.
2a	4.4 (0.9)	3.8 (1.0)	3.7 (1.4)	5.8 (0.5)	>50	8.6 (1.2)
2b	1.2 (0.2)	1.9 (0.6)	1.6 (0.2)	1.8 (0.1)	>50	18.1 (0.3)
4a	0.25 (0.10)	0.48 (0.15)	0.13 (0.08)	1.58 (0.41)	>50	5.3 (1.1)
4b	0.14 (0.13)	0.12 (0.04)	0.15 (0.01)	0.13 (0.02)	21.7 (5.7)	0.26 (0.02)
5a	0.3 (0.3)	0.3 (0.3)	0.3 (0.3)	1.0 (0.1)	27.5 (4.3)	0.30 (0.03)
5b	1.8 (0.2)	2.1 (0.1)	0.6 (0.2)	5.7 (0.2)	>50	>50
5b	1.8 (0.2)	2.1 (0.1)	0.6 (0.2)	5.7 (0.2)	>50	>50

## Conclusion

In conclusion, we have reported a versatile and simple access to new gold(i) complexes based on mesoionic 1-cobaltocenylidene and 1,1′-cobaltocenylidene carbene ligands. These carbenes were found to be among the most strongly donating MIC ligands reported so far and their donor properties can even be enhanced if the cobalt(iii) centre is further reduced to cobalt(ii). The biological evaluation highlighted complexes 4b and 5a as highly efficacious regarding their cytotoxic and Gram-positive selective antibacterial activity. Future work will focus on the implementation of these new, redox-active carbenes in switchable processes, especially in catalysis, and on further evaluating the mechanism and assessing their biological activity.

## Conflicts of interest

There are no conflicts to declare.

## Supplementary Material

DT-055-D5DT02549D-s001

DT-055-D5DT02549D-s002

## Data Availability

All data are available free of charge from our side if requested. NMRs, IR and cyclic voltammograms have been included (plotted) in the supplementary information (SI). Raw data is stored on the university servers and can be accessed *via* us if necessary. Crystallographic data (CIF-files) have been uploaded to the CCDC. Raw data and frames are stored on the university servers and can be accessed *via* us if necessary. The authors have cited additional references within the SI.^41^ Supplementary information: ^1^H, ^13^C, ^31^P and 2D NMR spectra, IR and UV-Vis data as well as further information regarding X-ray crystallography and computational investigations and antiproliferative testing. See DOI: https://doi.org/10.1039/d5dt02549d. CCDC 2314980 (2a), 2314979 (2b), 2476574 (3b), 2314982 (4a), 2314981 (4b), 2314983 (5a) and 2314984 (5b) contain the supplementary crystallographic data for this paper.^42*a*–*g*^
