# Supplementary material for: Cobalt-based metallo-mesoionic carbene gold complexes with antiproliferative effects
Source: Dalton Trans. 2025 Dec 17;55(8):3259–65. doi: 10.1039/d5dt02549d (PMC12871041; doi:10.1039/d5dt02549d)
Supplement: DT-055-D5DT02549D-s001 [file DT-055-D5DT02549D-s001.pdf]

Supplementary Information (SI) for Dalton Transactions.
This journal is © The Royal Society of Chemistry 2025

Cobalt-Based Metallo-Mesoionic Carbene Gold Complexes with Antiproliferative Effects

Daniel Menia,[a] Florian Neururer,[a] Klaus Wurst,[a] Petra Lippmann,[b] Igor V. Esarev,[b] Michael Seidl,[a] Ingo Ott,* Maren Podewitz,* Stephan Hohloch* and Benno Bildstein*

Table of Contents

Experimental Procedures 2
NMR Spectra 5
IR Spectra 55
ESI – MS 58
Cyclic voltammetry 62
Crystallographic data 66
Computational Details 76
References 80

Experimental Procedures

General Remarks:

Unless stated otherwise, all transformations were conducted under an inert atmosphere using standard Schlenk techniques or an argon filled glove box (GS Mega line). High temperature reactions were carried out in preheated oil baths or aluminium blocks. Low temperature reactions were performed using a precooled aluminium block or dewar vessels filled with cooling mixtures consisting of ethanol/liquid nitrogen. Solvents for synthetic purposes were purified using an *MBraun* SPS system and stored over activated molecular sieves. Additionally, THF, 1,4-Dioxane, and 1,2-Dimethoxyethane (DME) were dried and distilled over sodium/benzophenone. Solvents used for aqueous workup or chromatography (technical grade) were used as received by a commercial supplier. All commercially available reagents were used as received. Deuterated solvents (benzene-d_6 and dichloromethane-d_2) were stored over activated molecular sieves. NMR spectra were recorded on a *400 MHz Bruker Avance 4 Neo* spectrometer. ^{1}H and ^{13}C{^{1}H} chemical shifts are reported in ppm and calibrated using residual solvent resonances. ATR-Infrared spectroscopy was conducted on a *Bruker Alpha* IR spectrometer. UV-Vis-NIR spectra were recorded using an Avantes Ava-Spec NIR 1.7 spectrometer. 1,1'–bis(trimethylsilyl) cobaltocenium hexafluorophosphate was prepared following a previously reported protocol.[1]

X-ray crystallography

X-ray diffraction crystallography was performed at the University of Innsbruck (Bruker D8 Quest) All crystals were kept at low temperatures throughout data collection. Data collection, refinement and reduction was performed using the ApexIV. All structures were solved with SHELXT[2] and refined using the OLEX 2 software package[3] using SHELXL least square refinements.[4] Strongly disordered solvent molecules have been removed using the SQUEEZE operation.[5] All nonhydrogen atoms were refined anisotropically, and hydrogen atoms were included at the geometrically calculated positions and refined using a riding model. Non-substituted Cp rings in structures **2a** and **5a** were rotationally disordered and modelled using a 55:45 occupancy (**2a**) and 68:32 (**5a**) using RIGU and SIMU restraints. Crystals of **3a** were found to be a two-component twin which could only be moderately resolved. Additionally, the crystals were only weakly diffracting, for which no bond lengths etc. are discussed in Table S2. Nevertheless, the model itself is valid and atom connectivity can be unambiguously determined.

Cell culture

A549 lung carcinoma cells, MCF-7 breast cancer cells, HT29 colorectal adenocarcinoma cells (both DSMZ-German Collection of Microorganisms and Cell Cultures GmbH, Germany) and Vero E6 healthy kidney epithelial cells (Cytion GmbH (formerly CLS cell line services), Germany) were maintained in Dulbecco's modified Eagle's medium (DMEM; 4.5 g/L D-glucose, L-glutamine, pyruvate), supplemented with fetal bovine serum superior, standardized (Biochrom GmbH, Berlin, 10 % v/v) and gentamycin (50 mg/L) with a weekly passage. Cells were grown in tissue culture flasks (75 cm^2, TPP Life Sciences Co., Ltd., Korea). Cell lines were grown at 37 °C in a humidified atmosphere of 95% air and 5% CO2 and passaged when they reached 80–90% confluency.

Antiproliferative assay in tumorigenic and non-tumorigenic cells

The antiproliferative effects were determined according to a previously published procedure.[6] A volume of 100 µL of A549 cells (10000 cells/mL), MCF-7 cells (8000 cells/mL), HT 29 cells (25000 cells/ml) and Vero E6 cells (3000 cells/mL) was transferred into the wells of a 96-well plate and incubated at 37 °C under 5 % CO_2 for 48 h (A549, HT-29) or 72 h (MCF-7, Vero-E6). Stock solutions of the compounds were freshly prepared in dimethyl sulfoxide (DMSO) and diluted with the appropriate cell medium to obtain various concentrations in 0.1 % v/v DMSO solutions. After 72 h (A549, HT29) or 96 h (MCF-7, Vero E6) of exposure, the biomass of the cells was determined via crystal violet staining, and the (IC_{50}) value was determined as the concentration that caused 50 % inhibition of cell proliferation relative to an untreated control. The results were calculated as the mean values of three independent experiments, unless stated otherwise.

Culture of bacteria

Gram-negative strain *Escherichia coli* (DSM1116, ATCC 9637) and Gram-positive strain *Bacillus subtilits* (DSM 109511) were obtained from DSMZ (German Collection of Microorganisms and Cell Cultures GmbH, Braunschweig, Germany) as a lyophilized cell pellet. The pellet was resuspended in respective culture medium containing 10% glycerol: Müller Hinton broth (MHB, 21 g/L, pH 7.4) for E.coli and in Tryptic soy yeast broth (TSY, 30 g/L trypticase soy broth, 3 g/L yeast extract, pH 7.0–7.2) for B.subtilis. The suspensions were aliquoted into cryovials, and they were kept frozen over liquid nitrogen. This frozen stock was then used to deliver bacterial cells on a Müller-Hinton agar plate (MHA, 38 g/L, pH 7.0) for *E.col*i or on a Tryptic soy yeast agar (TSYA, 40 g/L trypticase soy agar, 3 g/L yeast extract, pH 7.3) for *B.subtilis*. The plates were incubated at 37°C for 24 h to obtain bacterial colonies.

Antibacterial Activity

The bacterial suspension of *E.coli* DSM 1116 was prepared by overnight incubation of a single colony at 37°C in MHB. Minimum inhibitory concentration (MIC) values were determined following a standardized protocol in broth microdilution assays.[7] In short: The compounds were dissolved as stock solutions in DMSO and diluted with 100 mkl of MHB to concentrations in the range of 128 to 2.0 mkM in 96-well microtiter plates. 100 mL of starting bacterial inocula of 2–8 x 10^5 CFU mL^{-1} in MHB at 37 °C was added to all wells except negative growth control (final DMSO concentraion: 1%) The serial dilutions of test compounds were prepared in duplicate. After incubation of the plates for 22 h at 37°C, the absorbance at 600 nm was measured using a Perkin-Elmer 2030 Multilabel Reader VICTORTM X4 microplate reader. MIC (the lowest concentration of compound that completely inhibits the cell growth) was determined in three independent experiments by dose-response curve fitting with Origin Pro (OriginLab Corp.). The same procedure was performed to test suspensions of *B.subtilis*, except that TSY was used for culturing and dilution.

Synthetic procedures:

Mono(triphenylphosphine gold(I)) cobaltocenylidene hexafluorophosphate (2a) and 1,1'–bis(triphenylphosphine gold(I)) cobaltocenylidene hexafluorophosphate (2b) –

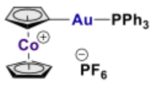

2a

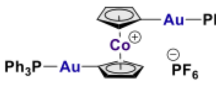

2b

Method A: In a Schlenk flask 1,1'–bis(trimethylsilyl) cobaltocenium hexafluorophosphate[1] (300 mg, 0.627 mmol, 1eq.) and triphenylphosphine gold(I) chloride (930 mg, 1.881 mmol, 3 eq.) were combined with 50 ml acetonitrile and the suspension stirred for 12 h. Then caesium fluoride (380 mg, 2.508 mmol, 4 eq.) was added. After 14 days, the precipitant was filtered off through a paper filter. The solvent was removed in vacuo and the product was washed with toluene (3 x 20 ml) and diethyl ether (3 x 20 ml). The residue was solved in 50 ml acetonitrile and approximately 3 g AlOx were added, and the solvent was removed on a rotatory evaporator with a minimum of vacuum. A plug of AlOx (Diameter = 4.5 cm, h = 2.5 cm) was equilibrated with Et_2O/DCM 2.5:1 and the residue on AlOx, consisting of cobaltocenium, **2a** and **2b**, was poured on top. After washing with 1400 ml Et_2O/DCM 2.5:1, **2a** was eluted with 2000 ml Et2O/DCM 2:1. All volatiles were evaporated and pure **2a** could be obtained as a yellow powder (92.4 mg, 0.074 mmol) with 11% yield. Single crystals were obtained by diffusion-crystallization from acetonitrile/diethyl ether. Due to partial hydrolysis the main product on this route is **2a**, which was eluted with 1500 ml Et2O/DCM 1:2 and after evaporation of the solvent **2a** could be isolated as a yellow powder (200.2 mg, 0.252 mmol) with 40% yield.

Method B: In a Schlenk flask 1,1'–bis(trimethylsilyl) cobaltocenium hexafluorophosphate[1] (50 mg, 0.104 mmol, 1eq.) and triphenylphosphine gold(I) chloride (155 mg, 0.313 mmol, 3 eq.) were combined with 15 ml THF and stirred for 1 h. Then cesium fluoride (65 mg, 0.418 mmol, 4 eq.) was added. After 48 h, the precipitant was filtered off through a paper filter. The solvent was removed in vacuo and the product was washed with toluene (3 x 20 ml) and diethyl ether (3 x 20 ml). The residue was solved in 10 ml acetonitrile and approximately 3 g AlOx were added, and the solvent was removed on a rotatory evaporator with a minimum of vacuum. A plug of AlOx (Diameter = 4.5 cm, h = 2.5 cm) was equilibrated with Et_2O/DCM 2.5:1 and the residue on AlOx, consisting of cobaltocenium, **2a** and **2b**, was poured on top. After washing with 1400 ml Et_2O/DCM 2.5:1, **2b** was eluted with 2000 ml Et_2O/DCM 2:1. All volatiles were evaporated and pure **2b** could be obtained as a yellow powder (58 mg, 0.047 mmol) with 45% yield. **2a** was eluted with 1500 ml Et2O/DCM 1:2 and after evaporation of the solvent **2a** could be isolated as a yellow powder (16 mg, 0.026 mmol) with 20% yield.

Analytical data for **2a**: ^{1}H NMR (400 MHz, CD_3CN): δ (ppm) = 7.61 (m, 15H, Aryl-*H*), 5.76 (t, J_{HH} = 1.7 Hz, 2H, Cp^{Au}), 5.52 (s, 5H, Cp*H*), 5.42 (t, J_{HH} = 1.7 Hz, 2H, $Cp^{Au}H$). ^{13}C NMR (101 MHz, CD_3CN): δ (ppm) = 135.1 (d, $^2J_{CP}$ = 13.8 Hz, Aryl-*C*H), 132.9 (d, $^4J_{CP}$ = 2.5 Hz, Aryl-*C*H), 130.7 (d, $^1J_{CP}$ = 53.6 Hz. Aryl-*C*), 130.5 (d, $^3J_{CP}$ = 11.0 Hz, Aryl-*C*H), 93.3 ($Cp^{Au}C$H), 87.0 ($Cp^{Au}C$H), 84.7 (Cp*C*H). ^{31}P NMR (162 MHz, CD_3CN) δ (ppm) = 42.9 (s, 1P, *P*Ph_3), −144.6 (hept, J_{PF} = 706 Hz, 1P, $[PF_6]^-$) ppm. MS (ESI+): m/z calc. 647.0608 (M+), found 647.0604 (M+). Melting point: 233.5 °C.

Analytical data for **2b**: ^{1}H NMR (400 MHz, CD_3CN): δ (ppm) = 7.50 (m, 30H, Aryl-*H*), 5.61 (t, J_{HH} = 1.7 Hz, 4H, Cp^{Au}-*H*), 5.32 (t, J_{HH} = 1.7 Hz, 4H, Cp^{Au}-) ppm. ^{13}C NMR (101 MHz, CD_3CN): δ (ppm) = 135.1 (d, $^2J_{CP}$ = 13.8 Hz, Aryl-*C*H), 132.7 (d, $^4J_{CP}$ = 2.5 Hz, Aryl-*C*H), 130.8 (d, $^1J_{CP}$ = 53.6 Hz. Aryl-*C*), 130.4 (d, $^3J_{CP}$ = 11.0 Hz, Aryl-*C*H), 92.8 ($Cp^{Au}C$H), 86.0 ($Cp^{Au}C$H). ^{31}P NMR (162 MHz, CD_3CN) δ (ppm) = 43.0 (s, 2P, *P*Ph_3), −144.6 (sept., $^1J_{PF}$ = 706 Hz, 1P, $[PF_6]^-$) ppm. MS (ESI+): m/z calc. 1105.1106 (M+), found 1105.1081 (M+). Melting point: 240.5 °C.

Mono(trimethylphosphine gold(I)) cobaltocenylidene hexafluorophosphate (3a) and 1,1'–bis(trimethylphosphine gold(I)) cobaltocenylidene hexafluorophosphate (3b)

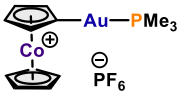

3a

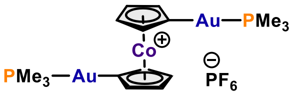

3b

In a Schlenk flask 1,1'–bis(trimethylsilyl) cobaltocenium hexafluorophosphate (300 mg, 0.627 mmol, 1eq.) and trimethylphosphine gold(I) chloride (580 mg, 1.881 mmol, 3 eq.) were combined with 50 ml acetonitrile and the suspension stirred for 1 h. Then cesium fluoride (380 mg, 2.508 mmol, 4 eq.) was added. After 5 days, the precipitant was filtered off through a paper filter. The solvent was removed in vacuo and the product was washed with toluene (3 x 20 ml) and diethyl ether (3 x 20 ml). The residue was solved in 50 ml acetonitrile and approximately 3 g AlOx were added, and the solvent was removed on a rotatory evaporator with a minimum of vacuum. A plug of AlOx (Diameter = 4.5 cm, h = 2.5 cm) was equilibrated with Et_2O/DCM 4:1 and the residue on AlOx, consisting of cobaltocenium, **3a** and **3b**, was poured on top. **3b** was eluted with a gradient consisting of 700 ml Et_2O/DCM 2.75:1, 730 ml Et_2O/DCM 2.65:1, 720 ml Et_2O/DCM 2.6:1, 3550 ml Et_2O/DCM 2.55:1. **3a** was then eluted with 750 ml Et_2O/DCM 1:2. Both fractions contain a significant amount of the bis(trimethylsilyl) gold(I) cation. Also, the fraction of **3a** contains a large amount of **3b**. Single crystals were obtained by diffusion-crystallization from acetonitrile/diethyl ether.

Analytical data for **3a**: ^{1}H NMR (400 MHz, CD_3CN): δ (ppm) = 5.71 (q, J_{HH} = 1.6 Hz, 2H, $Cp^{Au}H$), 5.51 (s, 5H, Cp*H*), 5.27 (q, J_{HH} = 1.6 Hz, 2H, $Cp^{Au}H$), 1.56 (d, 9H, P(CH_3)$_3$) ppm. ^{13}C NMR (101 MHz, CD_3CN): δ (ppm) = 93.2 (d, $^4J_{CP}$ = 4.3 Hz, $Cp^{Au}C$H), 86.9 (d, $^3J_{CP}$ = 5.3 Hz, $Cp^{Au}C$H), 84.4 (Cp*C*), 15.0 (d, $^1J_{CP}$ = 35.4 Hz, P(*C*H_3)$_3$). ^{31}P NMR (162 MHz, CD_3CN) δ (ppm) = 5.9 (dq, J_{CP} = 21.1, 10.6 Hz), *P*Me_3), −144.62 (sept, $^1J_{PF}$ = 706 Hz, 1P, $[PF_6]^-$) ppm. MS (ESI+): m/z calc. 461.0138 (M^+), found 461.0125 (M^+).

Analytical data for **3b**: ^{1}H NMR (400 MHz, CD_3CN): δ (ppm) = 5.56 (q, J_{HH} = 1.6 Hz, 4H, $Cp^{Au}H$), 5.17 (q, J_{HH} = 1.6 Hz, 4H, $Cp^{Au}H$), 1.54 (t, J_{PH} = 5.1 Hz). ^{13}C NMR (101 MHz, CD_3CN): δ (ppm) = 92.3 (d, $^4J_{CP}$ = 4.5 Hz, $Cp^{Au}C$H), 85.8 (d, $^3J_{CP}$ = 5.6 Hz, $Cp^{Au}C$H), 14.t (t, J_{CP} = 19.4 Hz, P(CH_3)$_3$). ^{31}P NMR (162 MHz, CD_3CN) δ (ppm) = 8.5 (m, 2P, *P*Me_3), −144.6 (sept, $^1J_{PF}$ = 706. Hz, 1P, $[PF_6]^-$) MS (ESI+): m/z calc. 733.0167 (M^+), found 733.0143 (M^+).

Mono(1,3-diisopropylbenzimidazolin-2-ylidene gold(I)) cobaltocenylidene hexafluorophosphate (4a)

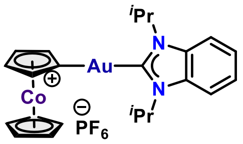

In a Schlenk flask **2a** (50 mg, 0.063 mmol, 1eq.) and 1,3-diisopropylbenzimidazolin-2-ylidene (16 mg, 0.075 mmol, 1.2 eq.) were solved in 20 ml THF and stirred for 24 h, resulting in a green solution. The solvent was removed in vacuo and the product was washed with toluene (3 x 30 ml) and diethyl ether (3 x 20 ml). For better solubility, an ultrasonic bath was used. After drying **4a** could be isolated as a yellow powder (35 mg, 0.048 mmol) with 76% yield. Single crystals were obtained by diffusion-crystallization from acetonitrile/diethyl ether. ^{1}H NMR (400 MHz, CD_3CN): δ (ppm) = 7.80 (m, 2H, Aryl-C*H*), 7.46 (m, 2H, Aryl-C*H*), 5.74 (m, 2H, Cp^{Au}*H*), 5.51 (s, 5H, Cp*H*), 5.47 (sept, J_{HH} = 7 Hz, 2H, C*H*(CH_3)$_2$), 5.42 (m, 2H, Cp^{Au}*H*), 1.82 (d, J_{HH} = 7 Hz, 12H, CH(C*H*$_3$)$_2$). ^{1}H NMR (400 MHz, $CDCl_3$): δ (ppm) = 7.66 (m, 2H, Aryl-C*H*), 7.39 (m, 2H, Aryl-C*H*), 5.78 (m, 2H, Cp^{Au}*H*), 5.53 (s, 5H, Cp*H*), 5.45 (m, 2H, Cp^{Au}*H*), 5.44 (hept, J_{HH} = 7 Hz, 2H, C*H*(CH_3)$_2$), 1.83 (d, J_{HH} = 7 Hz, 12H, CH(C*H*$_3$)$_2$). ^{13}C NMR (101 MHz, CD_3CN): δ (ppm) = 194.5 (benzimidazole-2-ylidene-*C*), 133.7 (Aryl-*C*), 124.9 (Aryl-*C*H), 113.8 (Aryl-*C*H), 93.7 (Cp^{Au}CH), 86.7 (Cp^{Au}CH), 84.5 (Cp*C*H), 53.7 (*C*H(CH_3)$_2$), 22.7 (CH(*C*H$_3$)$_2$). ^{13}C NMR (101 MHz, $CDCl_3$): δ (ppm) = 193.7 (benzimidazole-2-ylidene-*C*), 132.8 (Aryl-*C*), 124.1 (Aryl-*C*H), 113.0 (Aryl-*C*H) 93.1 (Cp^{Au}CH), 85.9 (Cp^{Au}CH), 83.6 (Cp*C*H), 53.5 (*C*H(CH_3)$_2$), 22.4 (CH(*C*H$_3$)$_2$) ^{31}P NMR (162 MHz, CD_3CN) δ (ppm) = −144.6 (hept, $^{1}J_{PF}$ = 706 Hz, 1P, [PF_6]$^-$). ^{15}N NMR (41 MHz, CD_3CN) δ (ppm) = 244.8 (benzimidazolylidene-*N*). MS (ESI+): m/z calc. 587.1166 (M^+), found 587.1134 (M^+). Melting point: 177.1 °C.

1,1'–bis(1,3-diisopropylbenzimidazolin-2-ylidene gold(I)) cobaltocenylidene hexafluorophosphate (4b)

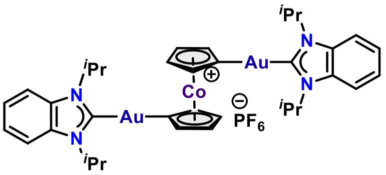

In a Schlenk flask **2b** (50 mg, 0.040 mmol, 1eq.) and 1,3-diisopropylbenzimidazolin-2-ylidene (18 mg, 0.088 mmol, 2.2 eq.) were solved in 20 ml THF and stirred for 24 h, resulting in a green solution. The solvent was removed in vacuo and the product was washed with toluene (3 x 30 ml) and diethyl ether (3 x 20 ml). For better solubility, an ultrasonic bath was used. After drying **4b** could be isolated as a yellow powder (33.1 mg, 0.029 mmol) with 73% yield. Single crystals were obtained by diffusion-crystallization from acetonitrile/diethyl ether. ^{1}H NMR (400 MHz, CD_3CN): δ (ppm) = 7.73 (m, 4H, Aryl-C*H*), 7.39 (m, 4H, Aryl-C*H*), 5.58 (t, J_{HH} = 1.6 Hz, 4H, Cp^{Au}*H*), 5.44 (hept, J_{HH} = 6.8 Hz, 4H, C*H*(CH_3)$_2$), 5.27 (t, 4H, J_{HH} = 1.6 Hz, 4H, Cp^{Au}*H*), 1.69 (d, J = 7.0 Hz, 24H, CH(C*H*$_3$)$_2$). ^{1}H NMR (400 MHz, $CDCl_3$): δ (ppm) = 7.64 (m, 4H, Aryl-C*H*), 7.38 (m, 4H, Aryl-C*H*), 5.59 (t, J_{HH} = 1.6 Hz, 4H, Cp^{Au}*H*), 5.48 (hept, J_{HH} = 6.8 Hz, 4H, C*H*(CH_3)$_2$), 5.27 (t, J_{HH} = 1.6 Hz, 4H, Cp^{Au}*H*), 1.80 (d, J = 7 Hz, 24H, CH(C*H*$_3$)$_2$).zz ^{13}C NMR (101 MHz, CD_3CN): δ (ppm) = 195.2 (benzimidazole-2-ylidene-*C*), 133.6 (Aryl-*C*), 124.8 (Aryl-*C*H), 115.3 (Cobaltoceneylidene-*C*), 113.8 (Aryl-*C*H), 93.0 (Cp^{Au}*C*), 85.4 (Cp^{Au}*C*), 53.8 (*C*H(CH_3)$_2$), 22.5 (CH(*C*H$_3$)$_2$). ^{13}C NMR (101 MHz, $CDCl_3$): δ (ppm) = 194.4 (Benzimidazole-2-ylidene-*C*), 132.8 (Aryl-*C*), 124.1 (Aryl-*C*H), 144.3 (Cobaltoceneylidene-*C*), 113.0 (Aryl-*C*H), 92.0 (Cp^{Au}*C*H) 94.7 (Cp^{Au}*C*H), 53.3 (*C*H(CH_3)$_2$), 22.4 (CH(*C*H$_3$)$_2$). ^{31}P NMR (162 MHz, CD_3CN) δ (ppm) = −144.6 (hept., $^{1}J_{PF}$ = 706 Hz, 1P, [PF_6]$^-$). ^{15}N NMR (41 MHz, CD_3CN) δ (ppm) = 244.8 (s, 1N, benzimidazolylidene-*N*). MS (ESI+): m/z calc. 985.2224 (M^+), found 985.2173 (M^+). Melting point: 220.1 °C (dec.).

Mono(1,3-bis-(2,6-diisopropylphenyl)-1,3-dihydro-2H-imidazol-2-ylidene gold(I)) cobaltocenylidene hexafluorophosphate (5a)

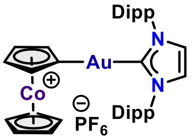

In a Schlenk flask **2a** (105 mg, 0.133 mmol, 1eq.) and 1,3-bis-(2,6-diisopropylphenyl)-1,3-dihydro-2H-imidazol-2-ylidene (62 mg, 0.159 mmol, 1.2 eq.) were solved in 50 ml THF and stirred for 24 h, resulting in a green solution. The solvent was removed in vacuo and the product was washed with toluene (3 x 30 ml) and diethyl ether (3 x 20 ml). For better solubility, an ultrasonic bath was used. After drying **5a** could be isolated as yellow crystals (119 mg, 0.129 mmol) with 98% yield. Single crystals were obtained by diffusion-crystallization from acetonitrile/diethyl ether. ^{1}H NMR (400 MHz, CD_3CN): δ (ppm) = 7.52 (m, 4H, Aryl-H), 7.41 (d, J_{HH} = 7.5 Hz, 4H, Aryl-H), 5.48 (pseudo-t, J_{HH} = 1.8 Hz, 2H, Cp^{Au}*H*), 4.93 (s, 5H, Cp*H*), 4.90 (pseudo-t, J_{HH} = 1.8 Hz, 2H, Cp^{Au}*H*), 2.64 (hept, J_{HH} = 6.9 Hz, 4H, C*H*(CH_3)$_2$), 1.40 (d, J_{HH} = 6.9 Hz, 12H, CH(C*H*$_3$)$_2$), 1.26 (d, J_{HH} = 6.9 Hz, 12H, CH(C*H*$_3$)$_2$). ^{13}C NMR (101 MHz, CD_3CN): δ (ppm) = 193.0 (imidazole-2-ylidene-*C*), 147.1 Aryl-*C*H), 135.4 (Aryl-C), 131.6 (Aryl-*C*H), 125.1 (Aryl-*C*H), 124.9 (Aryl-*C*H), 116.3 (Cobaltoceneylidene-*C*), 93.2 (Cp^{Au}CH), 86.5 (Cp^{Au}CH), 83.9 (Cp*C*H), 29.6 (*C*H(CH_3)$_2$), 24.6 (CH(*C*H$_3$)$_2$), 23.9 (zzCH(*C*H$_3$)$_2$). ^{31}P NMR (162 MHz, CD_3CN) δ (ppm) = −144.6 (hept., $^{1}J_{PF}$ = 706 Hz, 1P, [PF_6]$^-$). ^{15}N NMR (41 MHz, CD_3CN) δ (ppm) = 244.8 ppm (imidazolylidene-*N*). MS (ESI+): m/z calc. 773.2575 (M^+), found 773.2550 (M^+). Melting point: >250 °C.

1,1'–bis(1,3-bis-(2,6-diisopropylphenyl)-1,3-dihydro-2H-imidazol-2-ylidene gold(I)) cobaltocenylidene hexafluorophosphate (5b)

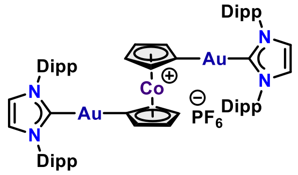

In a Schlenk flask **2b** (89 mg, 0.071 mmol, 1eq.) and 1,3-bis-(2,6-diisopropylphenyl)-1,3-dihydro-2H-imidazol-2-ylidene (61 mg, 0.157 mmol, 2.2 eq.) were solved in 50 ml THF and stirred for 24 h, resulting in a green solution. The solvent was removed in vacuo and the product was washed with toluene (3 x 30 ml) and diethyl ether (3 x 20 ml). For better solubility, an ultrasonic bath was used. After drying **5b** could be isolated as a yellow crystals (55 mg, 0.071 mmol) in 51% yield. Single crystals were obtained by diffusion-crystallization from acetonitrile/diethyl ether. ^{1}H NMR (400 MHz, CD_3CN): δ (ppm) = 7.47 (m, 8H, Aryl-*H*), 7.36 (d, J = 7.5 Hz, 8H, Aryl-*H*), 4.56 (t, J_{HH} = 1.6 Hz, 4H, Cp^{Au}*H*), 4.29 (t, J_{HH} = 1.6 Hz, 4H, Cp^{Au}*H*), 2.60 (hept, J_{HH} = 6.9 Hz, 8H, C*H*(CH_3)$_2$), 1.34 (d, J_{HH} = 6.9 Hz, 24H, CH(C*H*$_3$)$_2$), 1.24 (d, J_{HH} = 6.9 Hz, 24H, CH(C*H*$_3$)$_2$).^{13}C NMR (101 MHz, CD_3CN): δ (ppm) = 193.8 (benzimidazole-2-ylidene-*C*), 147.0 (Aryl-*C*H), 135.4 (Aryl-*C*), 131.50 (Aryl-*C*H), 125.0 (Aryl-*C*H), 124.8 (Aryl-*C*H), 113.9 (cobaltoceneylidene-*C*), 91.6 (Cp^{Au}CH), 84.9 (Cp^{Au}CH), 29.6 (*C*H(CH_3)$_2$), 24.9 (CH(*C*H$_3$)$_2$), 24.0 (CH(*C*H$_3$)$_2$), ^{31}P NMR (162 MHz, CD_3CN) δ (ppm) = −144.6 (hept., $^{1}J_{PF}$ = 706 Hz, 1P, [PF_6]$^-$). ^{15}N NMR (41 MHz, CD_3CN) δ (ppm) = 244.9 (imidazolylidene-*N*). MS (ESI+): m/z calc. 1357.5041 (M^+), found 1357.4998 (M^+). Melting point: >250 °C.

NMR Spectra

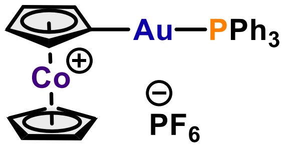

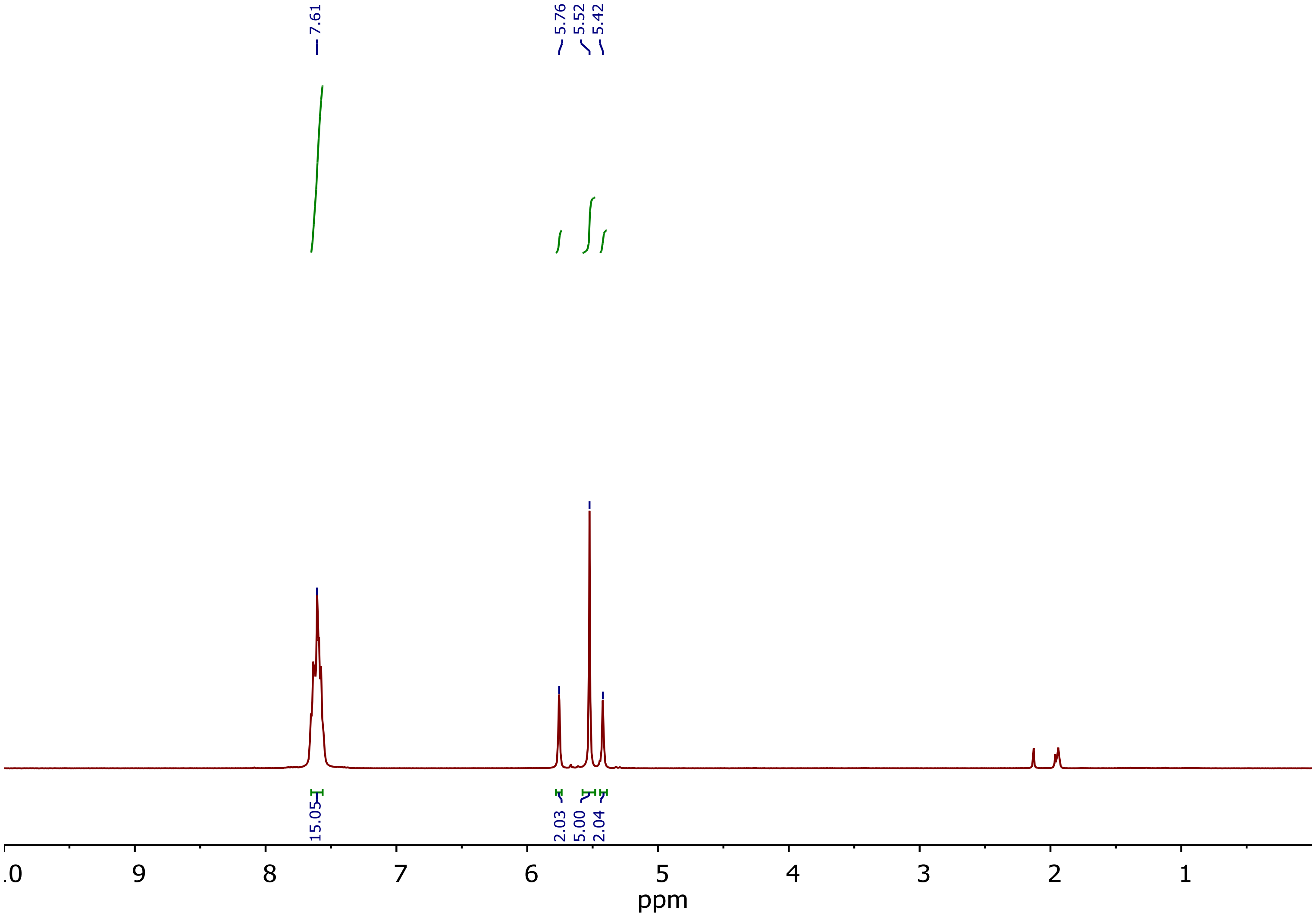

*Figure S 1: ^{1}H NMR (400 MHz, CD_3CN) **2a**.*

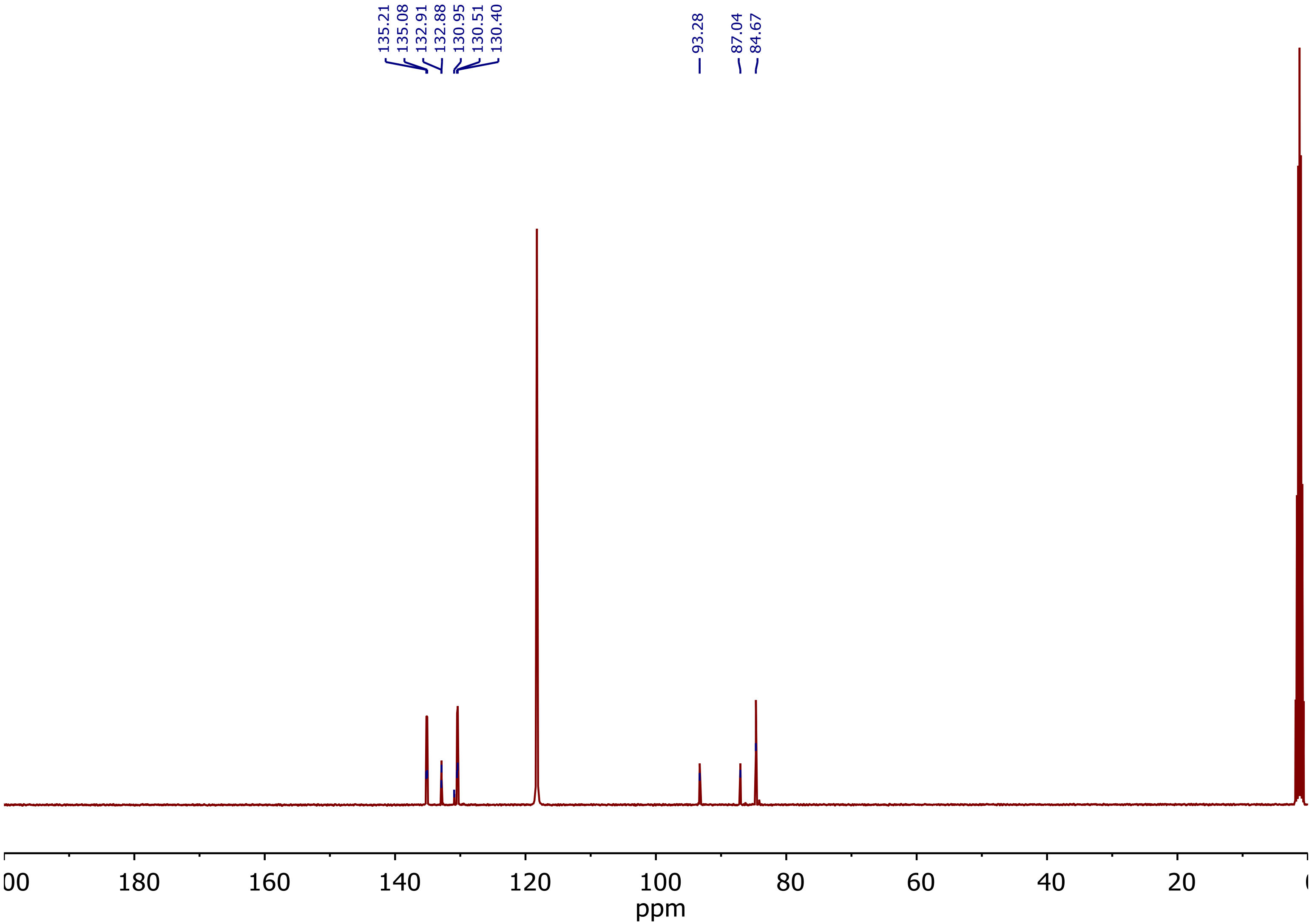

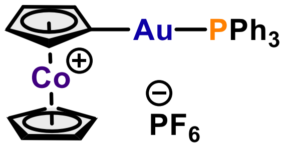

*Figure S 2: ^{13}C NMR (101 MHz, CD_3CN) **2a**.*

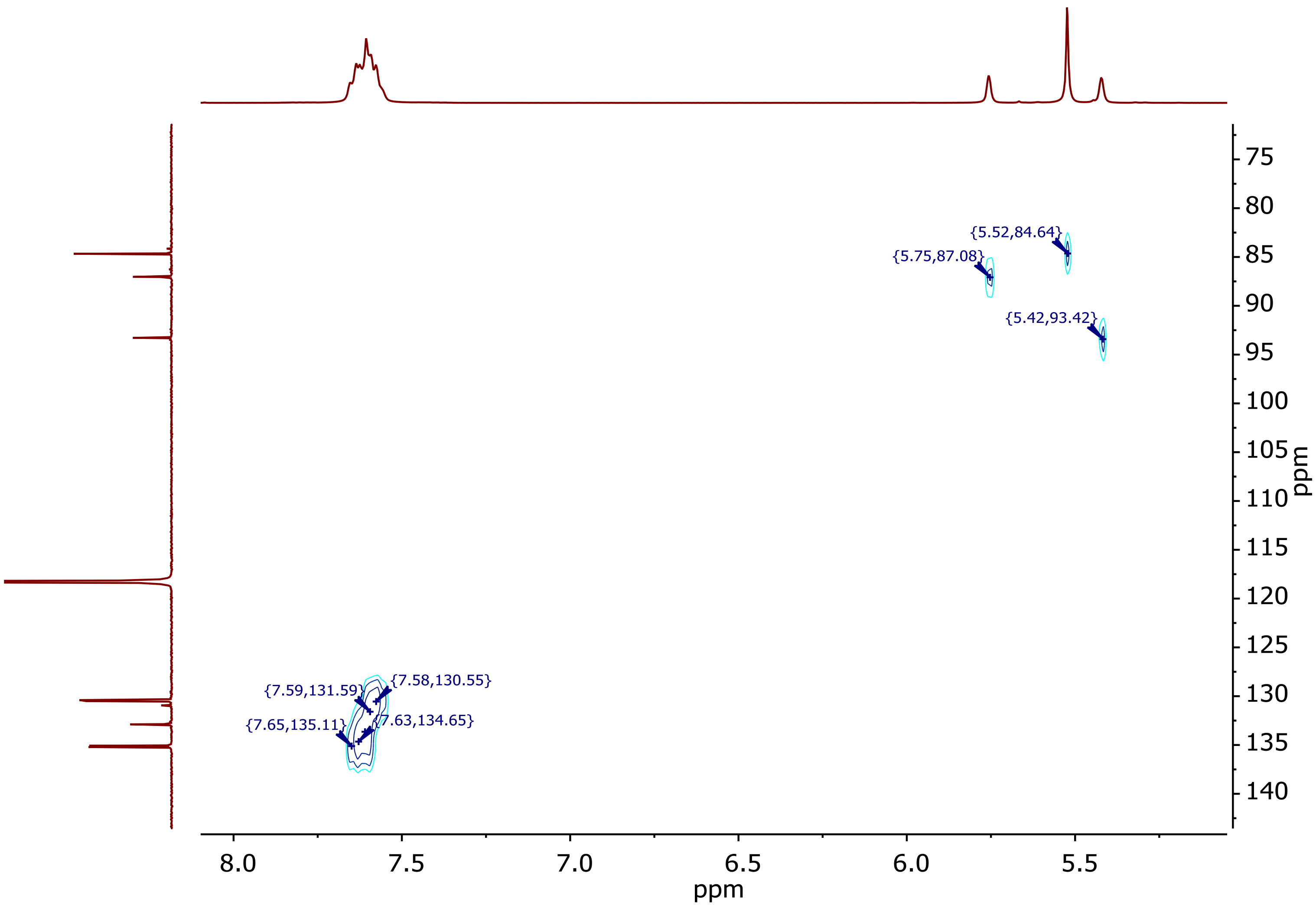

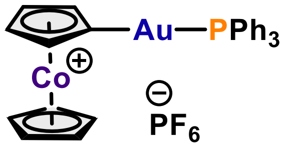

*Figure S 3: HSQC $^{1}H – ^{13}C$ (400 MHz, CD_3CN) **2a**.*

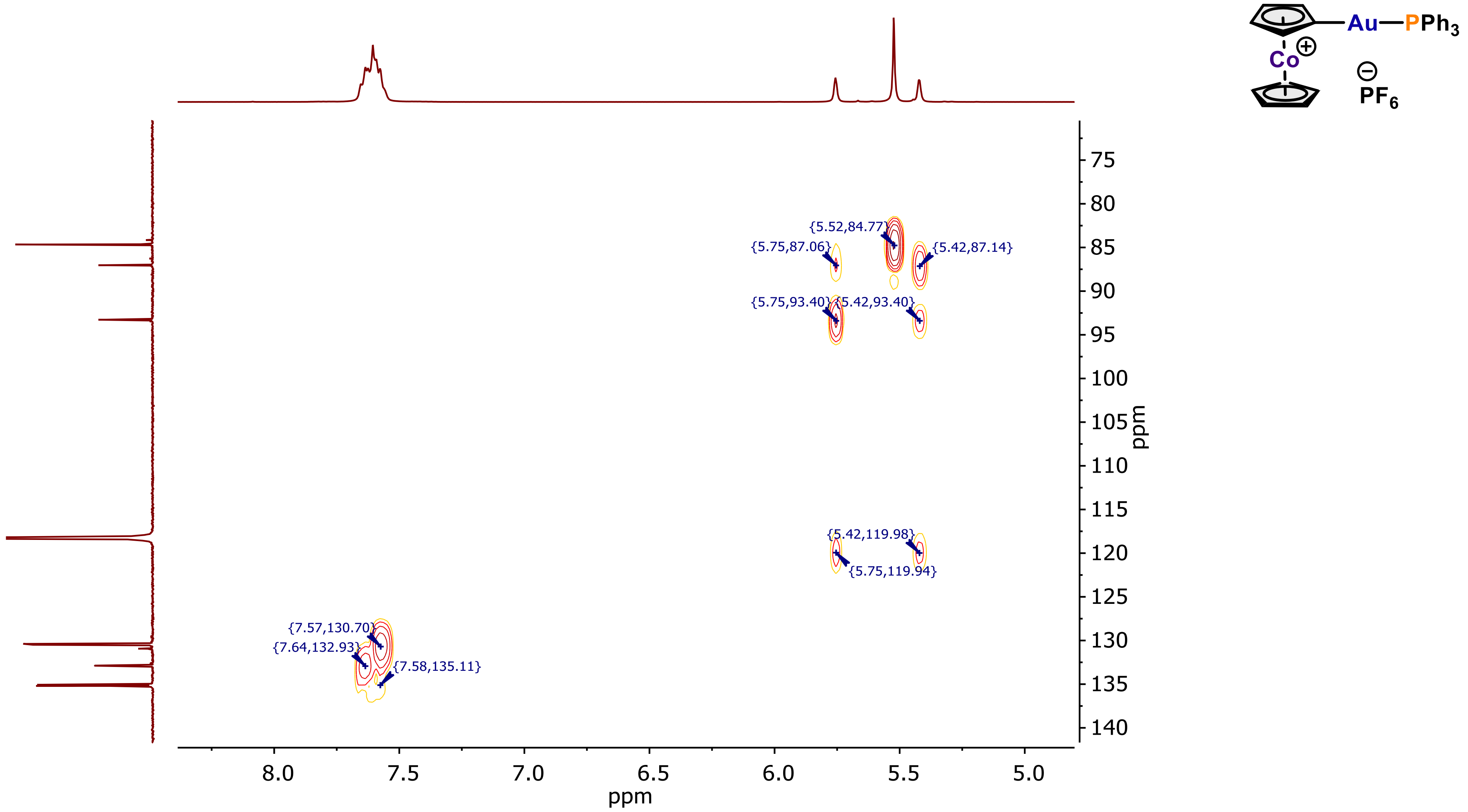

Figure S 4: HMBC ^{1}H – ^{13}C (400 MHz, CD_3CN) **2a**.

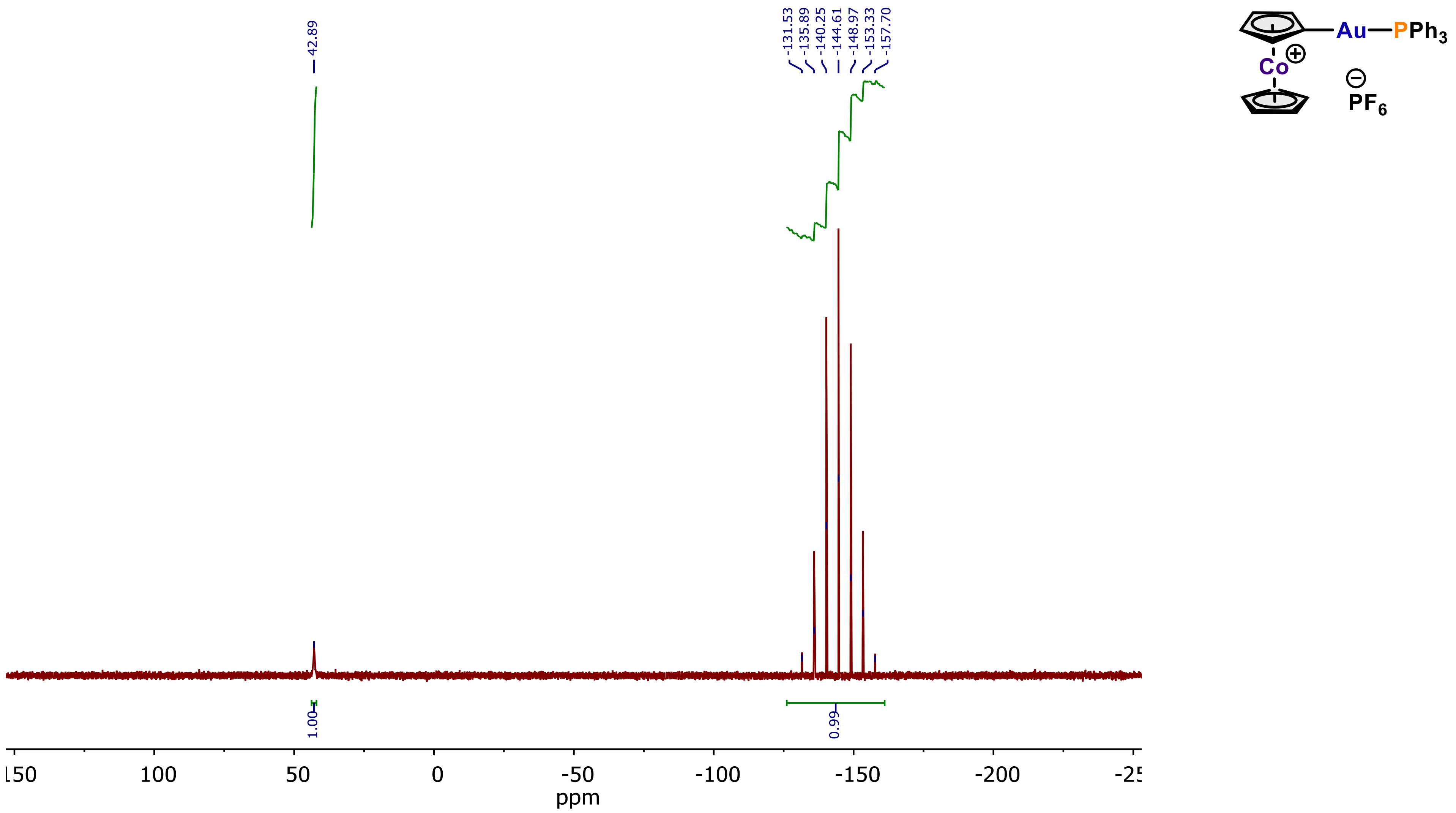

*Figure S 5: ^{31}P NMR (162 MHz, CD_3CN) **2a**.*

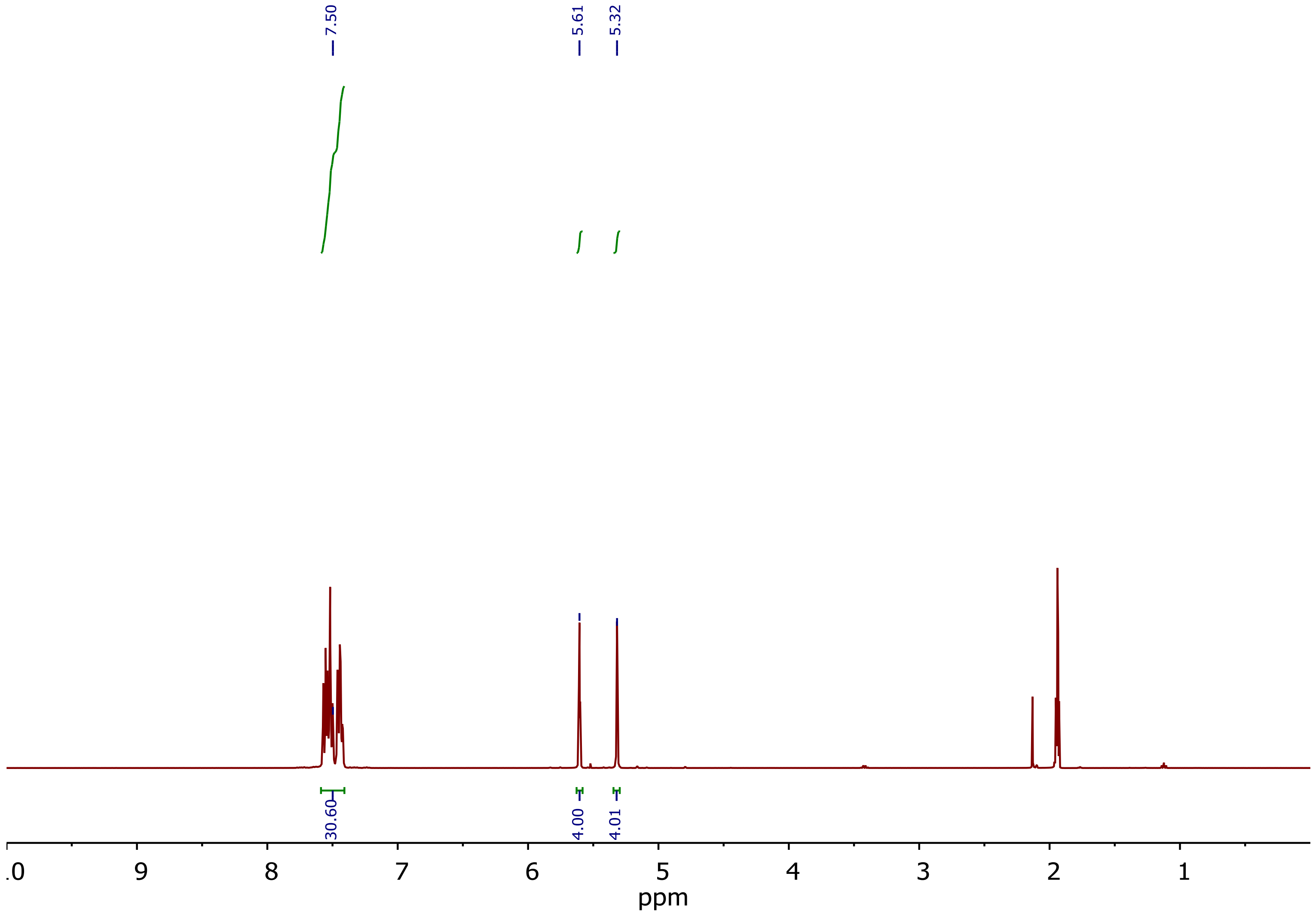

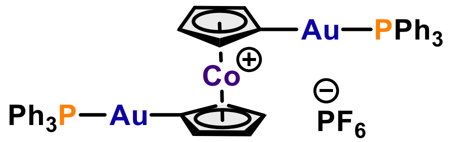

Figure S 6: ^{1}H NMR (400 MHz, CD_3CN) ***2b****.*

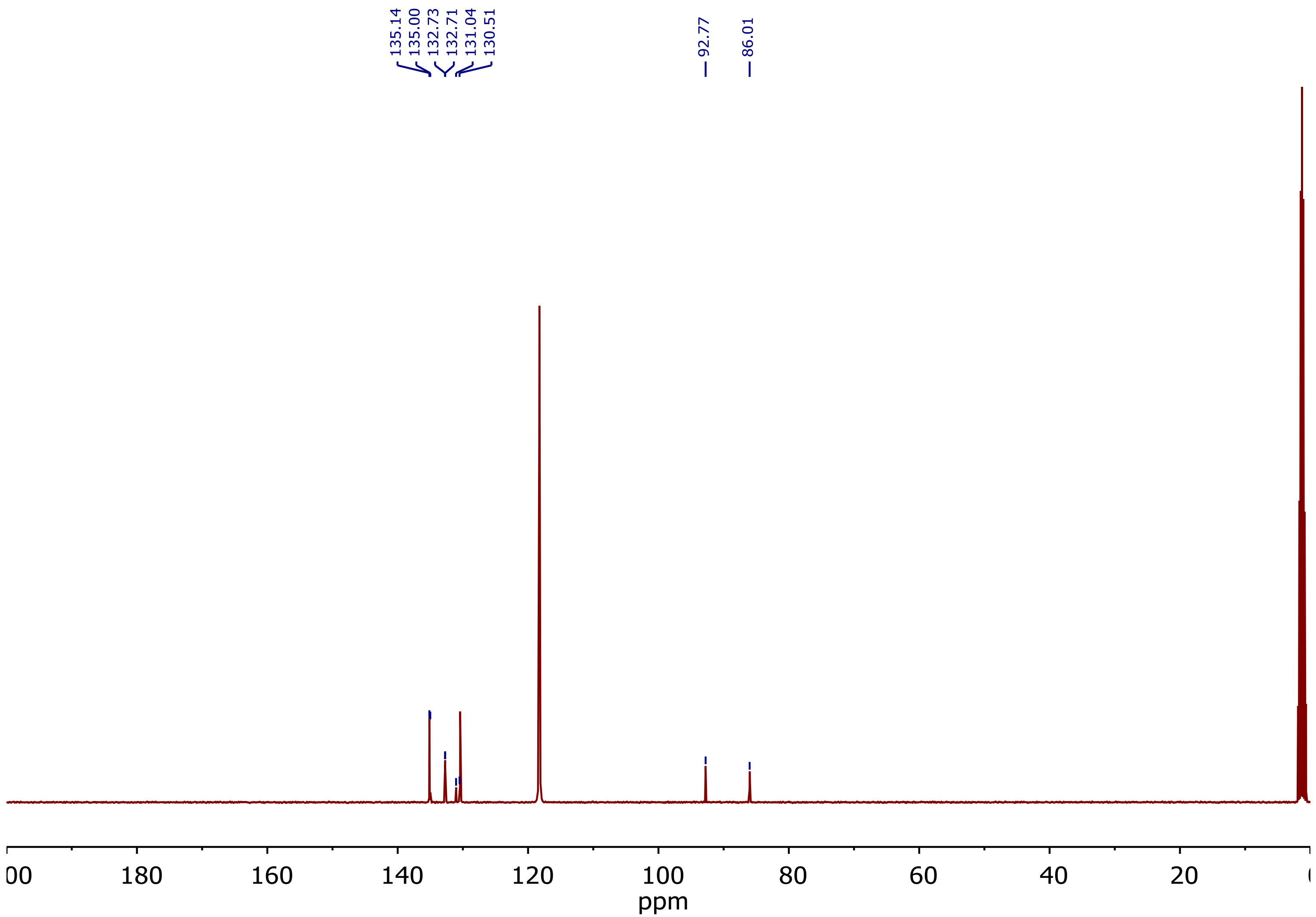

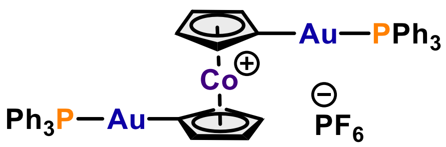

*Figure S 7: ^{13}C NMR (101 MHz, CD_3CN) **2b**.*

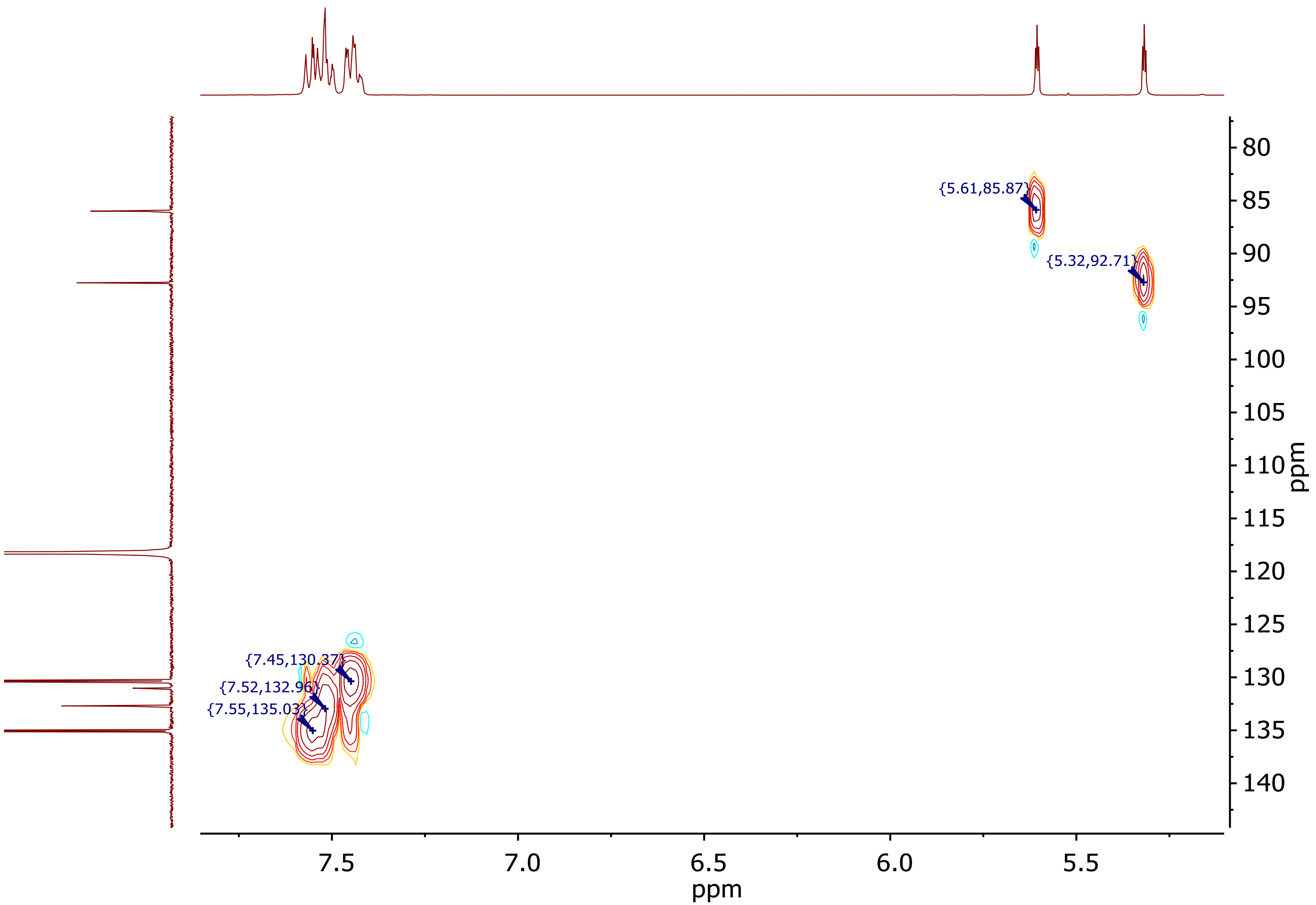

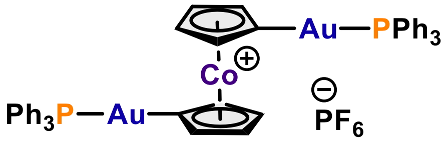

*Figure S 8: HSQC ^{1}H – ^{13}C (400 MHz, CD_3CN) **2b**.*

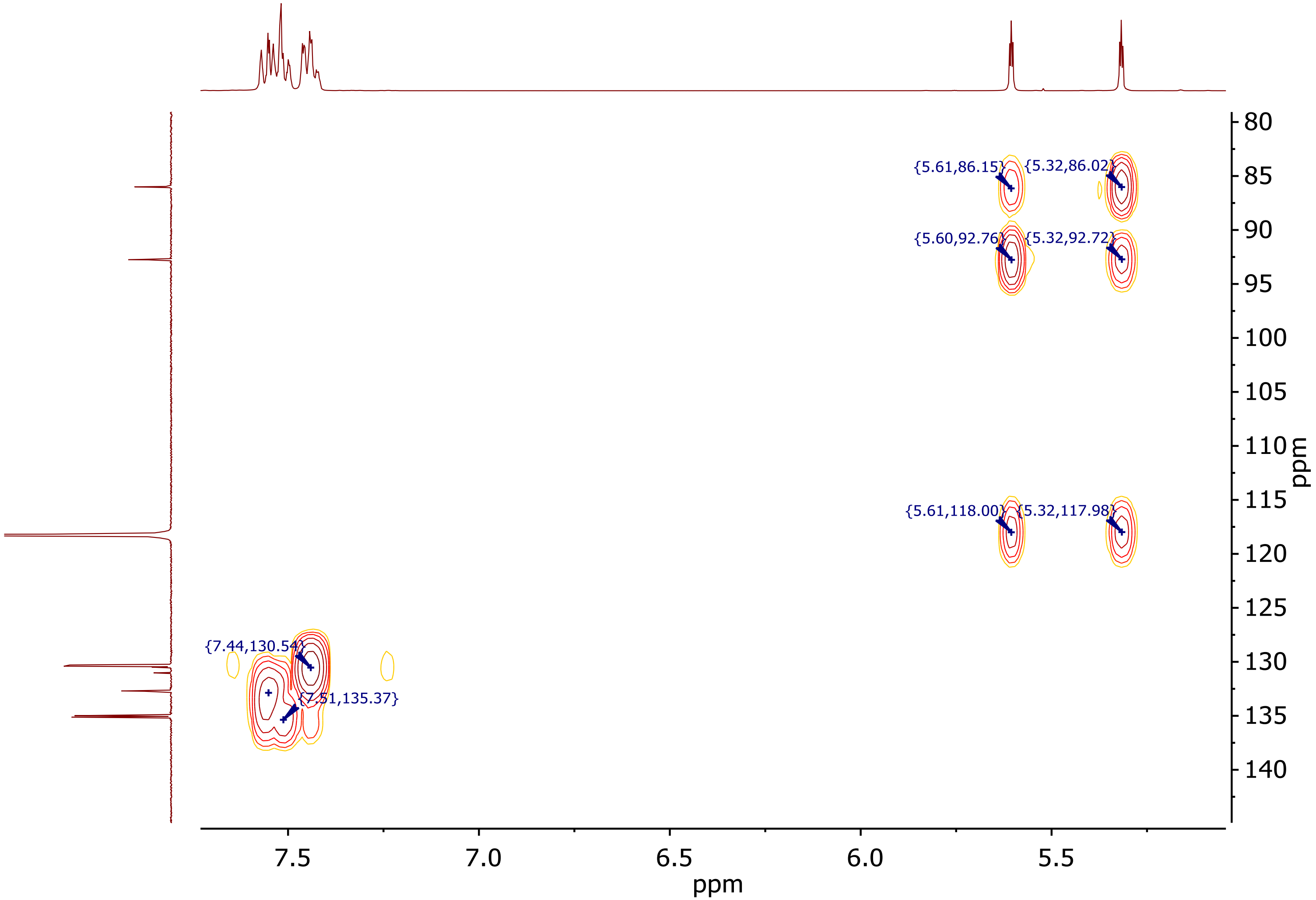

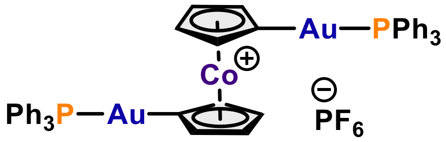

*Figure S 9: HMBC ^1H – ^{13}C (400 MHz, CD_3CN) **2b**.*

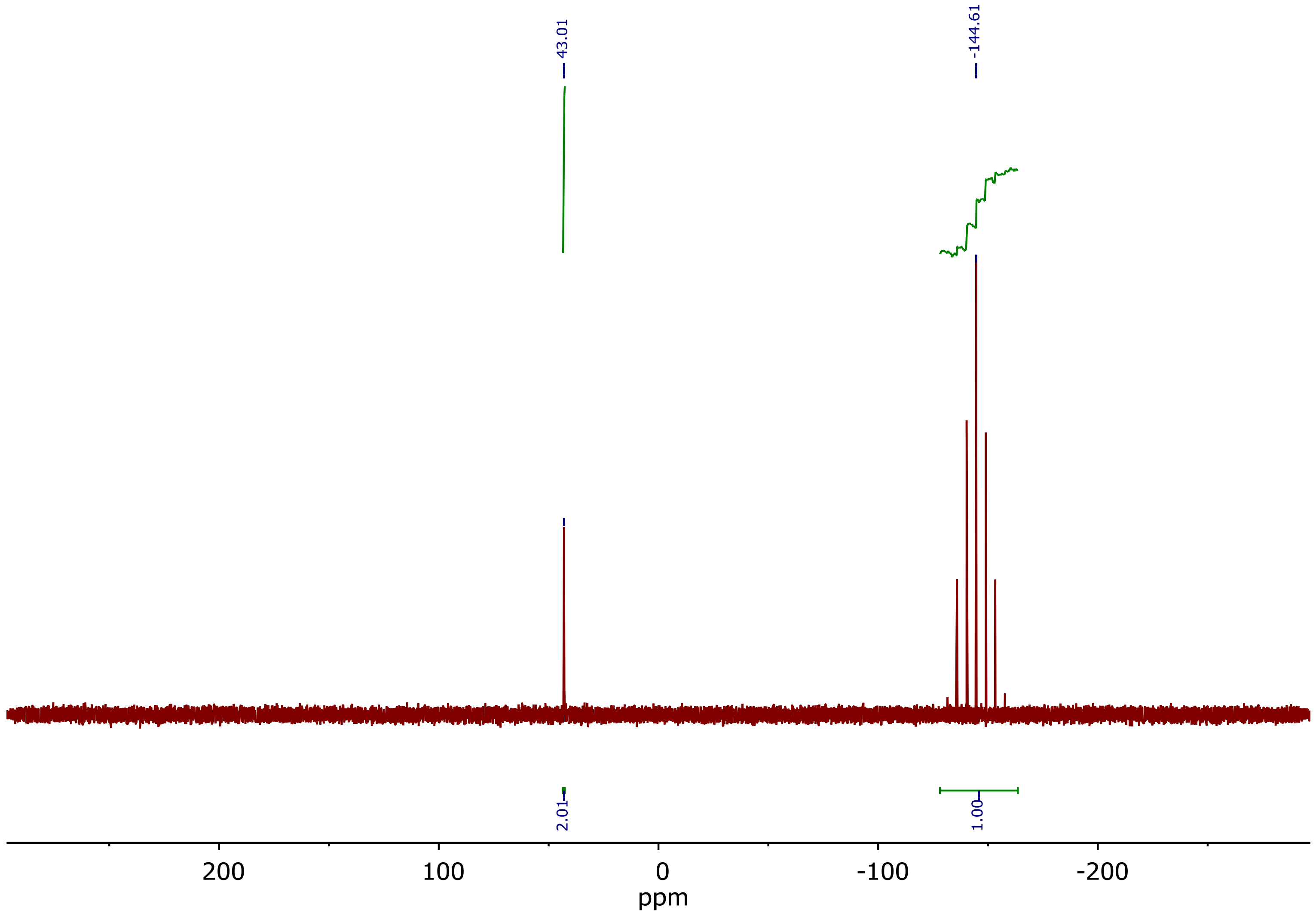

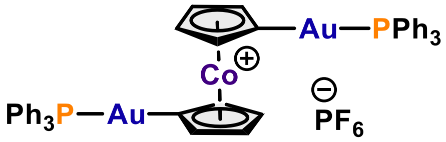

*Figure S 10: ^{31}P NMR (162 MHz, CD_3CN) **2b**.*

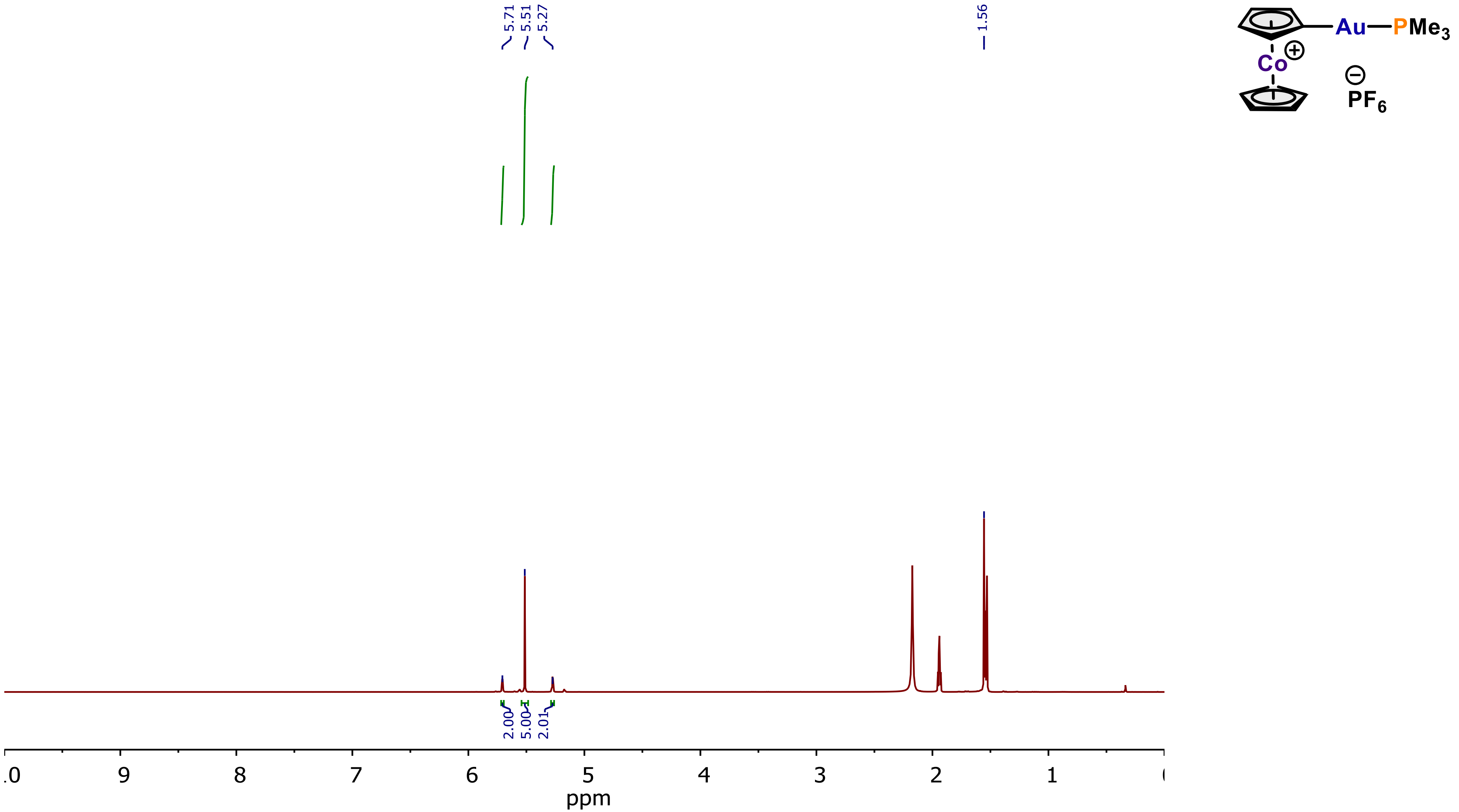

Figure S 11: ^{1}H NMR (400 MHz, CD_3CN) ***3a****. The spectrum contains minor impurities of* ***3b****. Furthermore, the methyl region of the PMe_3 ligand contains an unknown impurity for which this signal cannot be reliably integrated.*

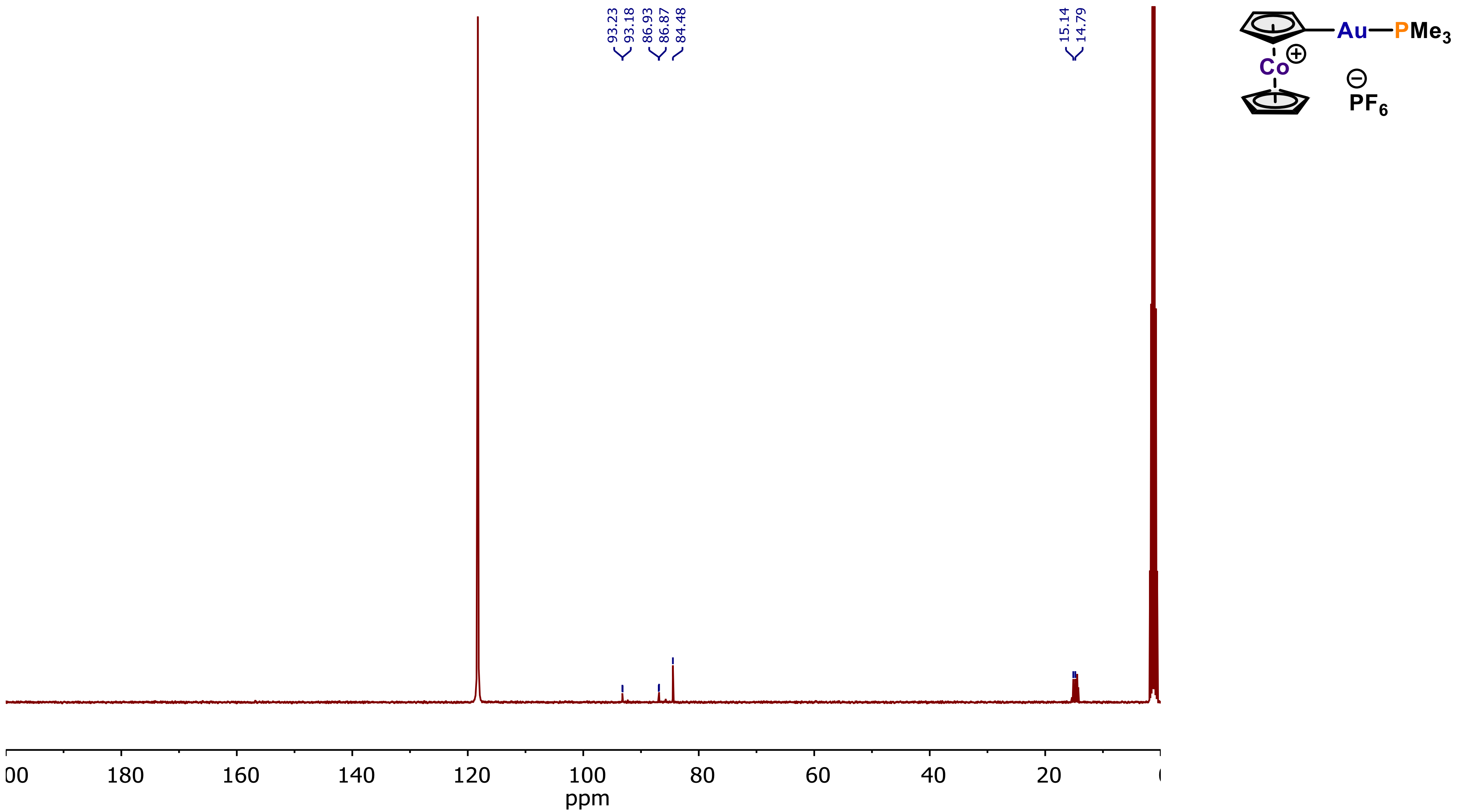

*Figure S 12: ^{13}C NMR (101 MHz, CD_3CN) **3a**. The spectrum contains minor impurities of **3b**.*

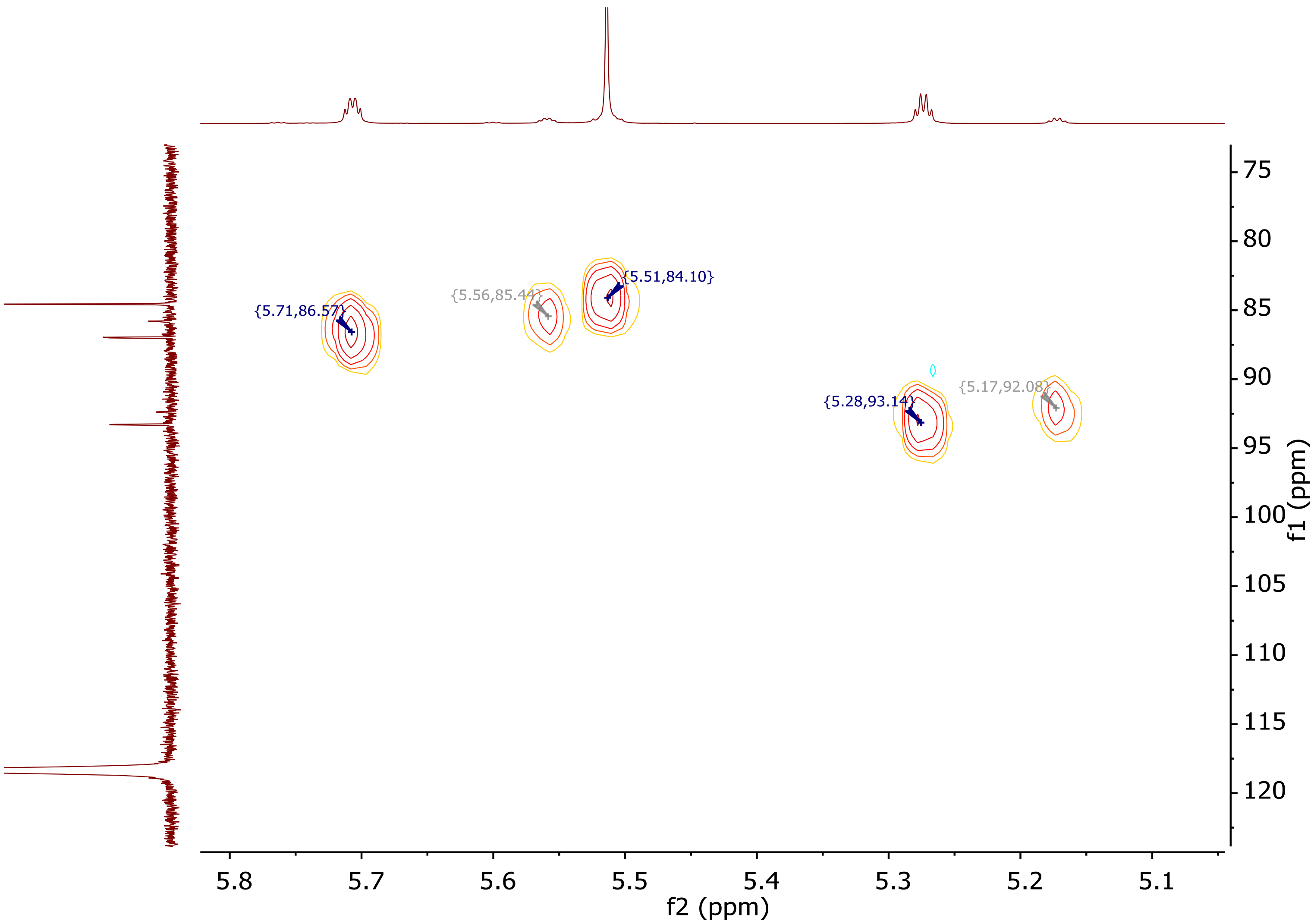

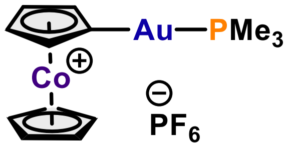

Figure S 13: HSQC ^{1}H – ^{13}C (400 MHz, CD_3CN) ***3a***.

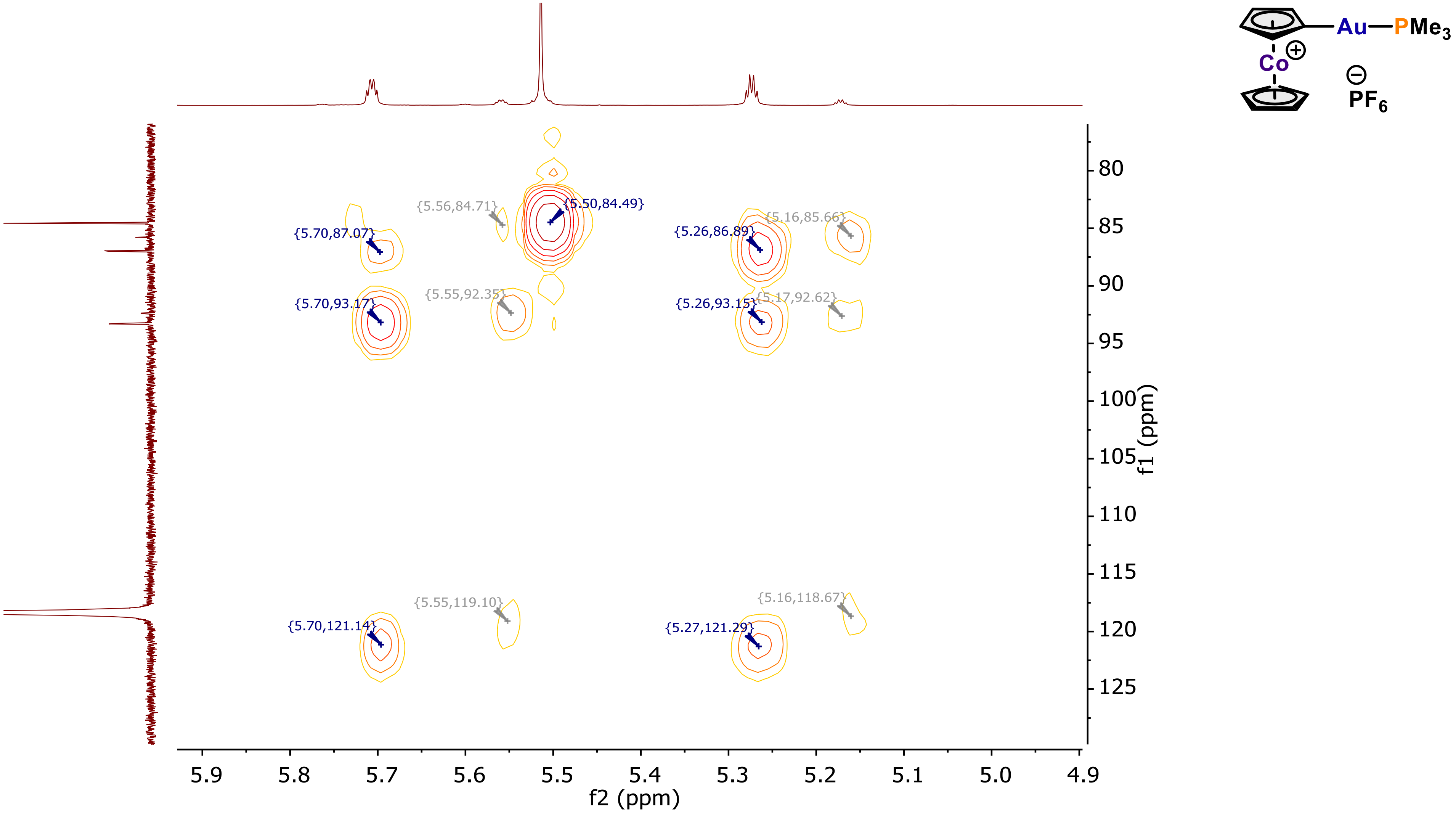

Figure S 14: HMBC ^{1}H – ^{13}C (400 MHz, CD_3CN) ***3a****.*

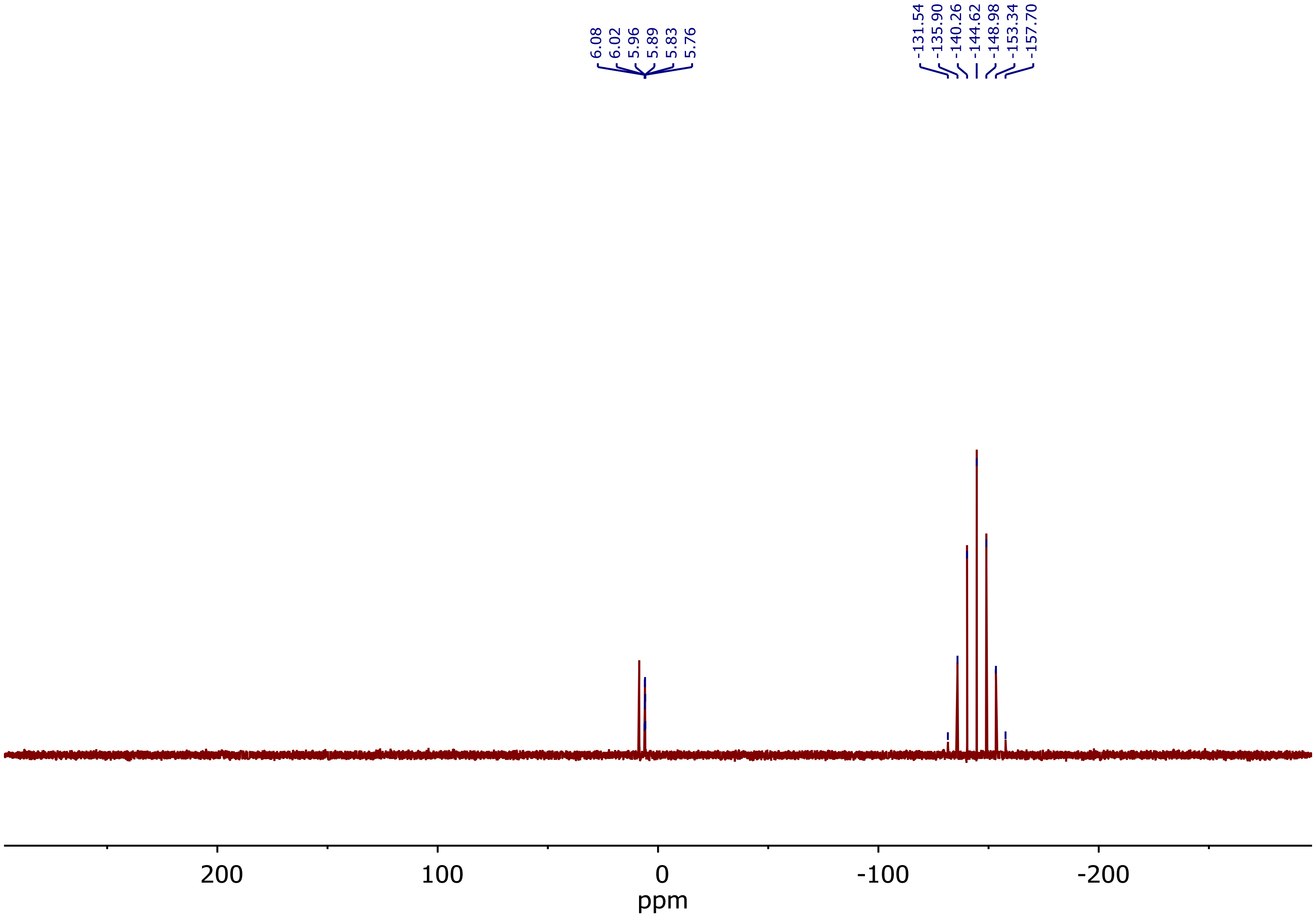

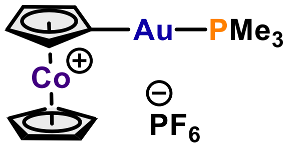

Figure S 15: ^{31}P NMR (162 MHz, CD_3CN) ***3a****. The spectrum contains impurities of* ***3b****.*

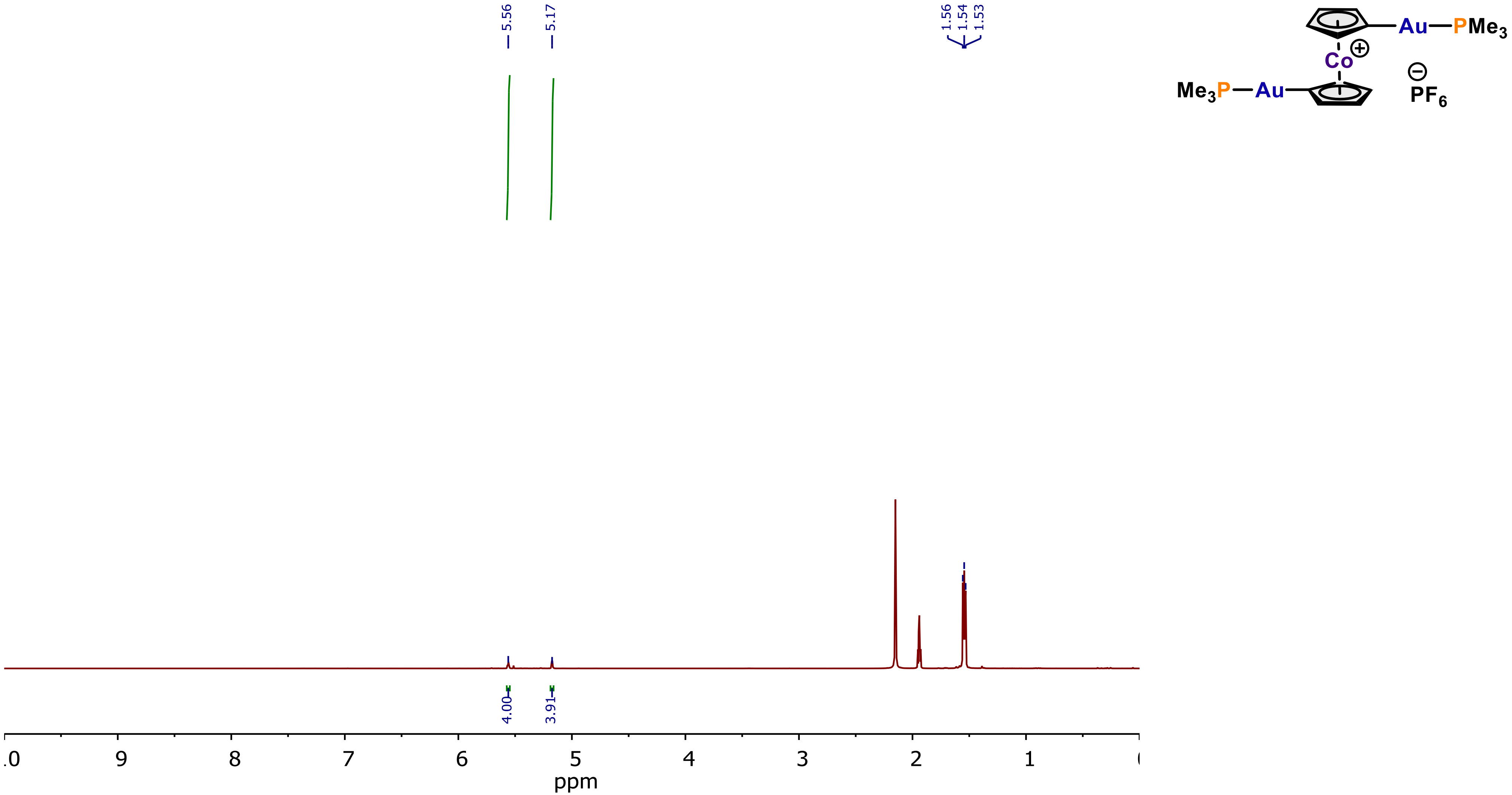

Figure S 16: ¹H NMR (400 MHz, CD_3CN) ***3b****. The spectrum contains a minor impurity of* ***3a****. Furthermore, the methyl region of the PMe_3 ligand contains an unknown impurity for which this signal cannot be reliably integrated.*

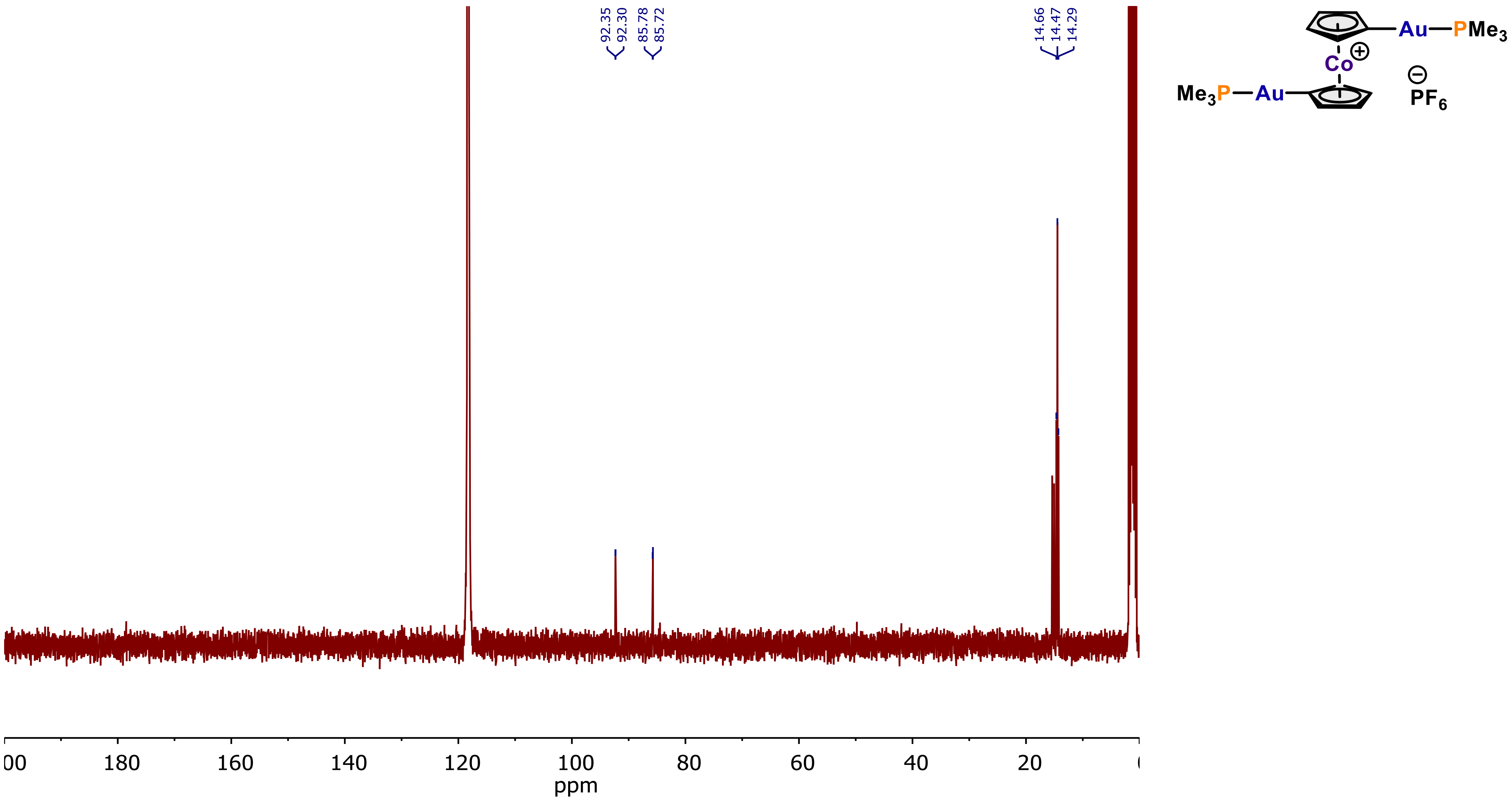

*Figure S 17: ^{13}C NMR (101 MHz, CD_3CN) **3b.** The spectrum contains minor impurities of **3a**.*

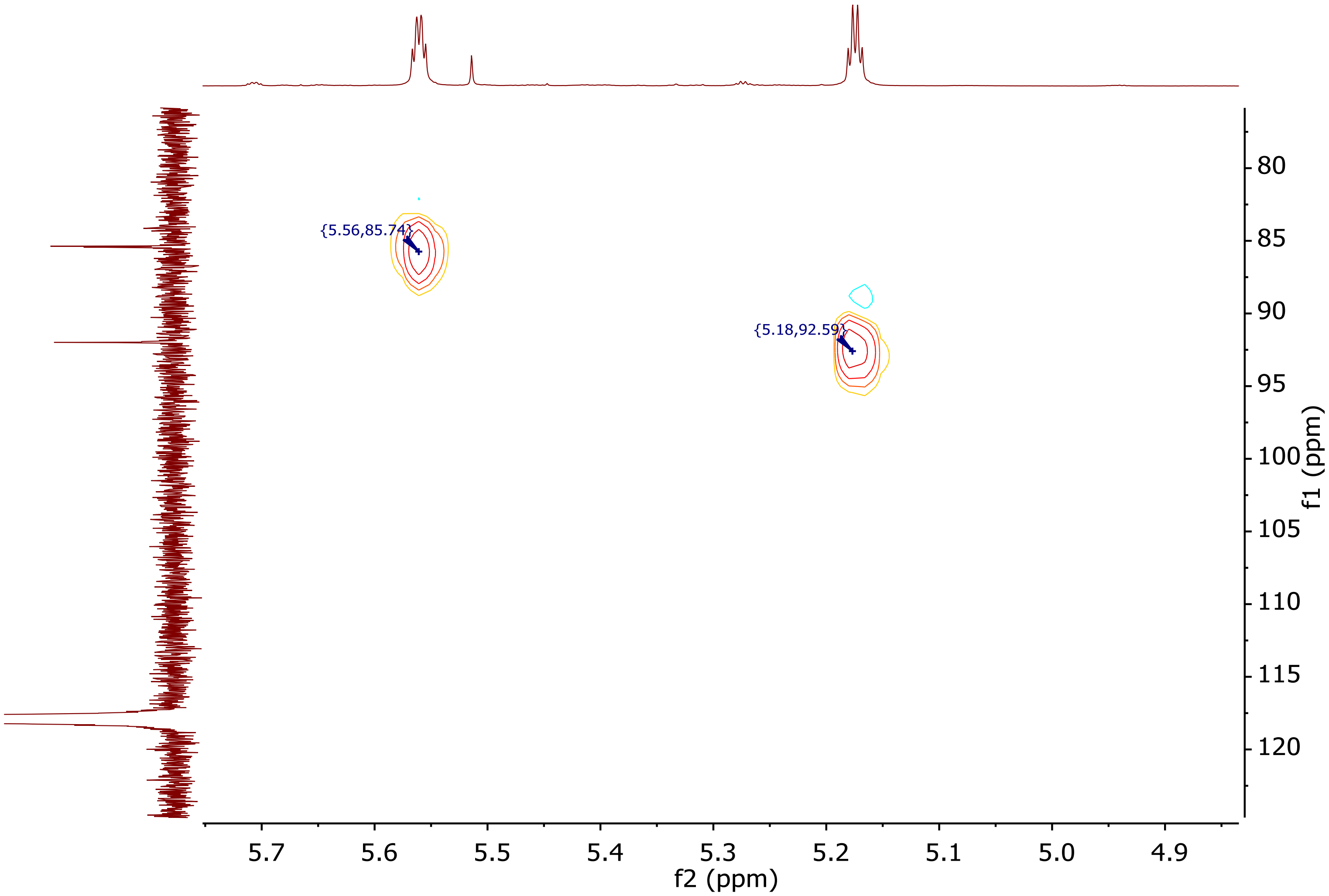

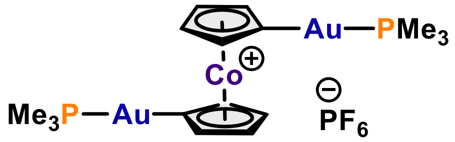

*Figure S 18: HSQC ^{1}H – ^{13}C (400 MHz, CD_3CN) **3b**.*

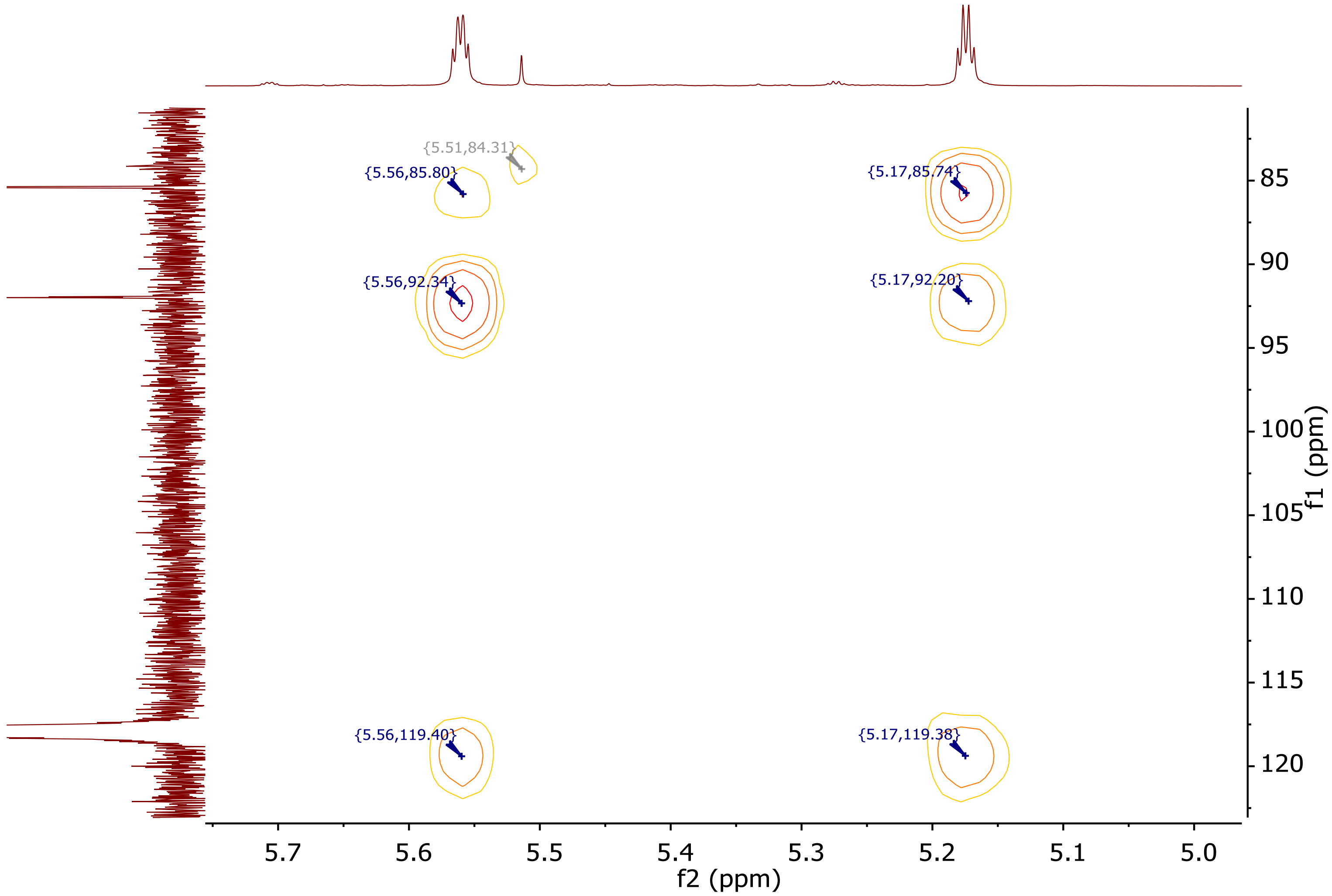

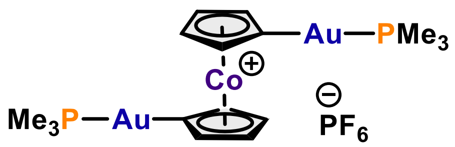

*Figure S 19: HMBC ^{1}H – ^{13}C (400 MHz, CD_3CN) **3b**.*

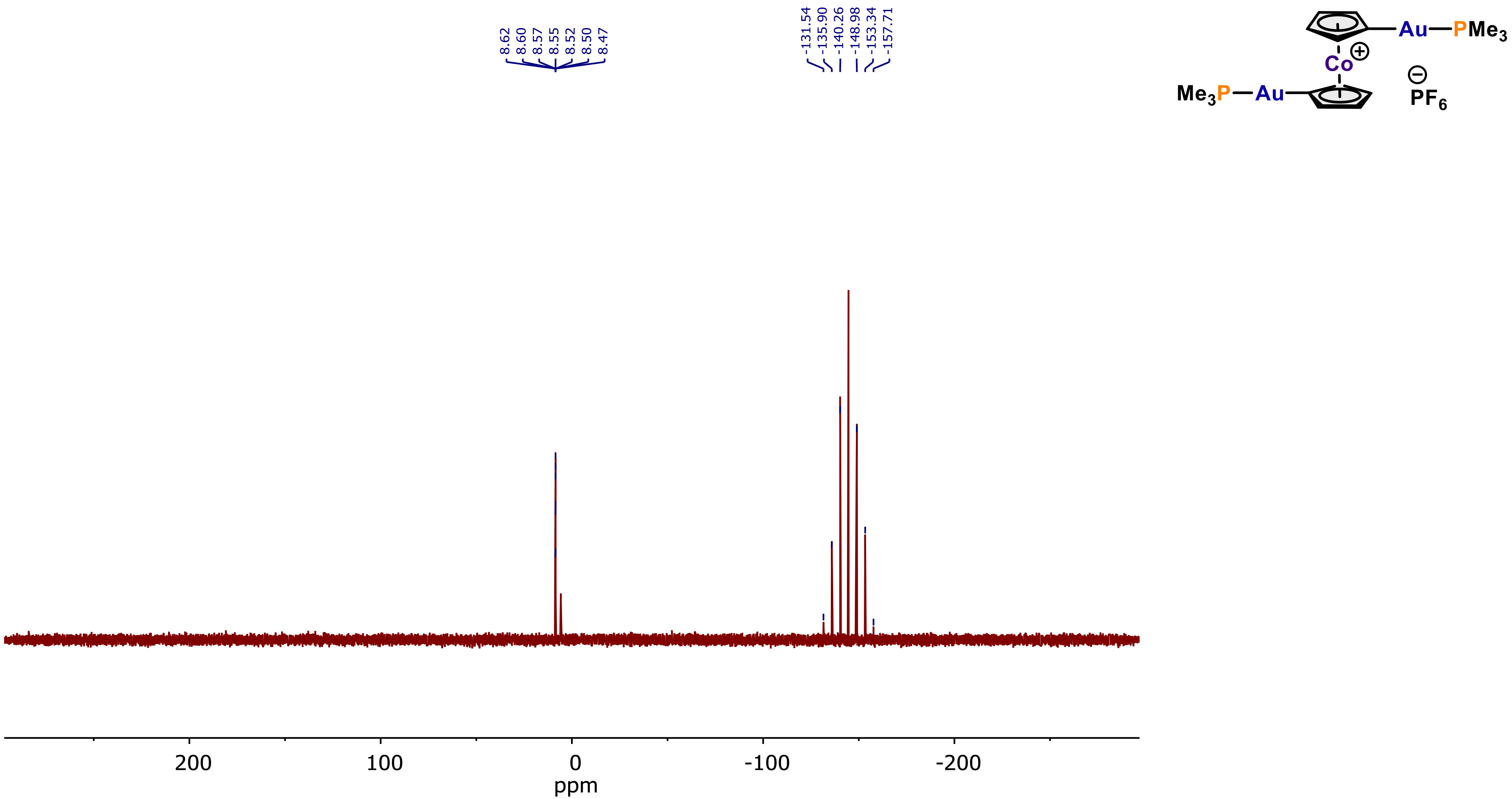

*Figure S 20: ^{31}P NMR (162 MHz, CD_3CN) **3b.** The spectrum contains minor impurities of **3a**.*

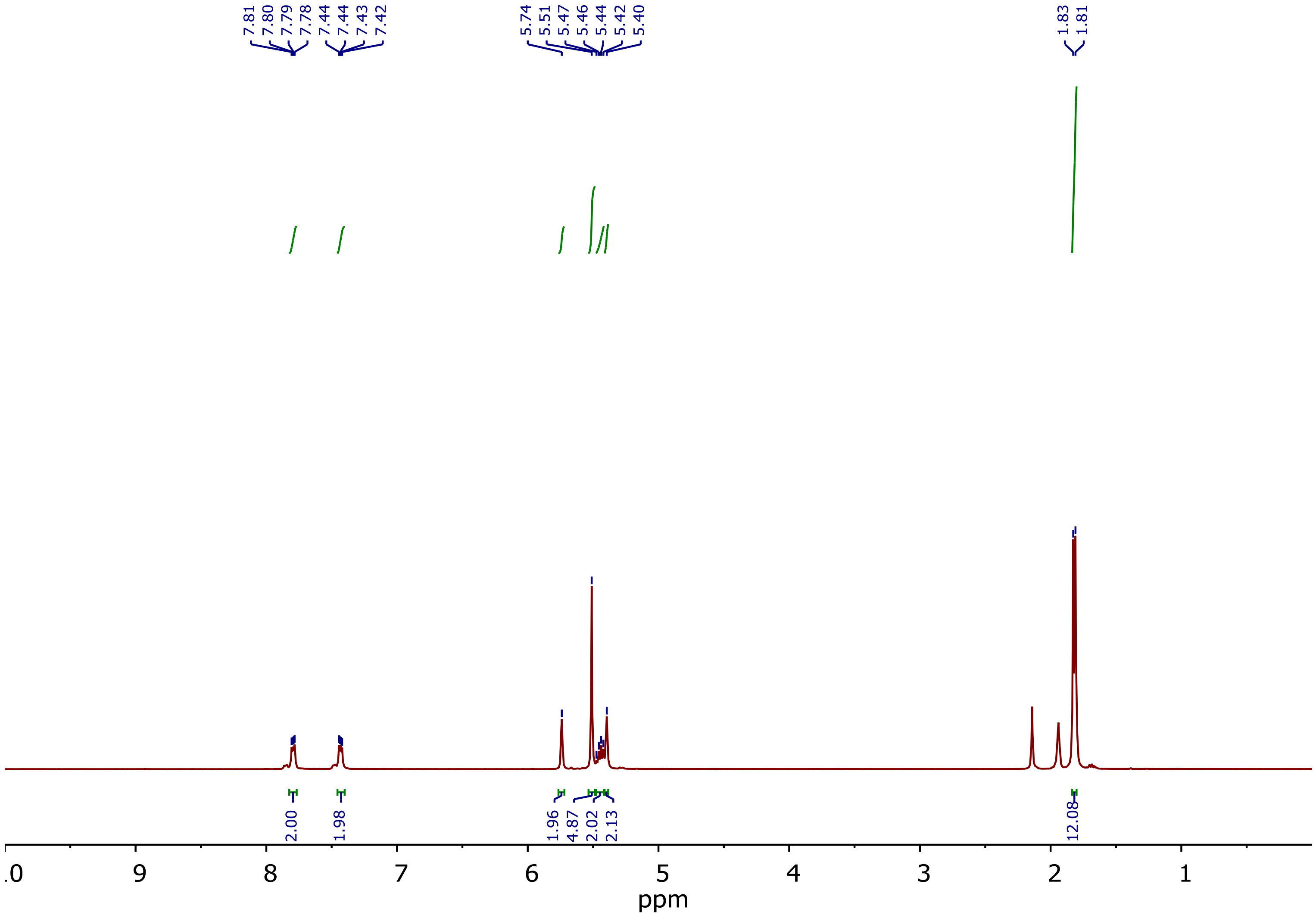

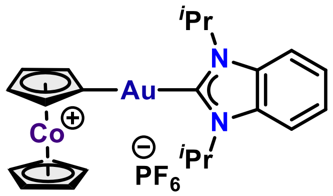

*Figure S 21: ^{1}H NMR (400 MHz, CD_3CN) **4a**.*

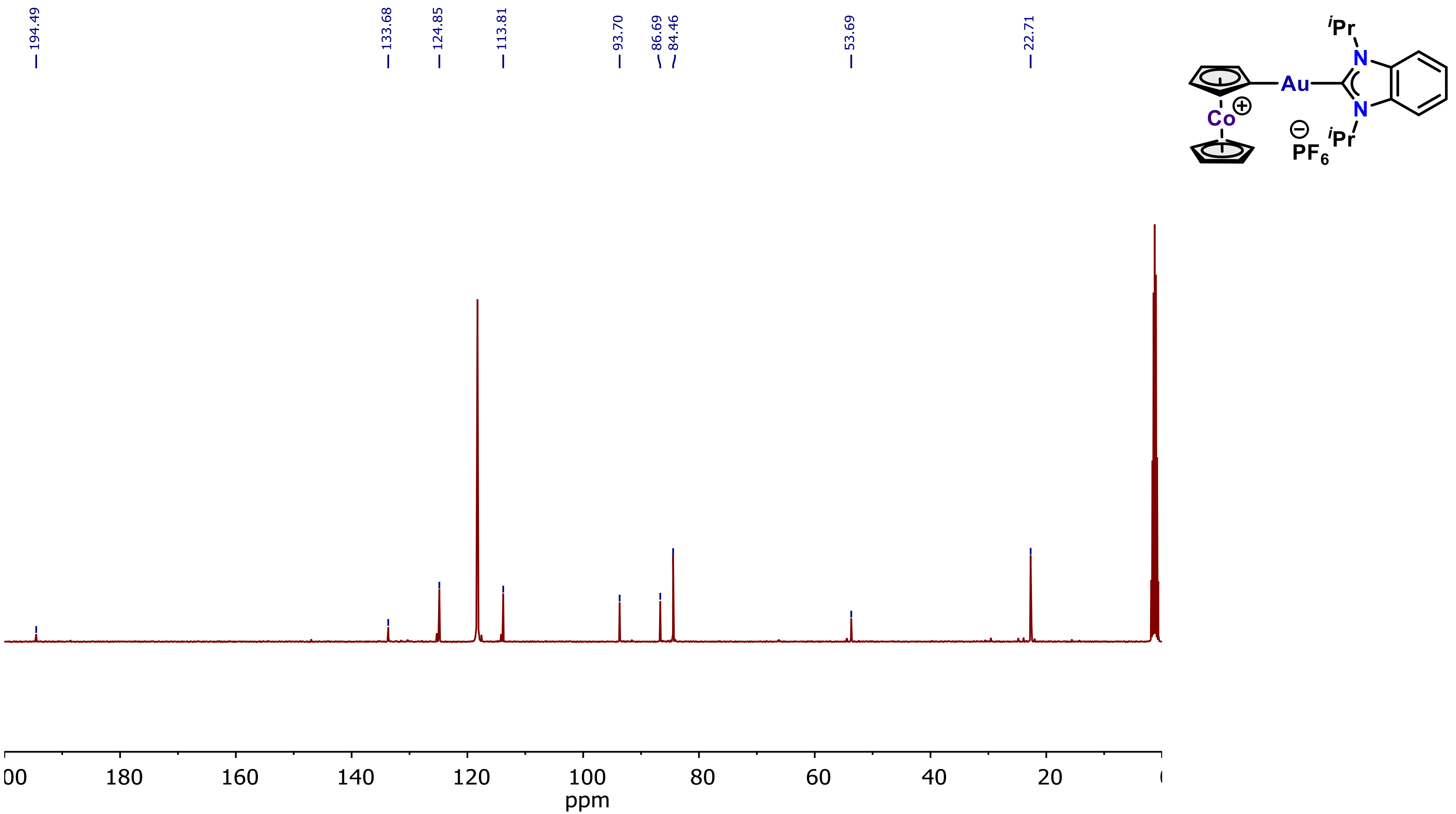

*Figure S 22: ^{13}C NMR (101 MHz, CD_3CN) **4a**.*

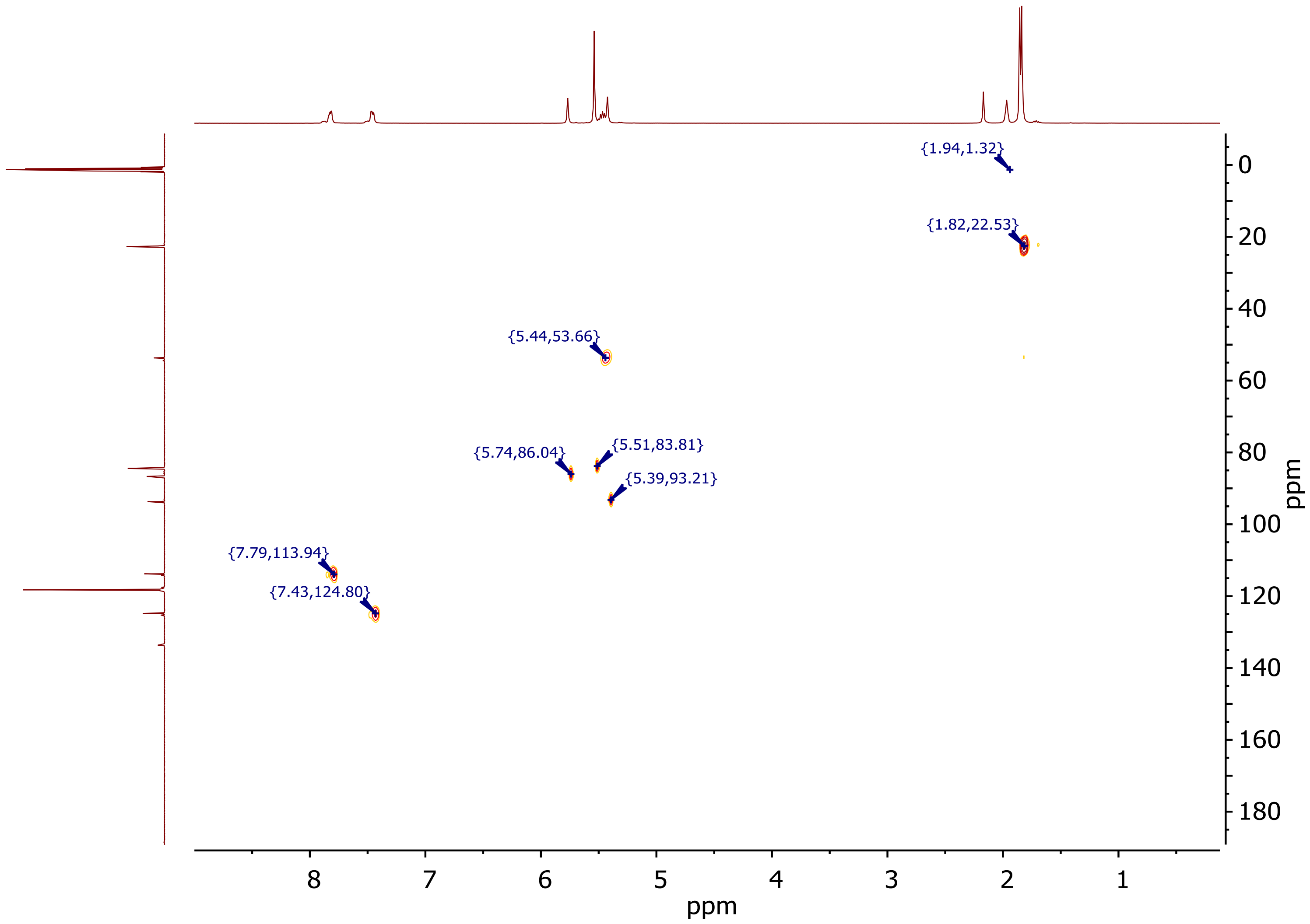

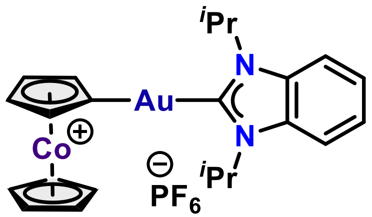

Figure S 23: HSQC ^1H – ^{13}C (400 MHz, CD_3CN) **4a**.

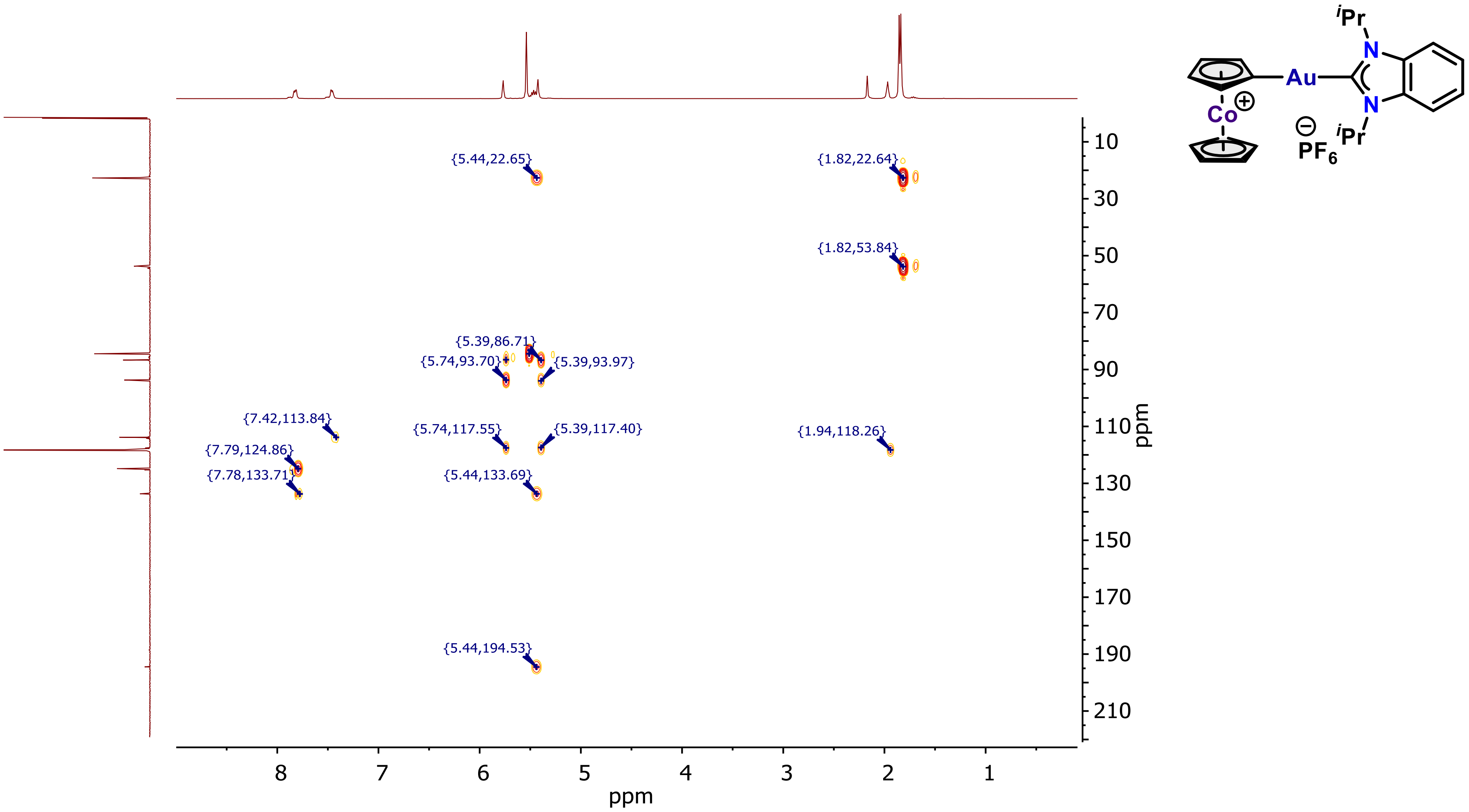

*Figure S 24: HMBC ^1H – ^{13}C (400 MHz, CD_3CN) **4a**.*

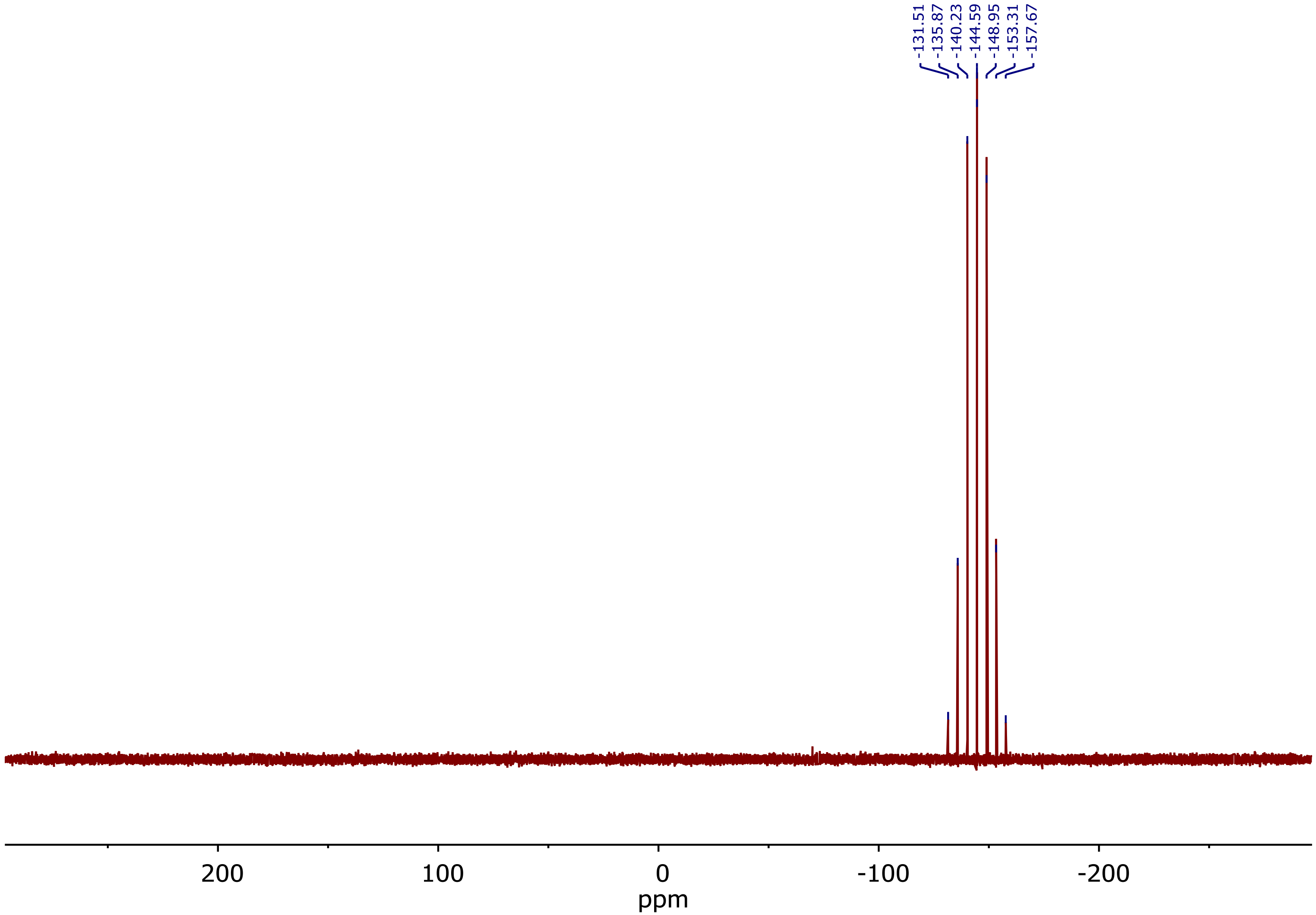

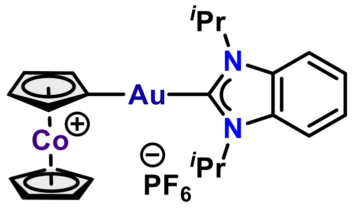

*Figure S 25: ^{31}P NMR (162 MHz, CD_3CN) **4a**.*

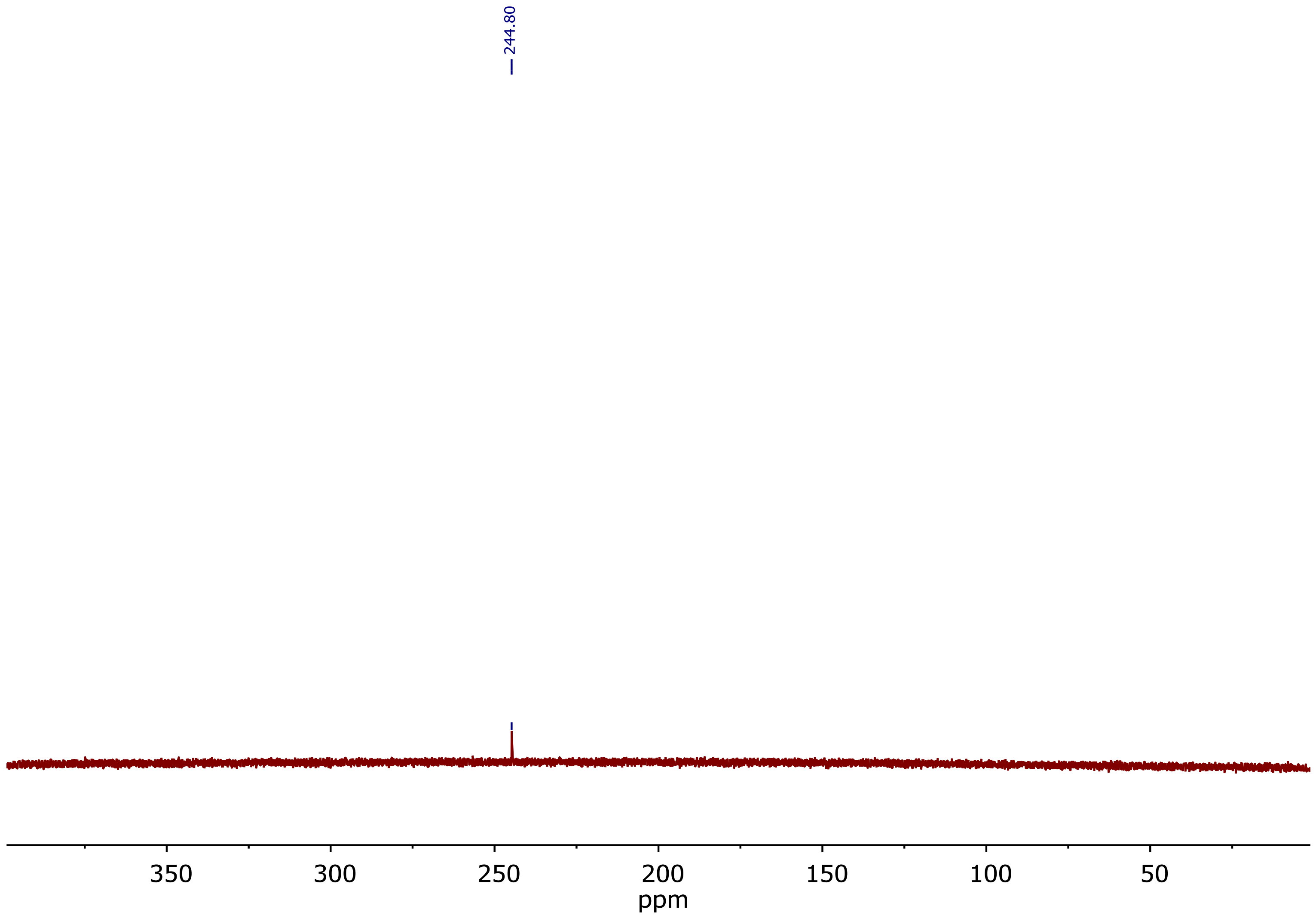

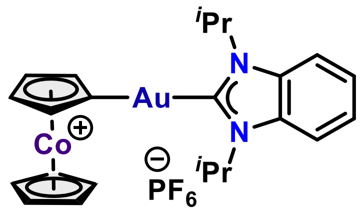

*Figure S 26: ^{15}N NMR (41 MHz, CD_3CN) **4a**.*

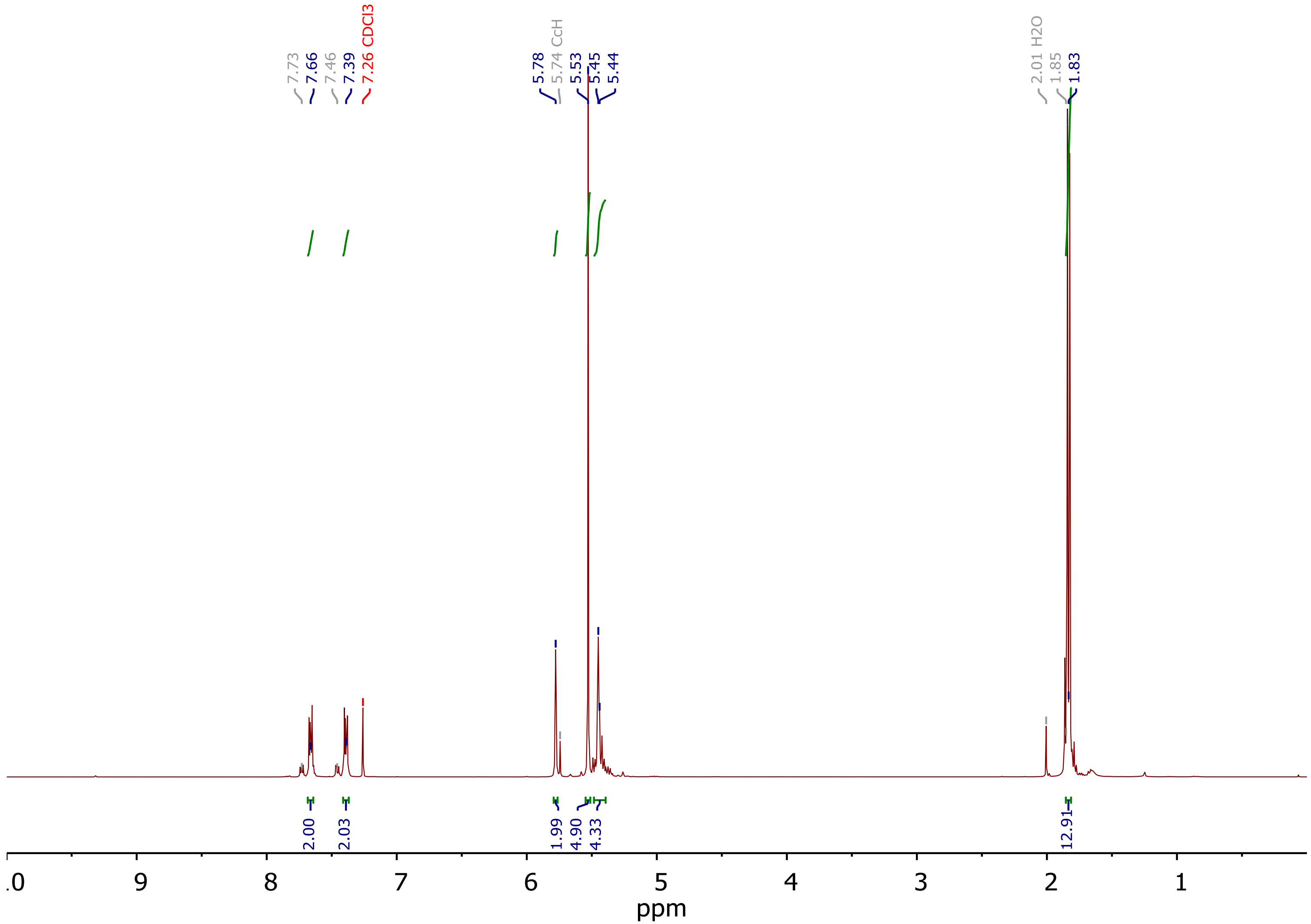

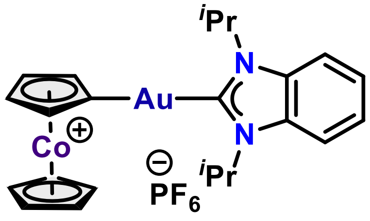

Figure S 27: ^{1}H NMR (400 MHz, $CDCl_3$) **4a**.

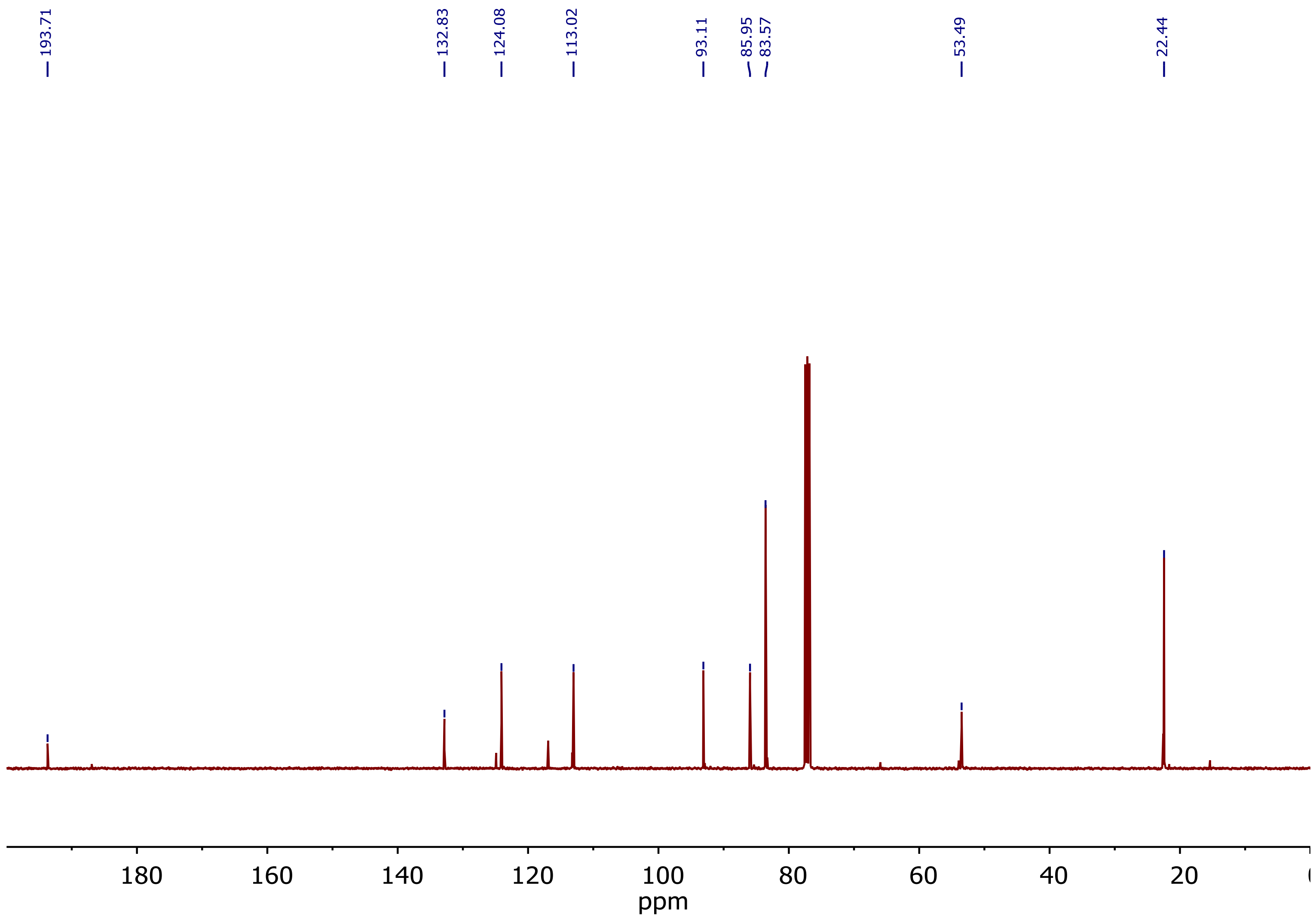

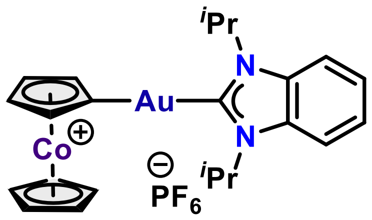

Figure S 28: ^{13}C NMR (101 MHz, CDCl$_3$) ***4a***.

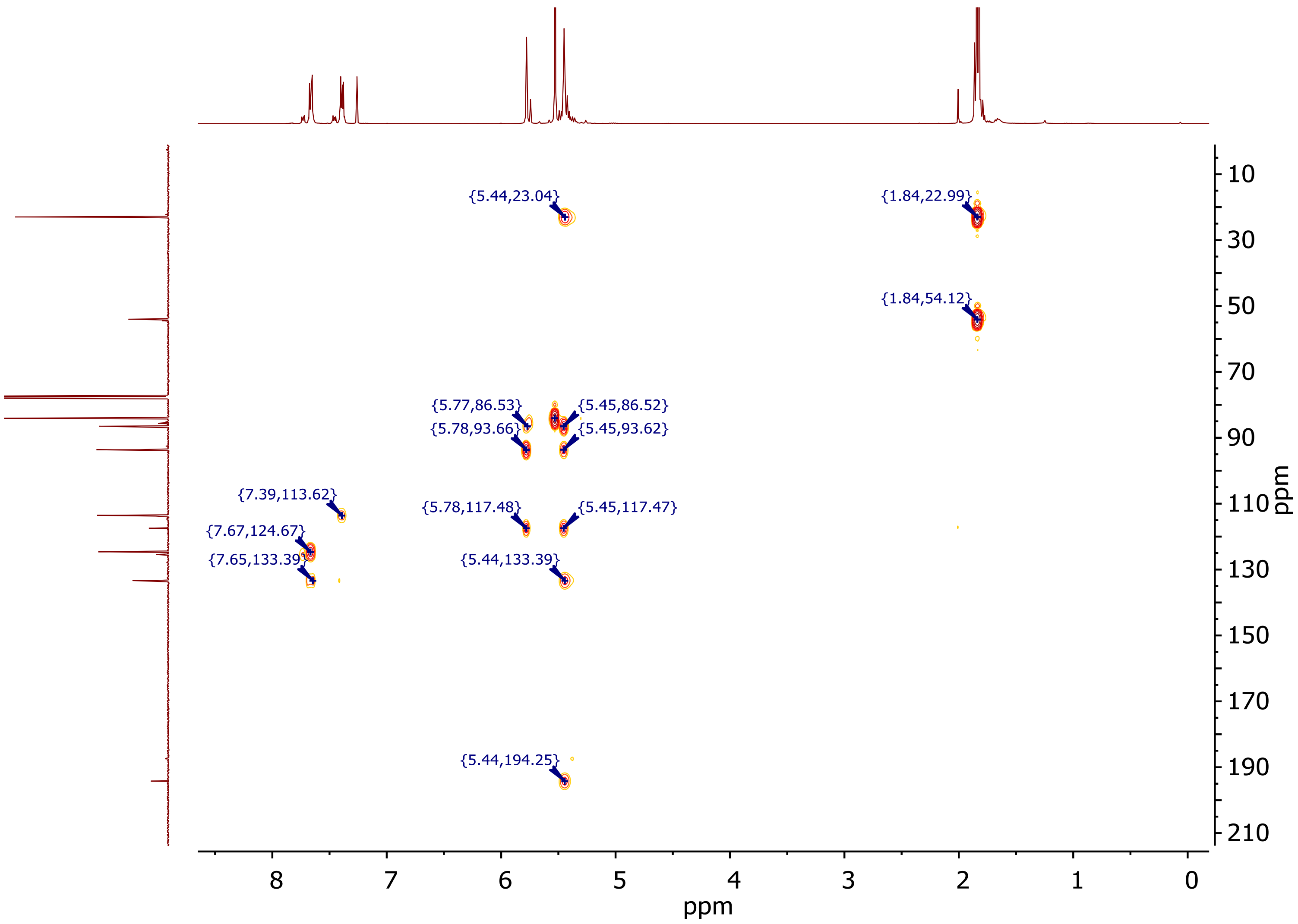

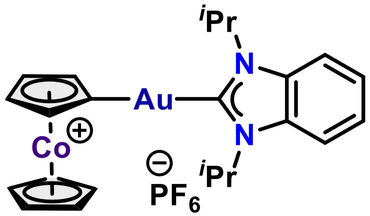

*Figure S 29: HMBC ^{1}H – ^{13}C (400 MHz, $CDCl_3$) **4a**.*

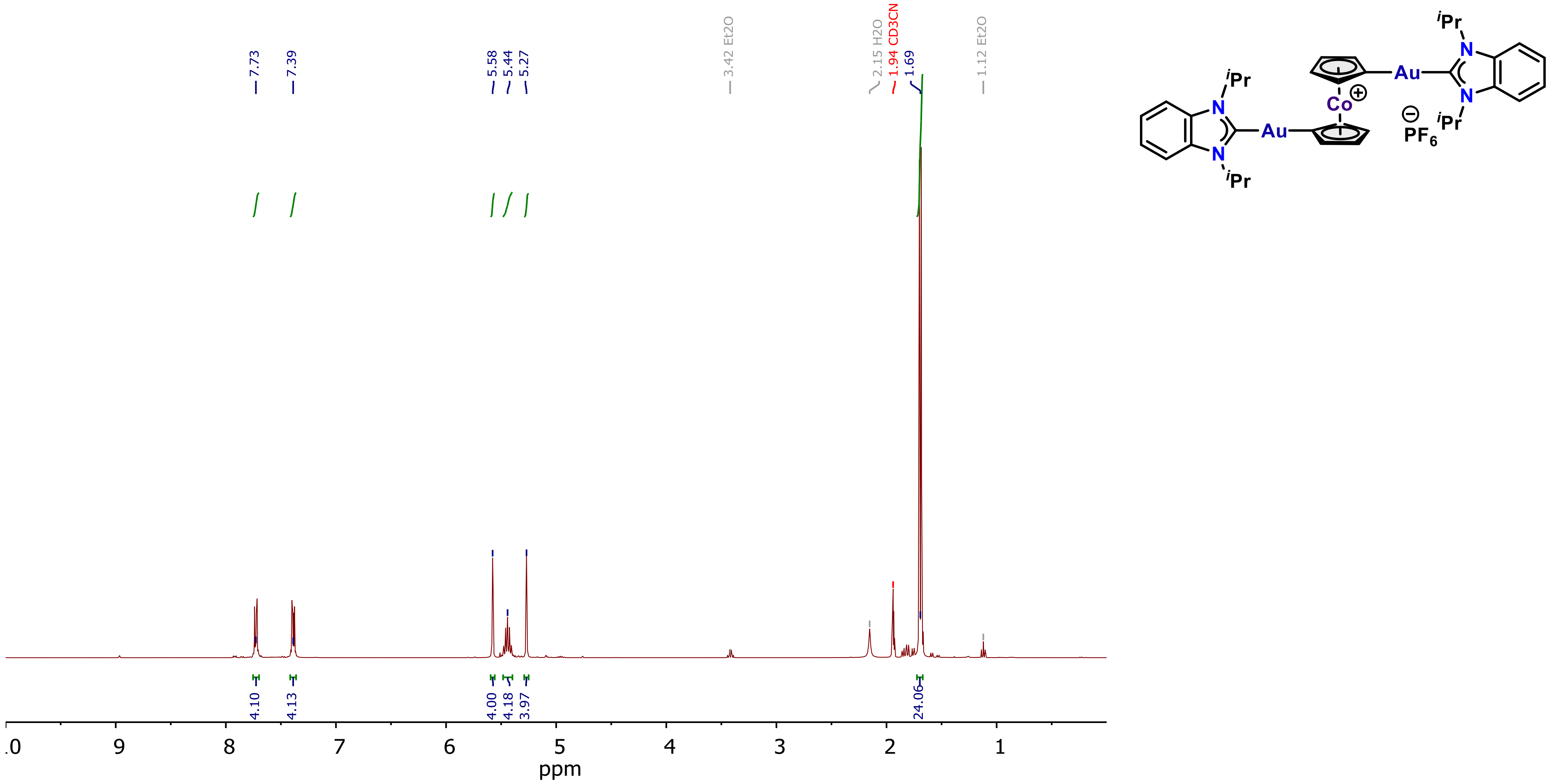

Figure S 30: ^{1}H NMR (400 MHz, CD_3CN) **4b**.

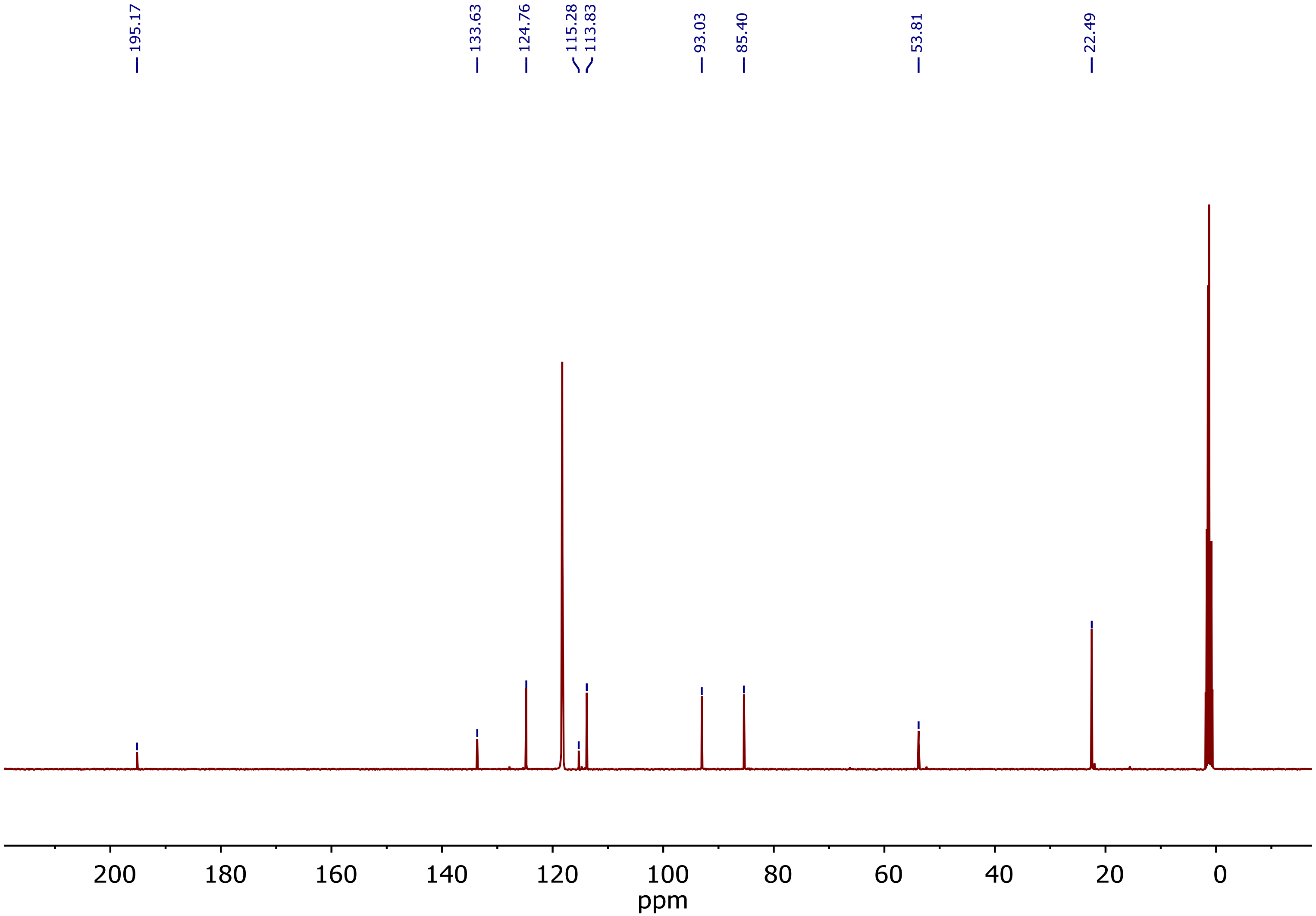

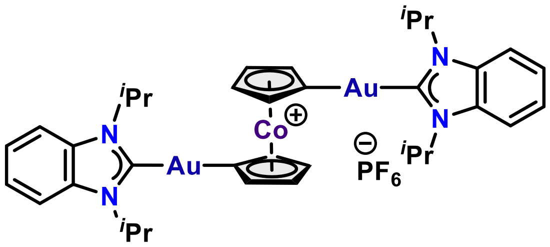

*Figure S 31: ^{13}C NMR (101 MHz, CD_3CN) **4b**.*

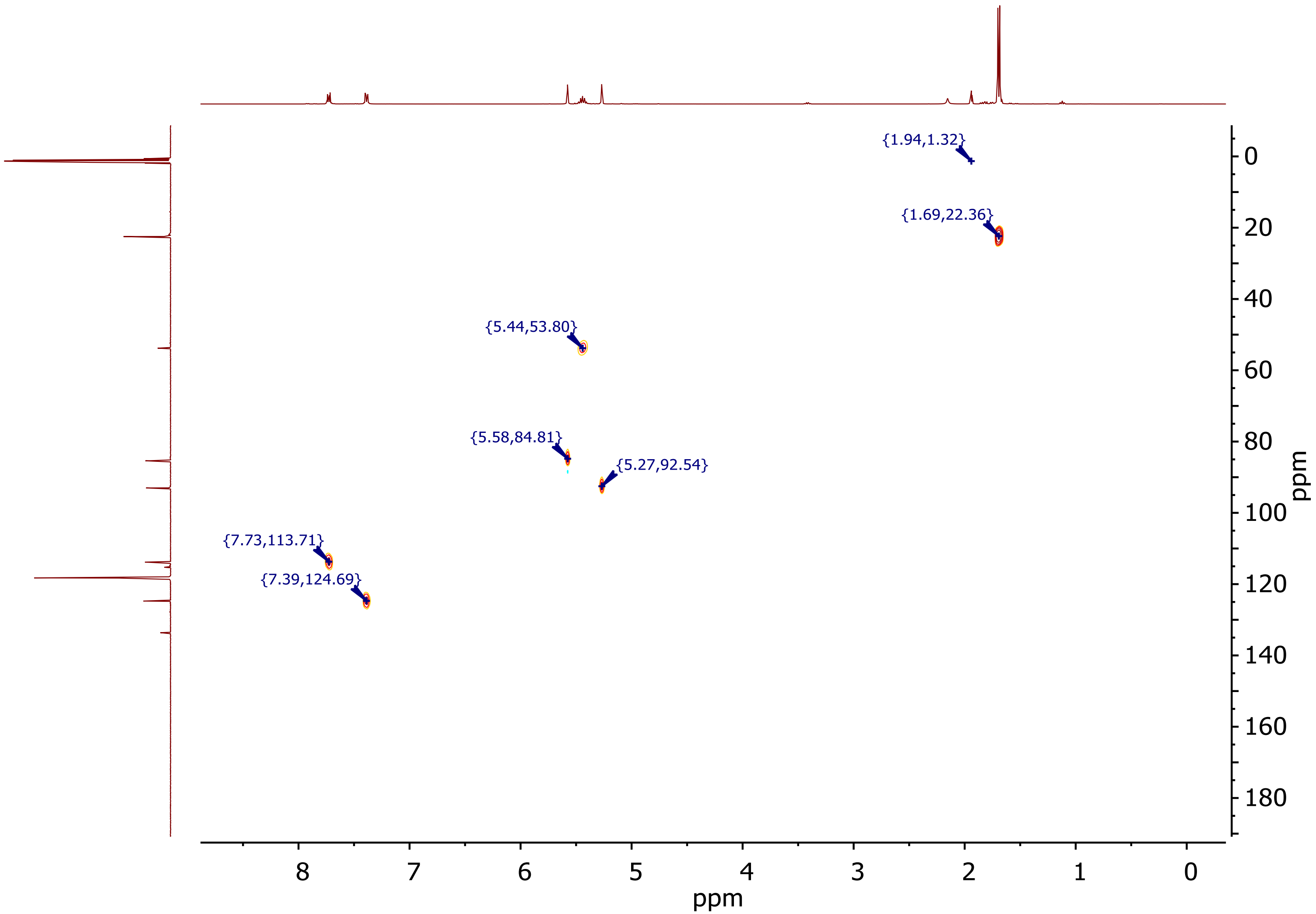

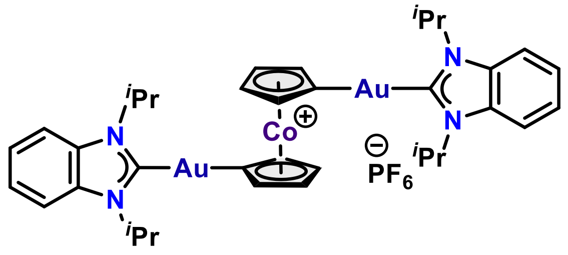

*Figure S 32: HSQC ^{1}H – ^{13}C (400 MHz, CD_3CN) **4b**.*

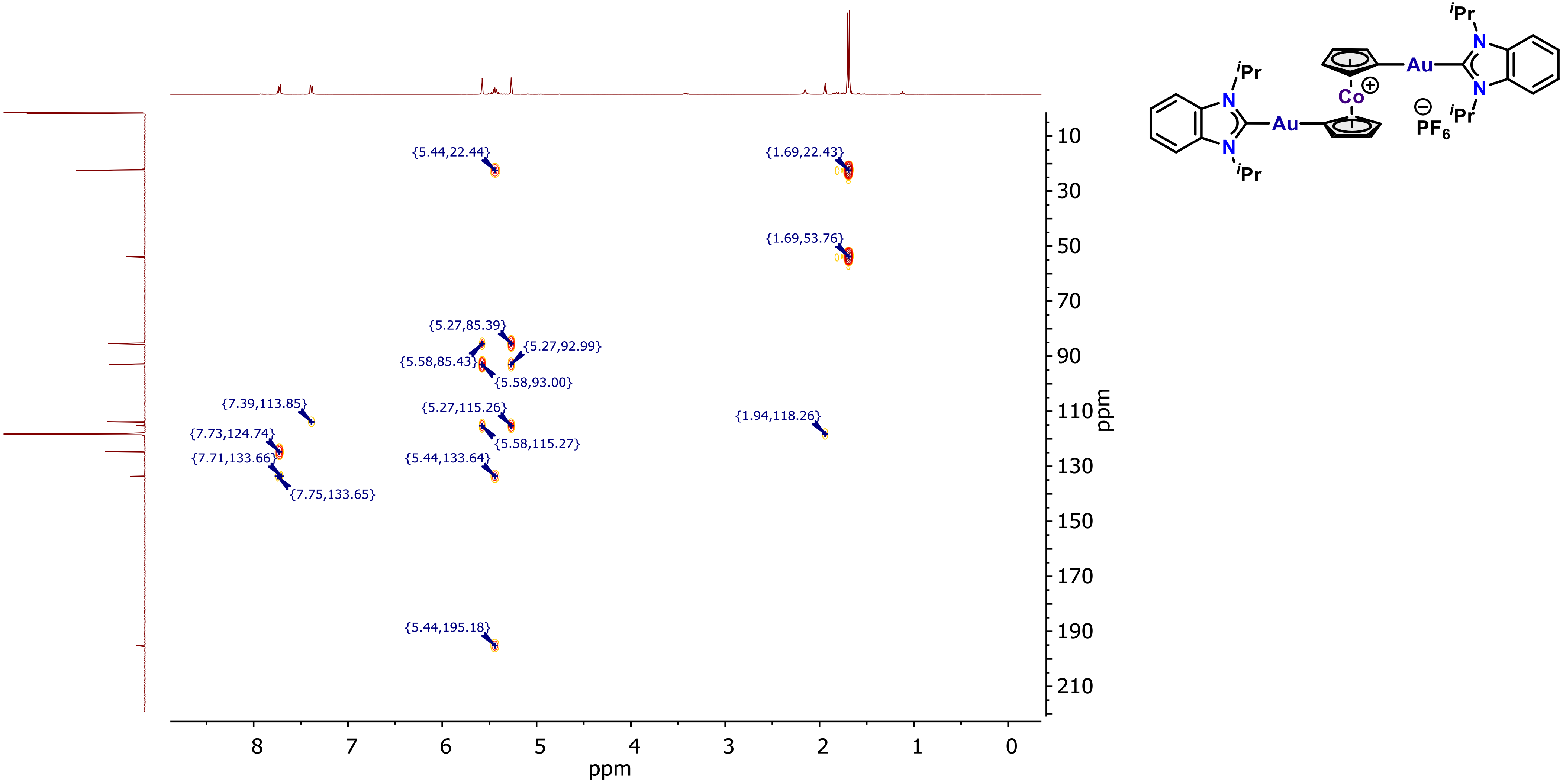

Figure S 33: HMBC ^{1}H – ^{13}C (400 MHz, CD_3CN) **4b**.

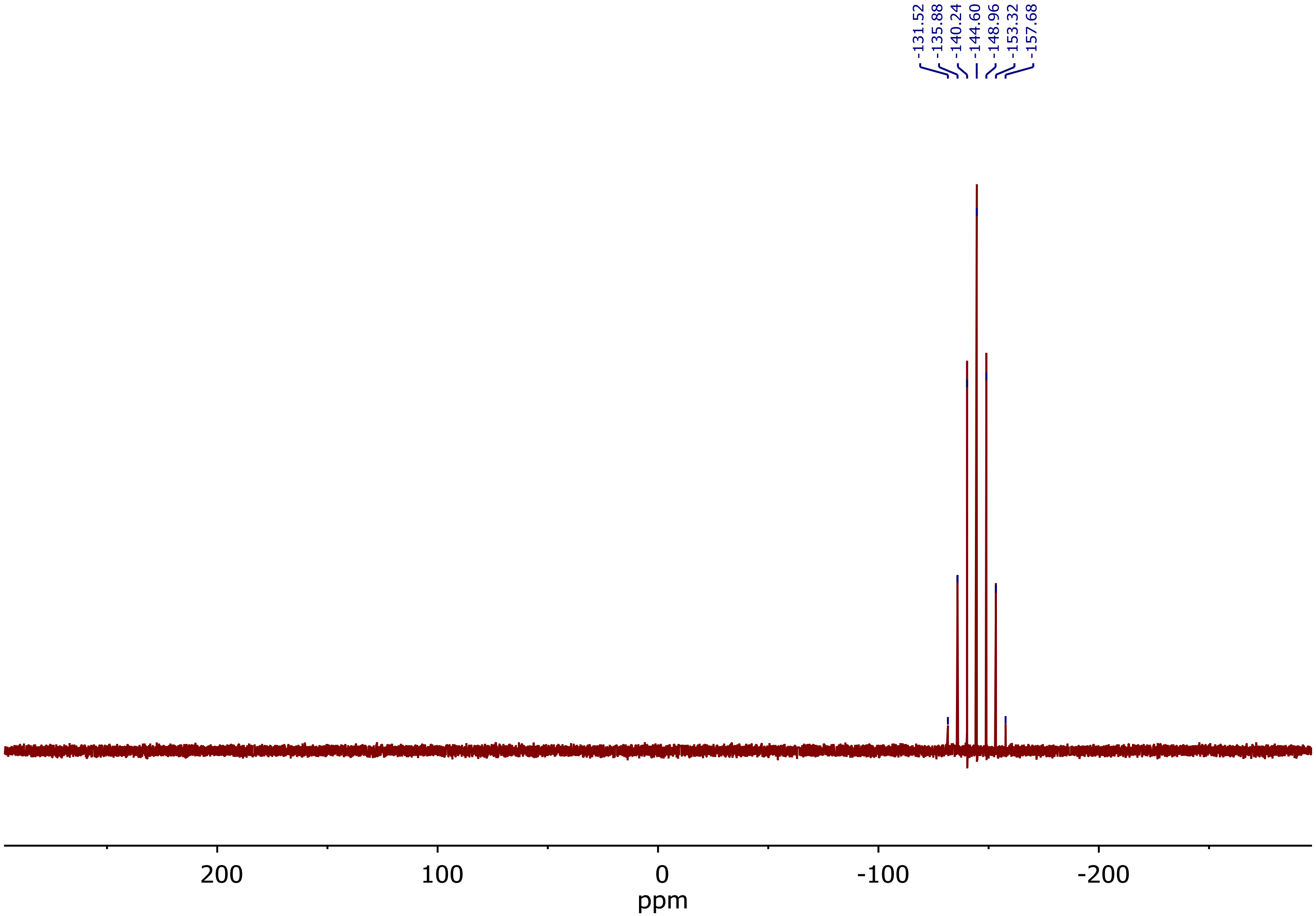

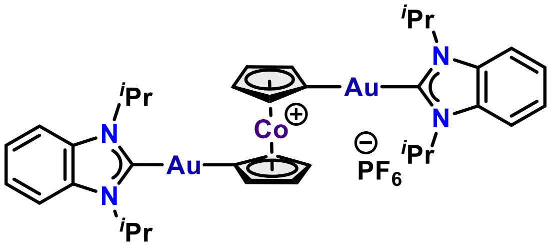

*Figure S 34: ^{31}P NMR (162 MHz, CD_3CN) **4b**.*

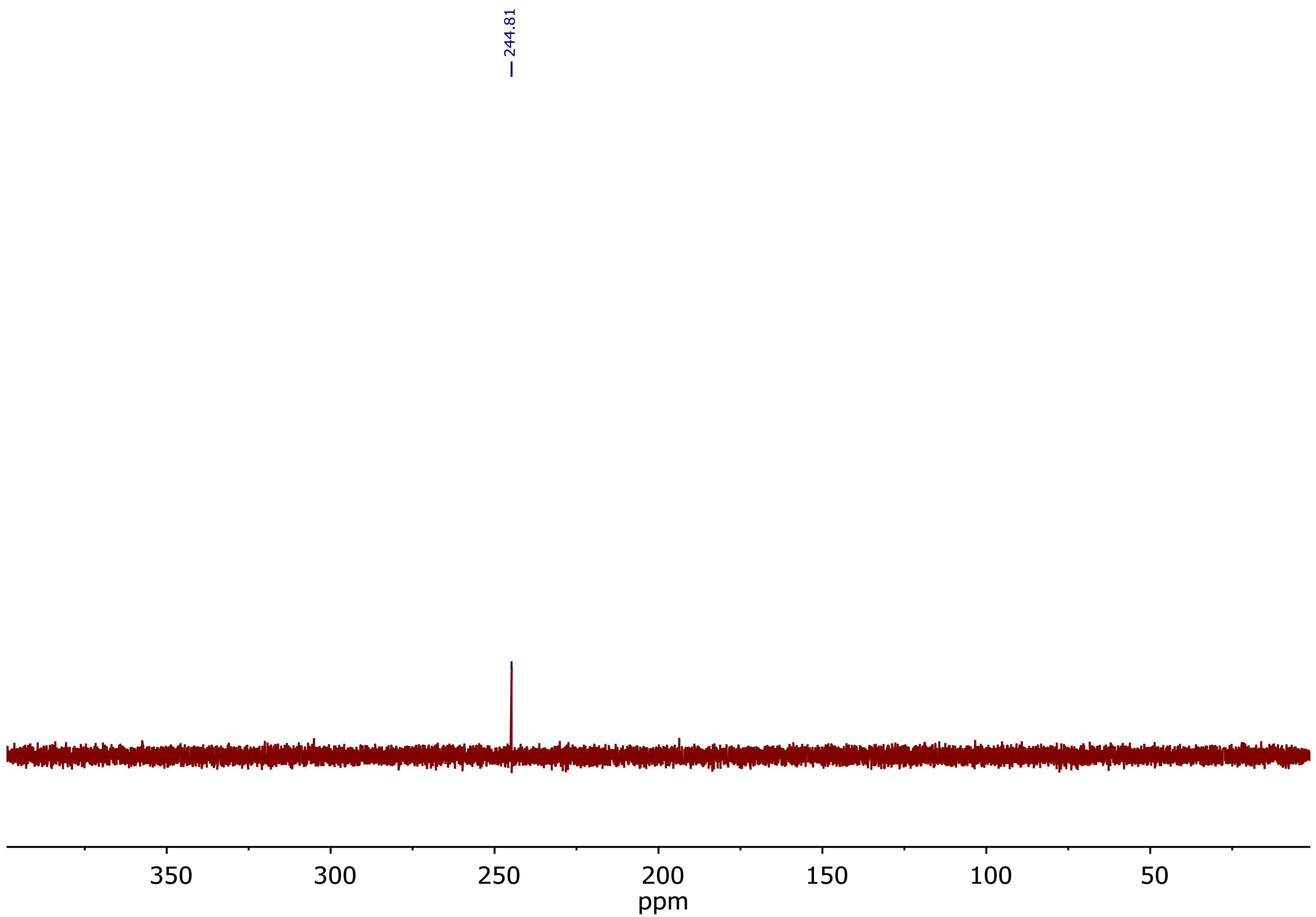

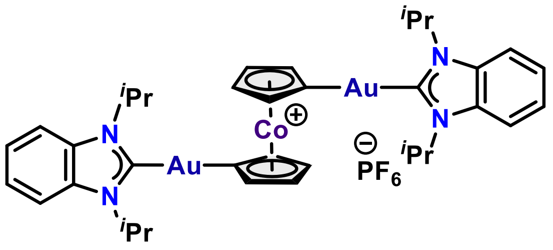

Figure S 35: ^{15}N NMR (41 MHz, CD_3CN) ***4b****.*

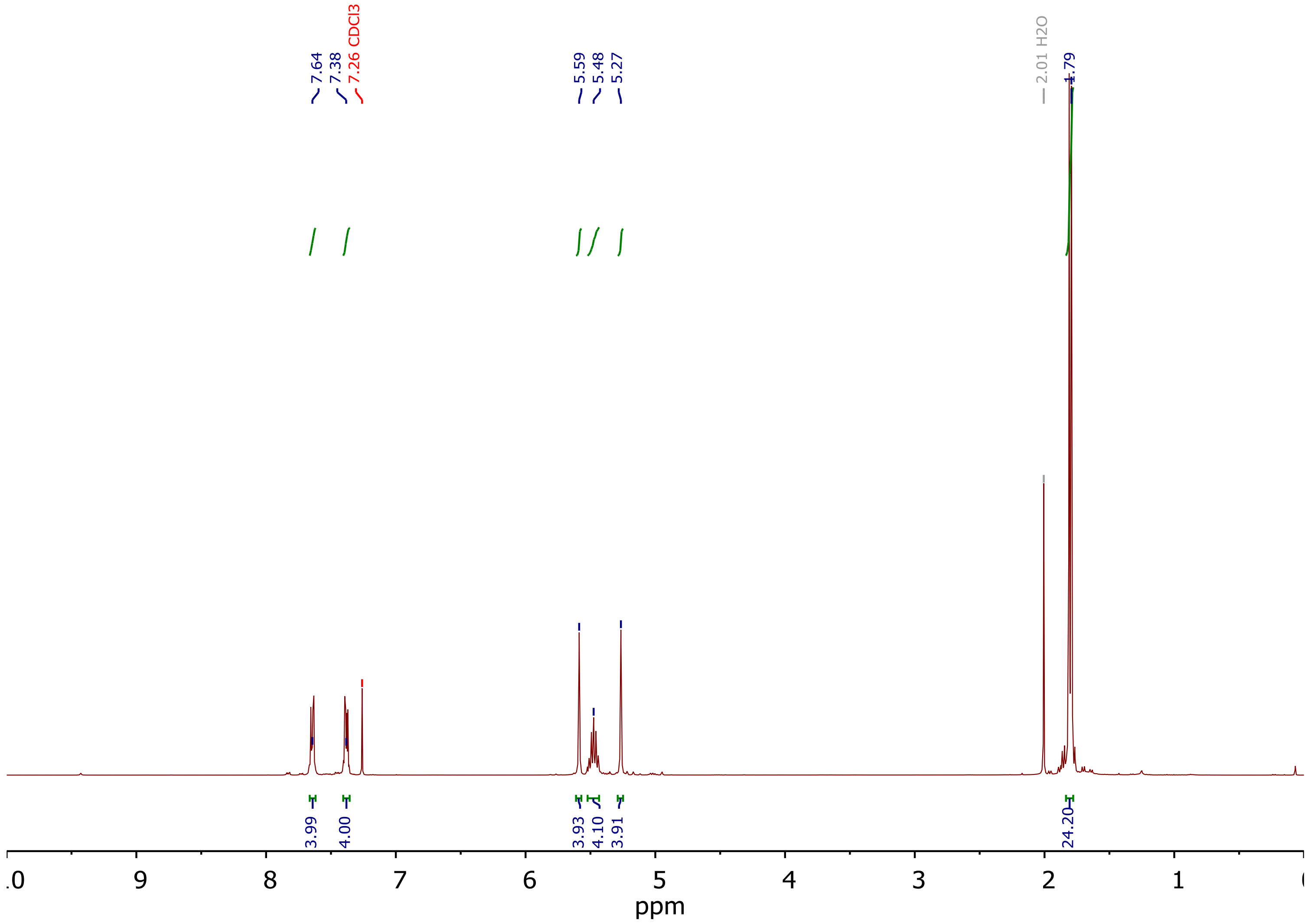

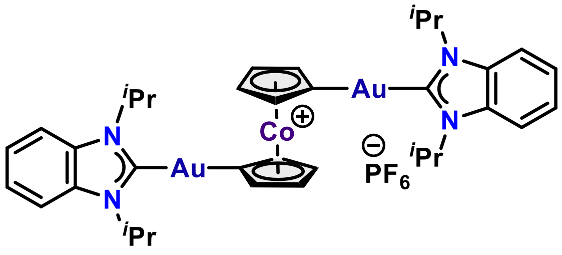

*Figure S 36: ^{1}H NMR (400 MHz, CDCl$_3$) **4b**.*

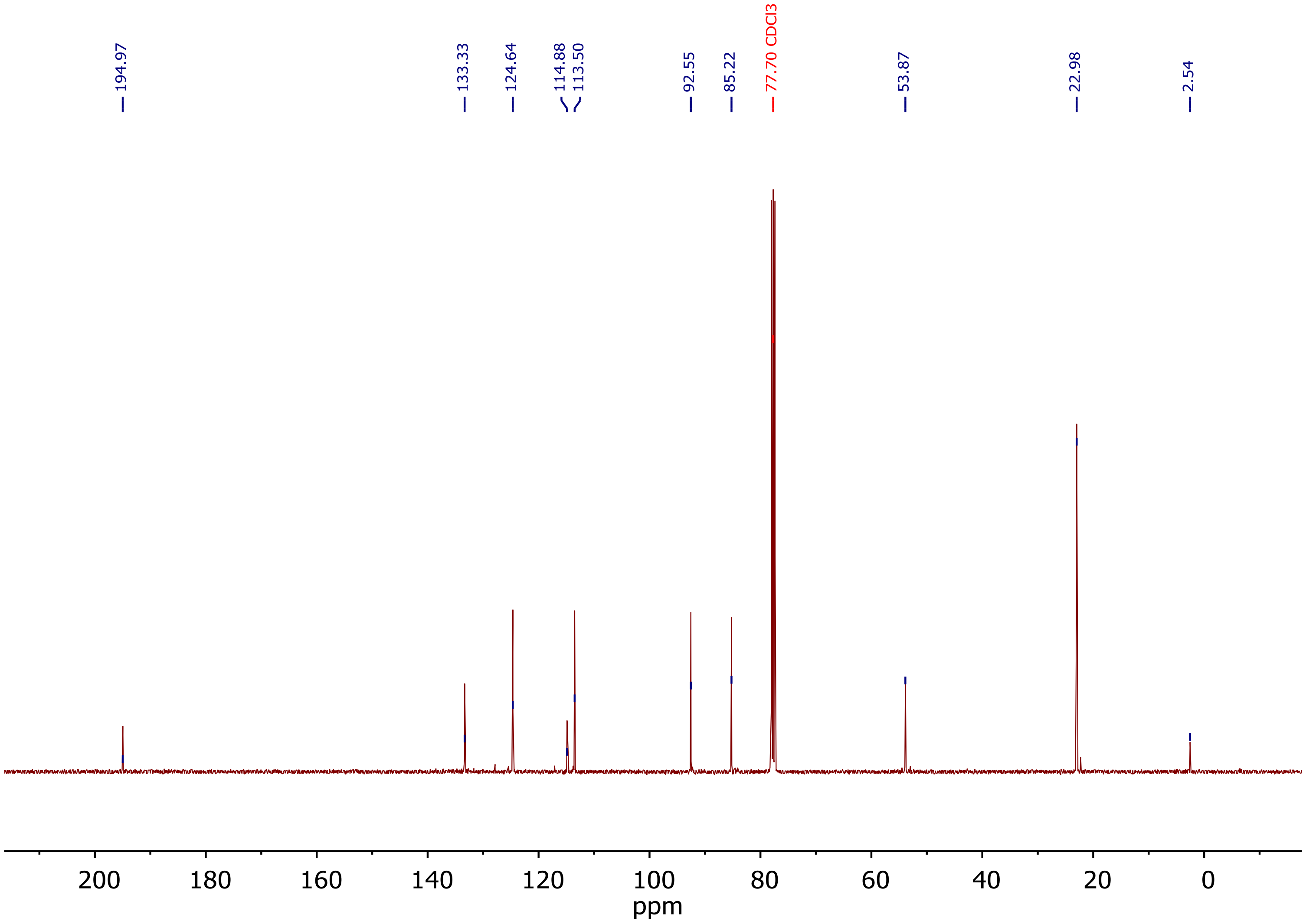

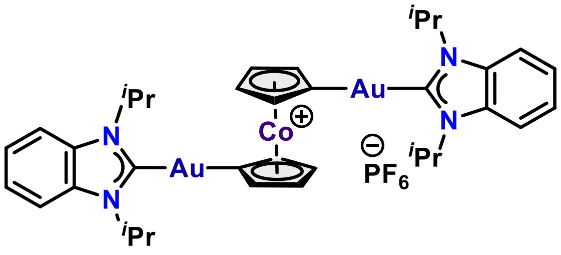

*Figure S 37: ^{13}C NMR (101 MHz, $CDCl_3$) **4b**.*

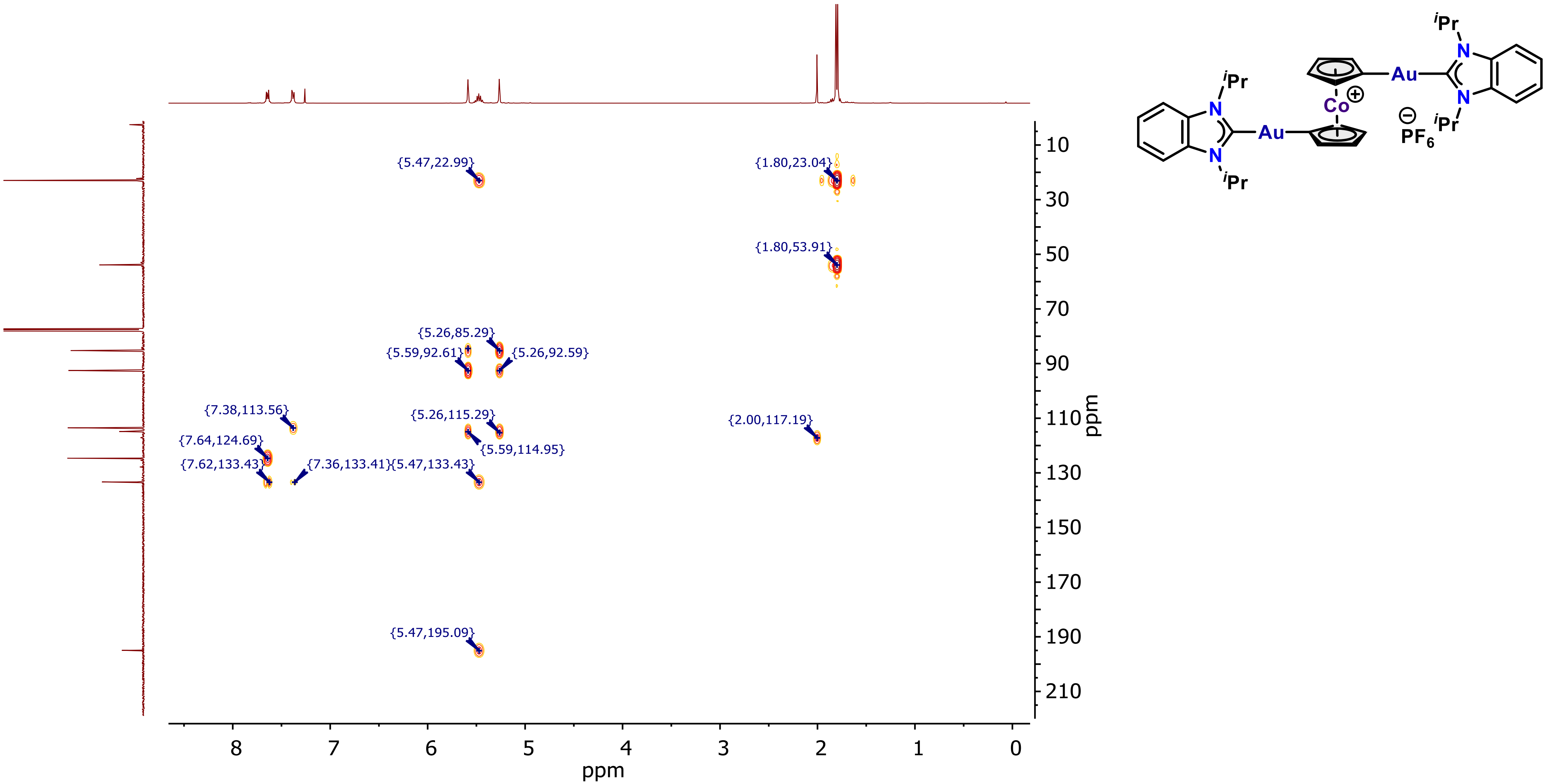

Figure S 38: HMBC ^{1}H – ^{13}C (400 MHz, $CDCl_3$) ***4b****.*

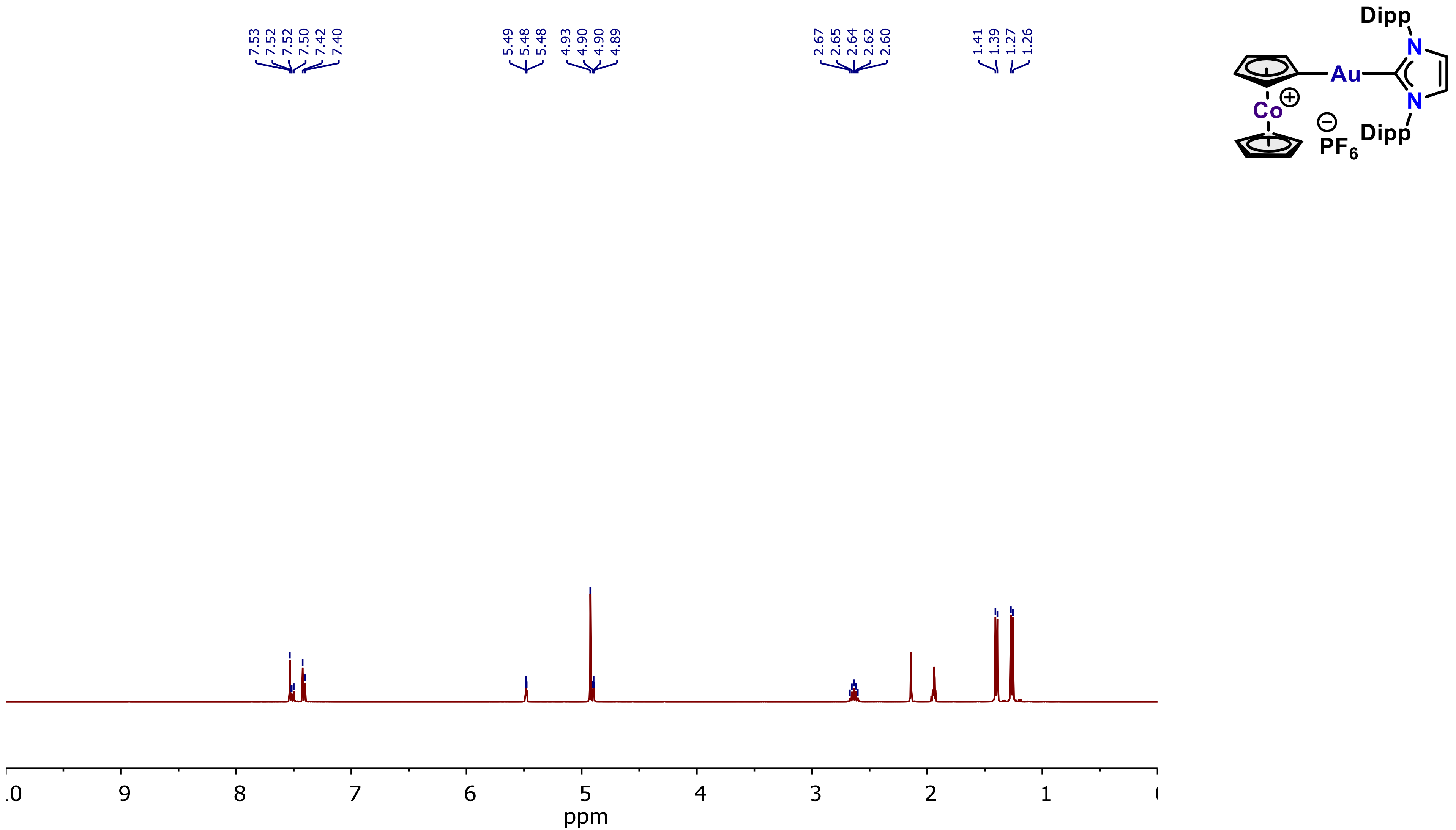

Figure S 39: ^{1}H NMR (400 MHz, CD_3CN) ***5a****.*

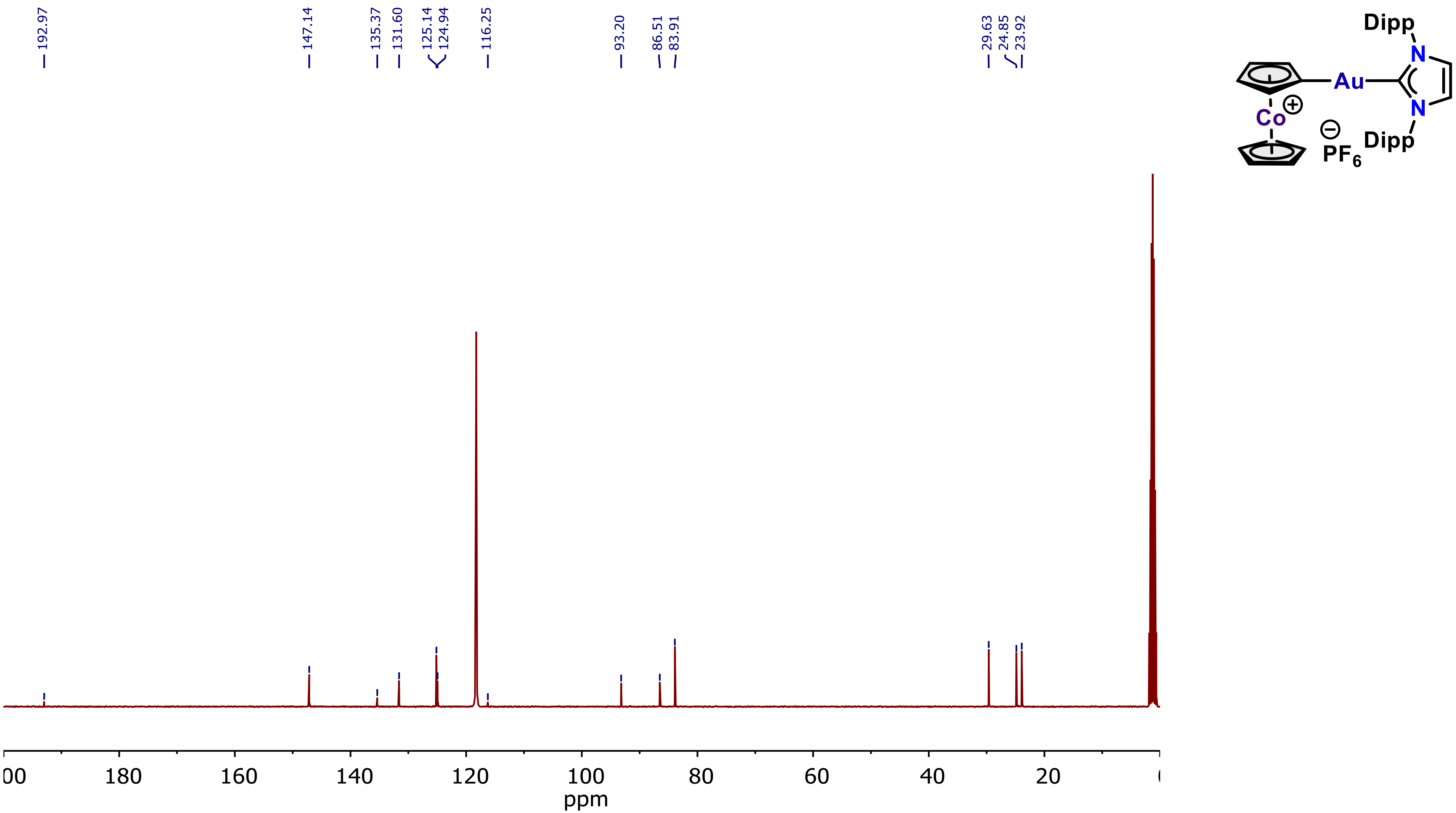

Figure S 40: ^{13}C NMR (101 MHz, CD_3CN) **5a**.

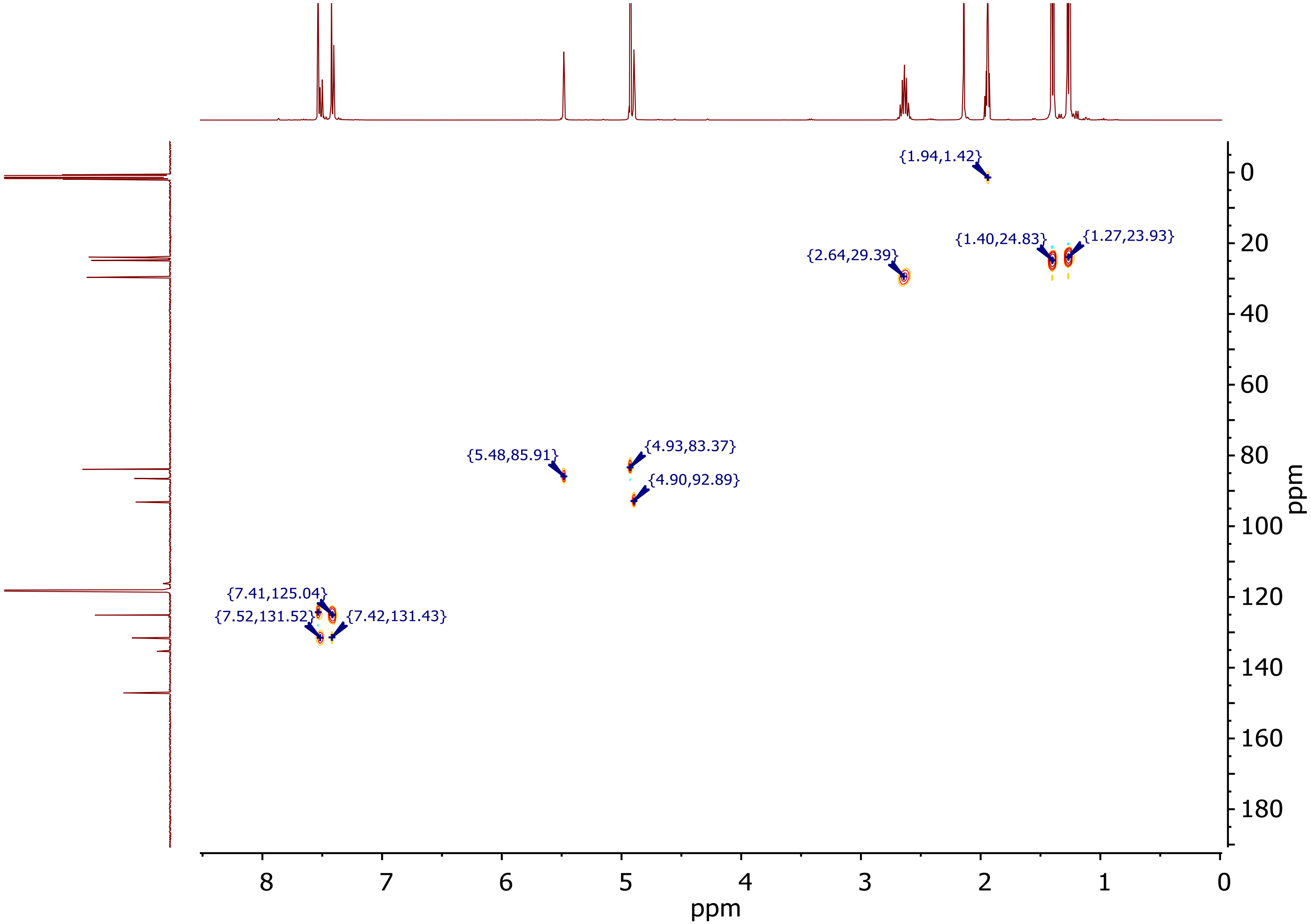

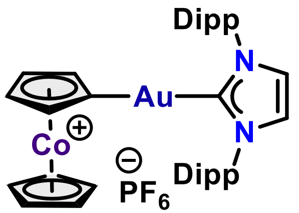

Figure S 41: HSQC ^{1}H – ^{13}C (400 MHz, CD_3CN) ***5a***.

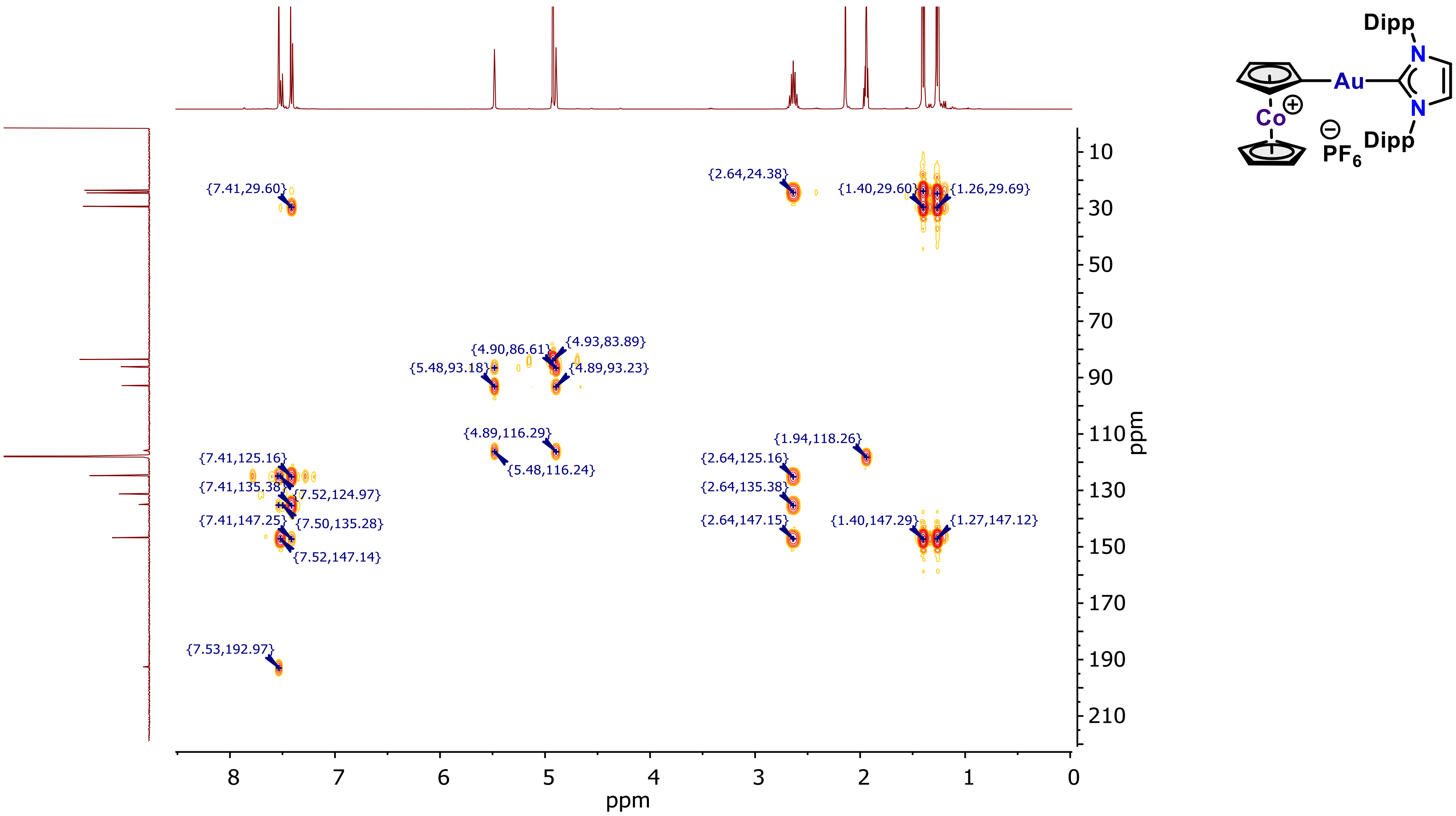

Figure S 42: HMBC ^1H – ^{13}C (400 MHz, CD_3CN) ***5a****.*

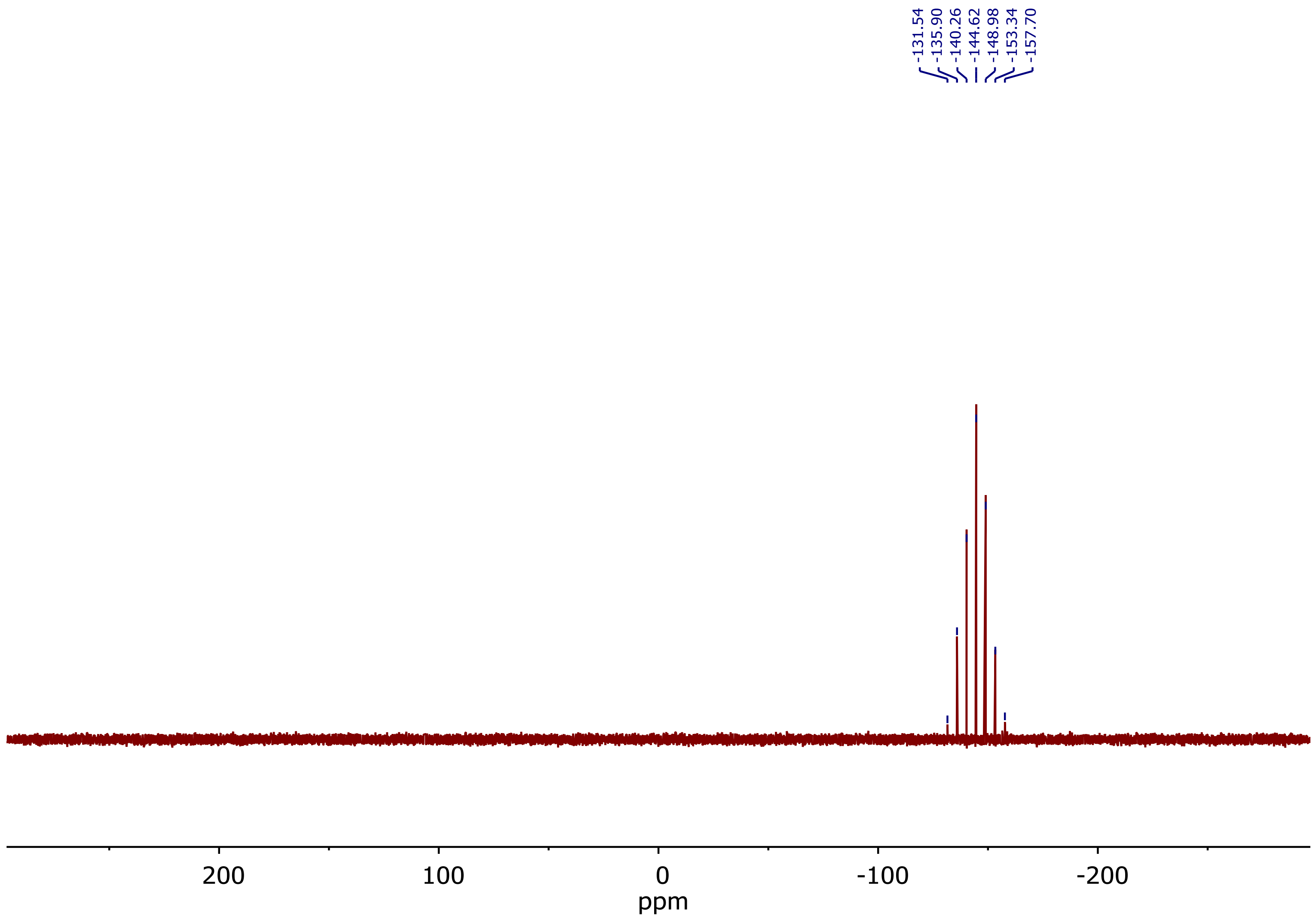

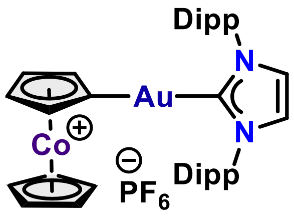

*Figure S 43: ^{31}P NMR (162 MHz, CD_3CN) **5a**.*

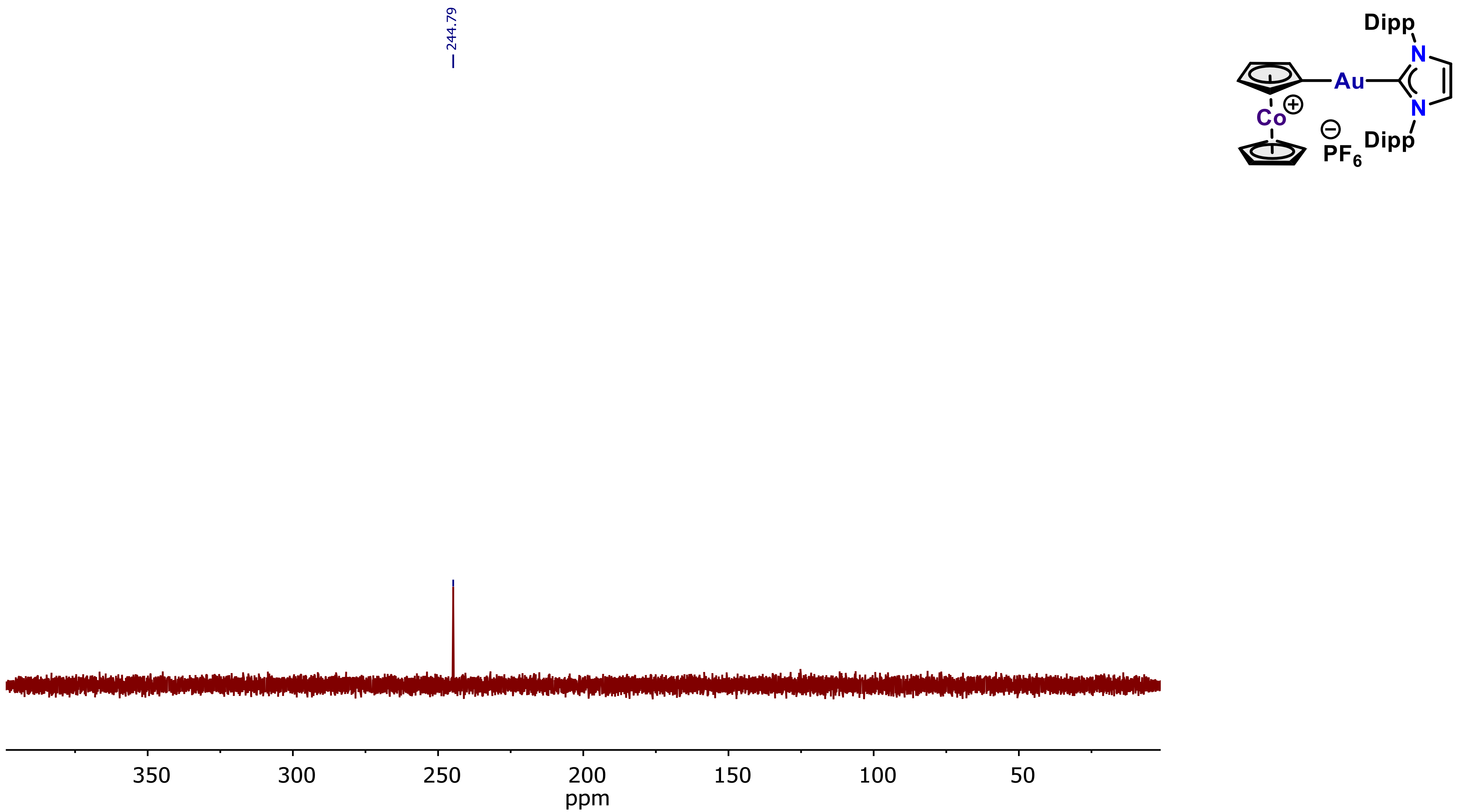

*Figure S 44: ^{15}N NMR (41 MHz, CD_3CN) **5a**.*

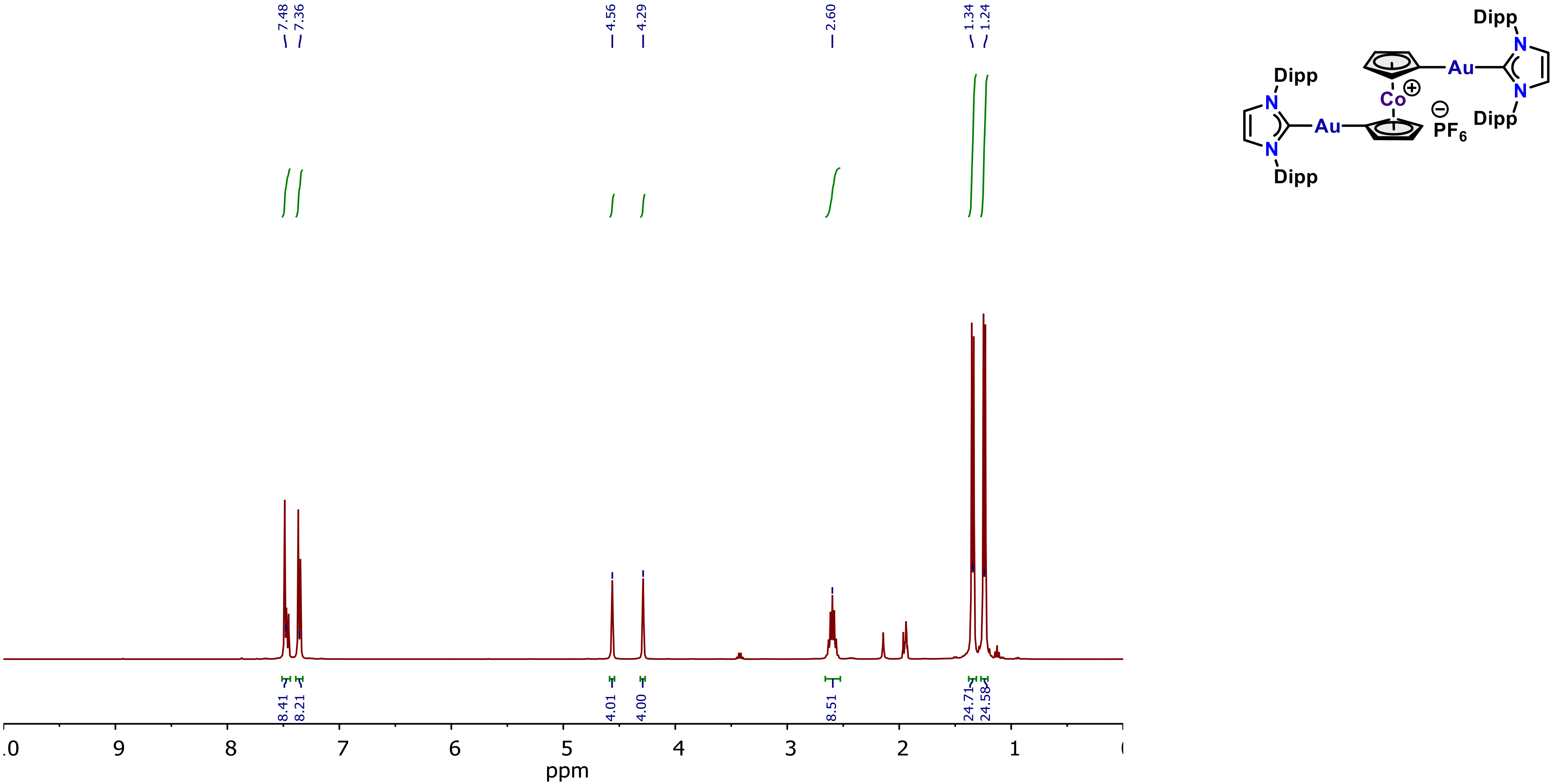

Figure S 45: ¹H NMR (400 MHz, CD_3CN) ***5b****.*

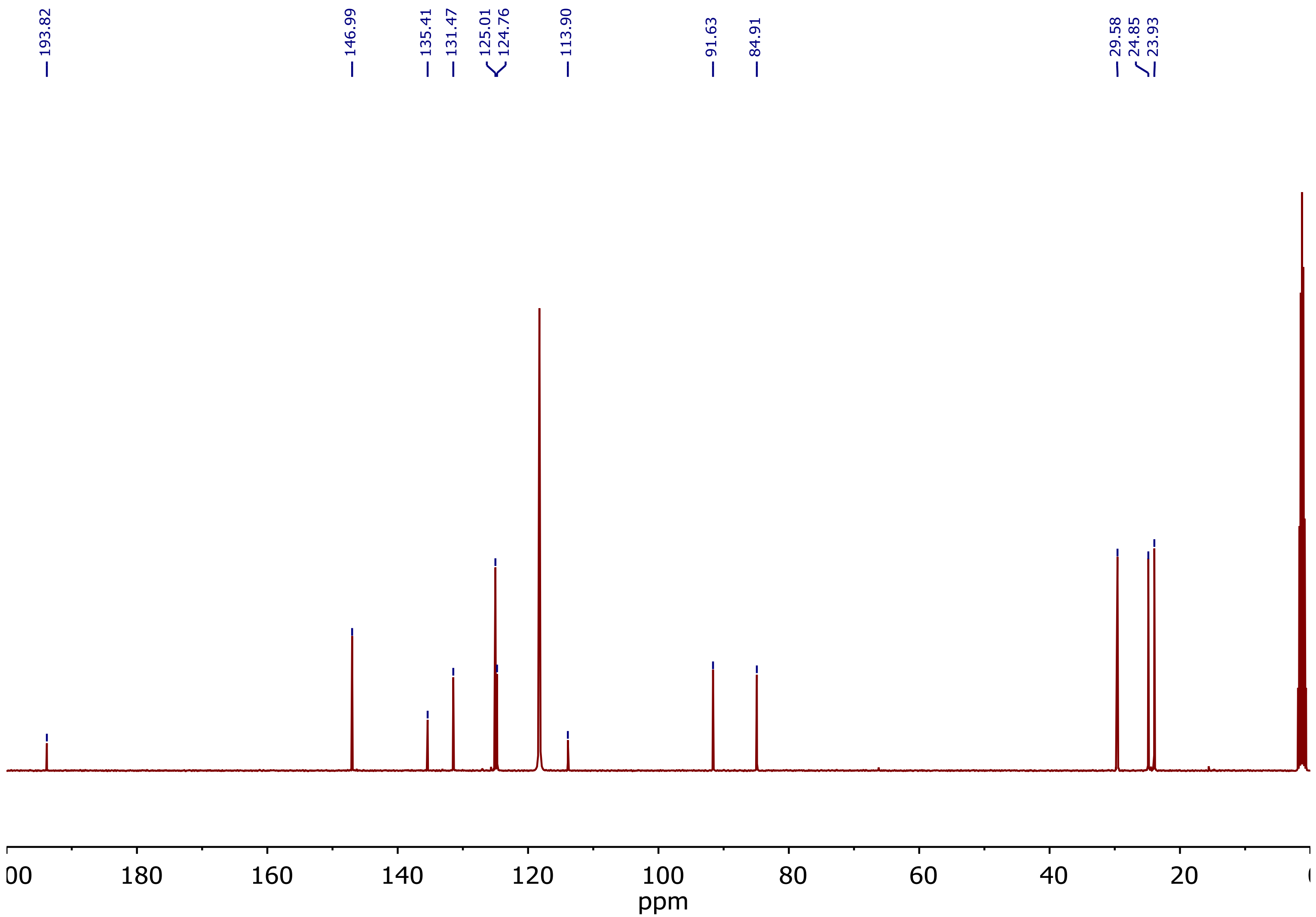

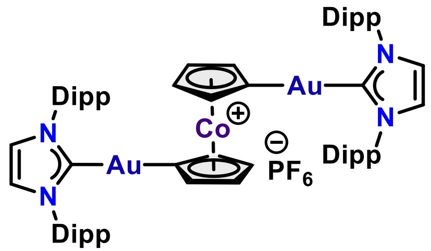

Figure S 46: ^{13}C NMR (101 MHz, CD_3CN) ***5b****.*

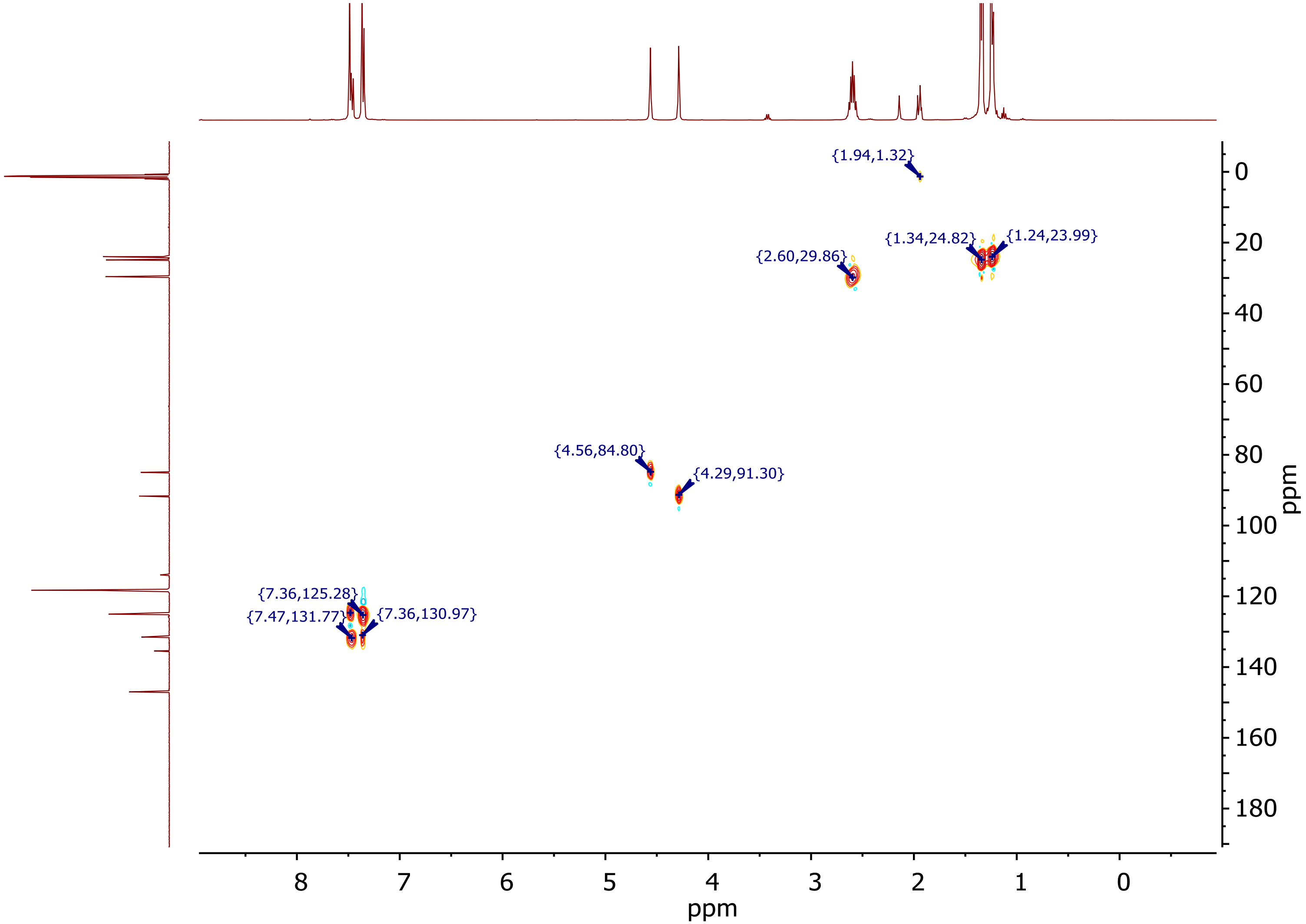

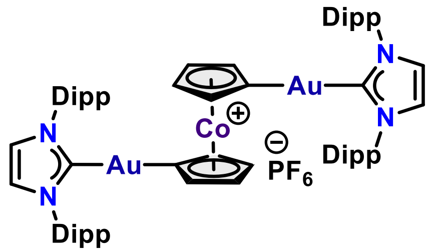

Figure S 47: HSQC ^{1}H – ^{13}C (400 MHz, CD_3CN) **5b**.

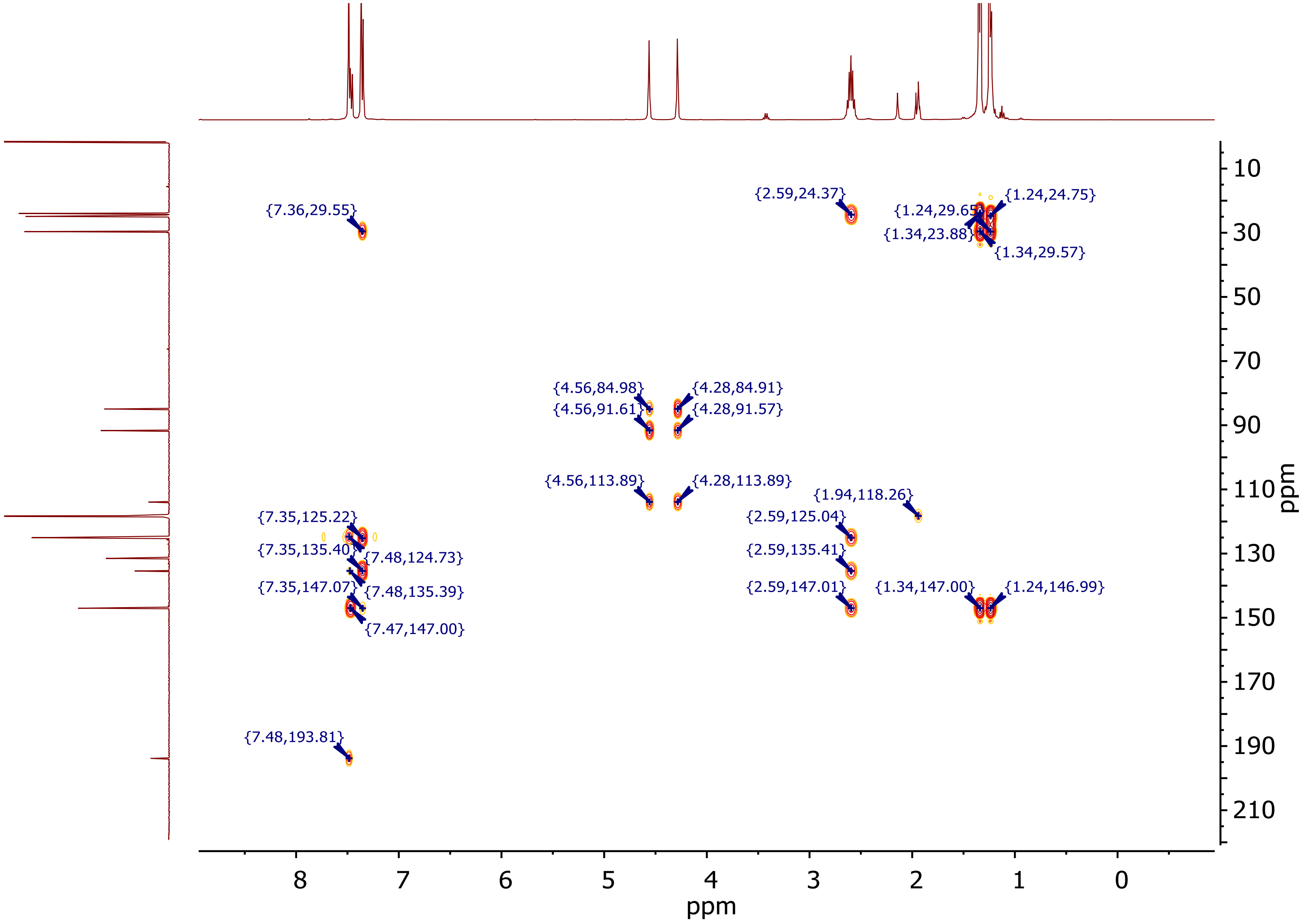

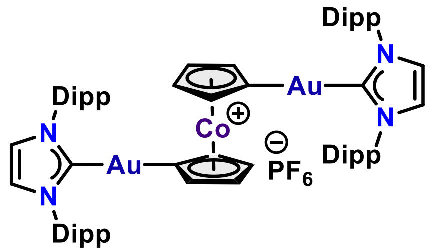

Figure S 48: HMBC ^{1}H – ^{13}C (400 MHz, CD_3CN) ***5b****.*

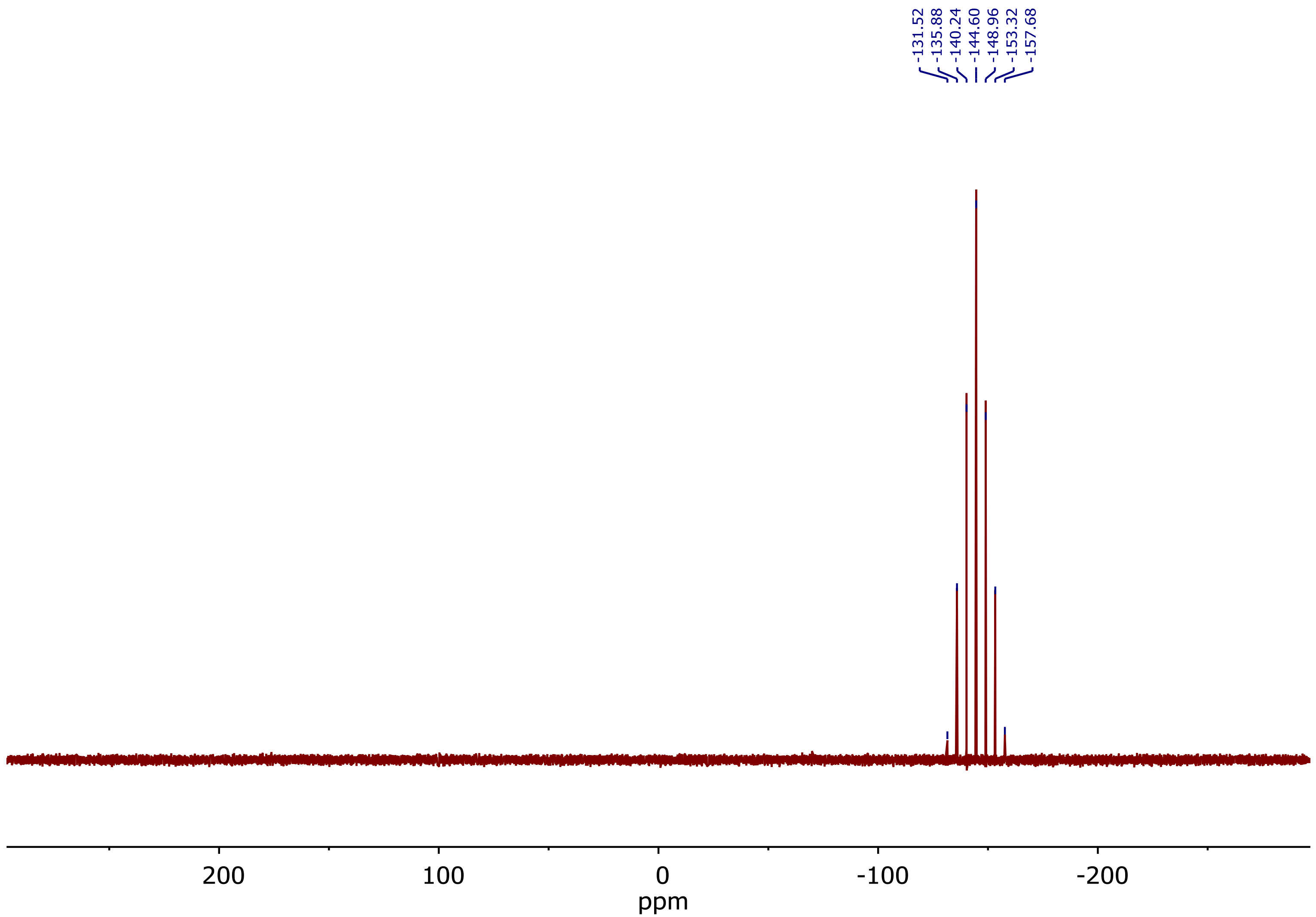

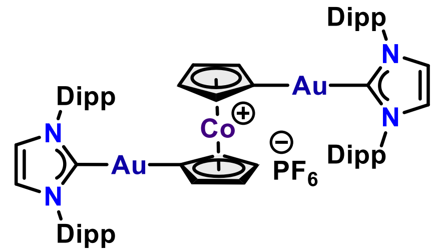

*Figure S 49: ^{31}P NMR (162 MHz, CD_3CN) **5b**.*

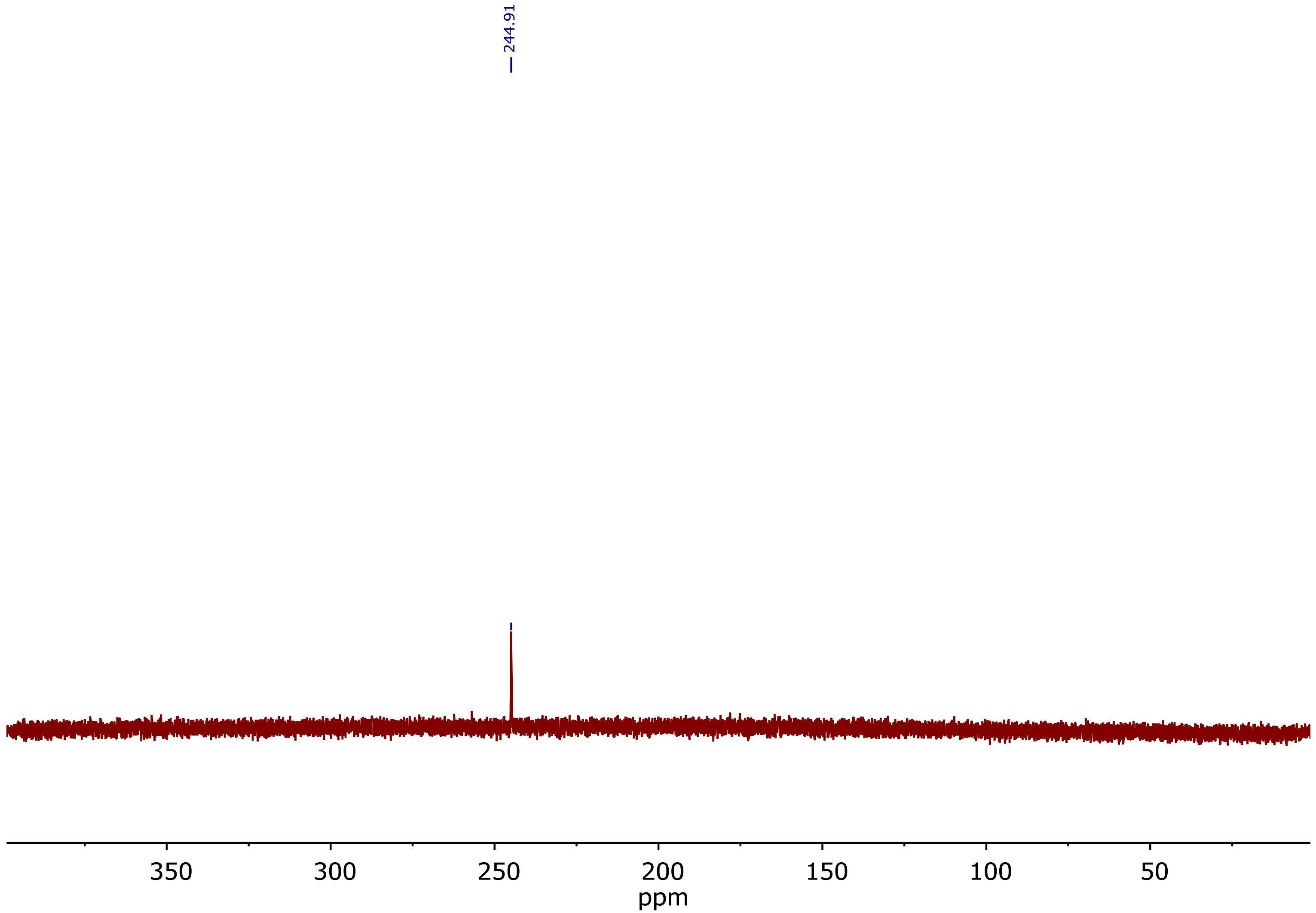

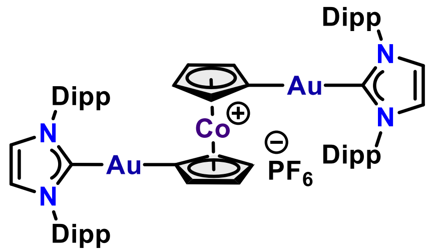

Figure S 50: ^{15}N NMR (41 MHz, CD_3CN) ***5b****.*

IR Spectra

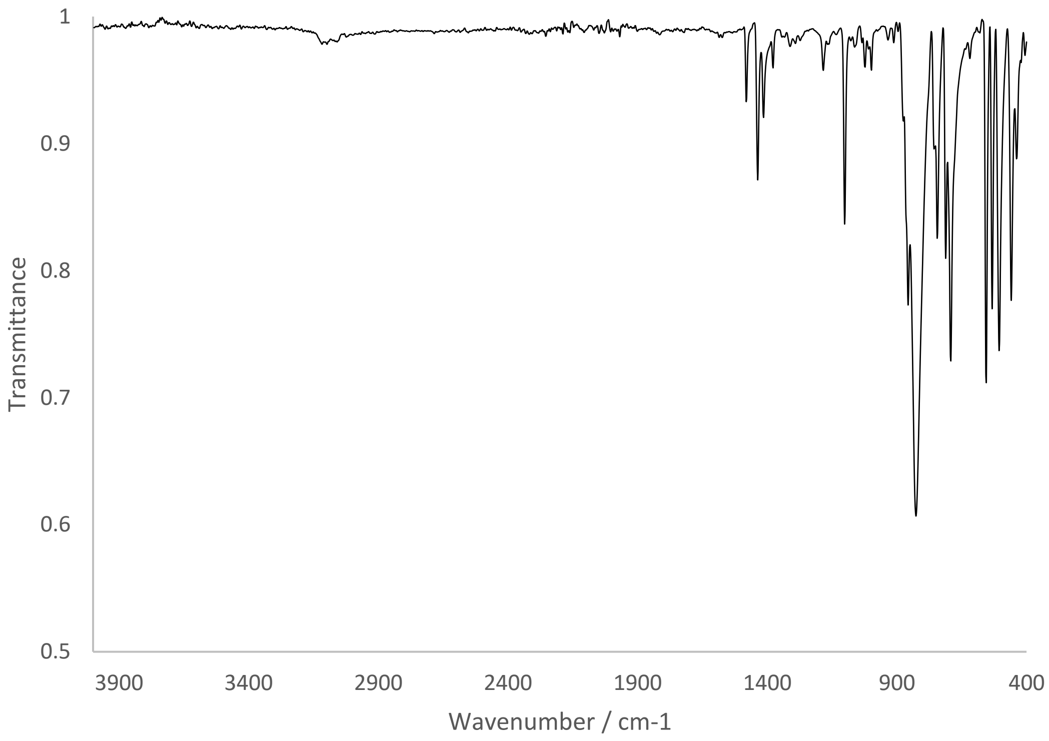

Figure S 51: IR (ATR) ***2a****.*

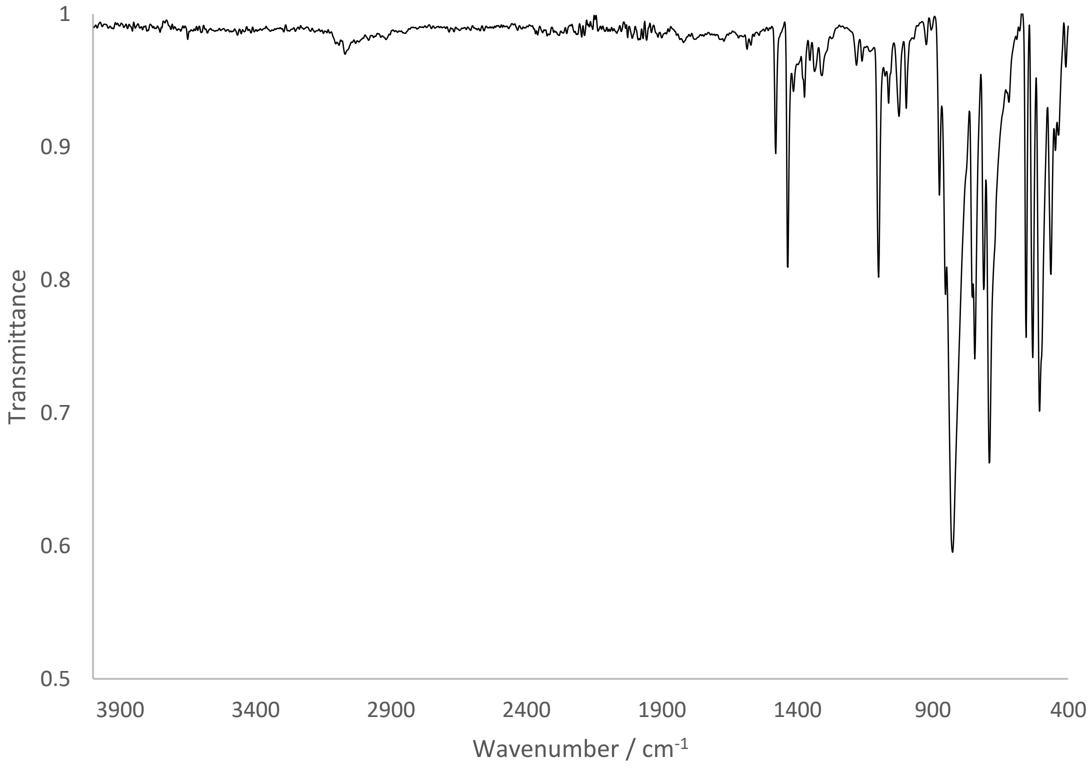

Figure S 52: IR (ATR) ***2b****.*

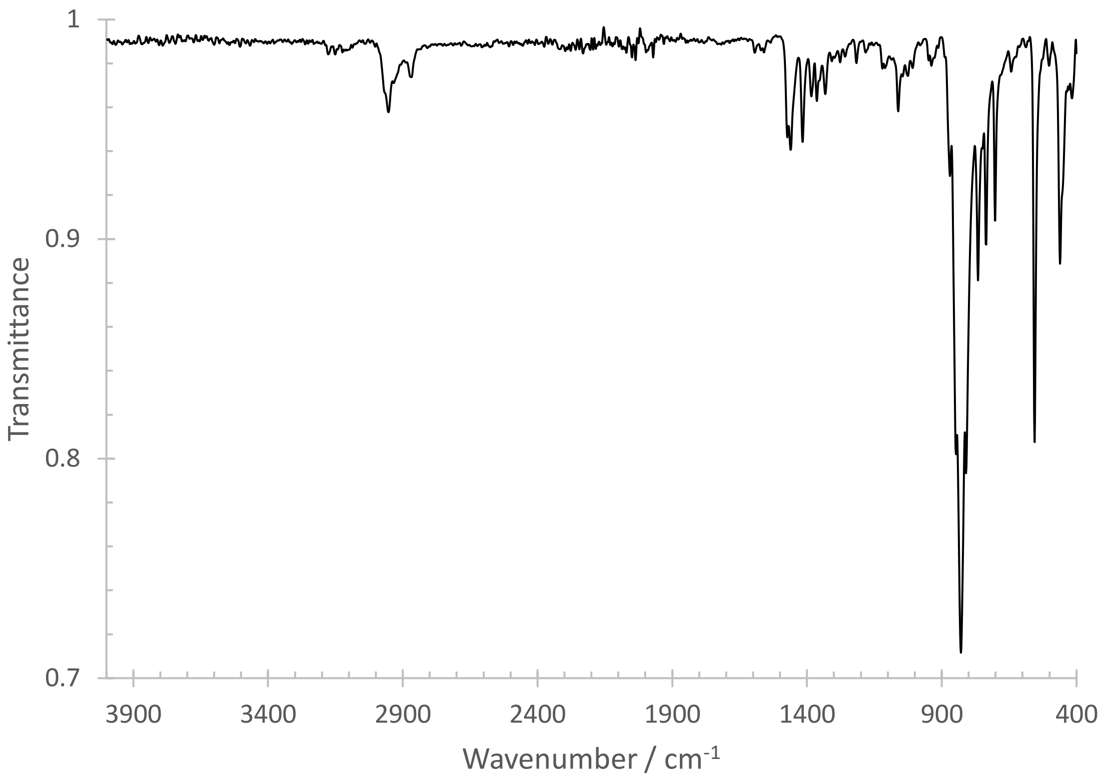

Figure S 53: IR (ATR) ***3a****.*

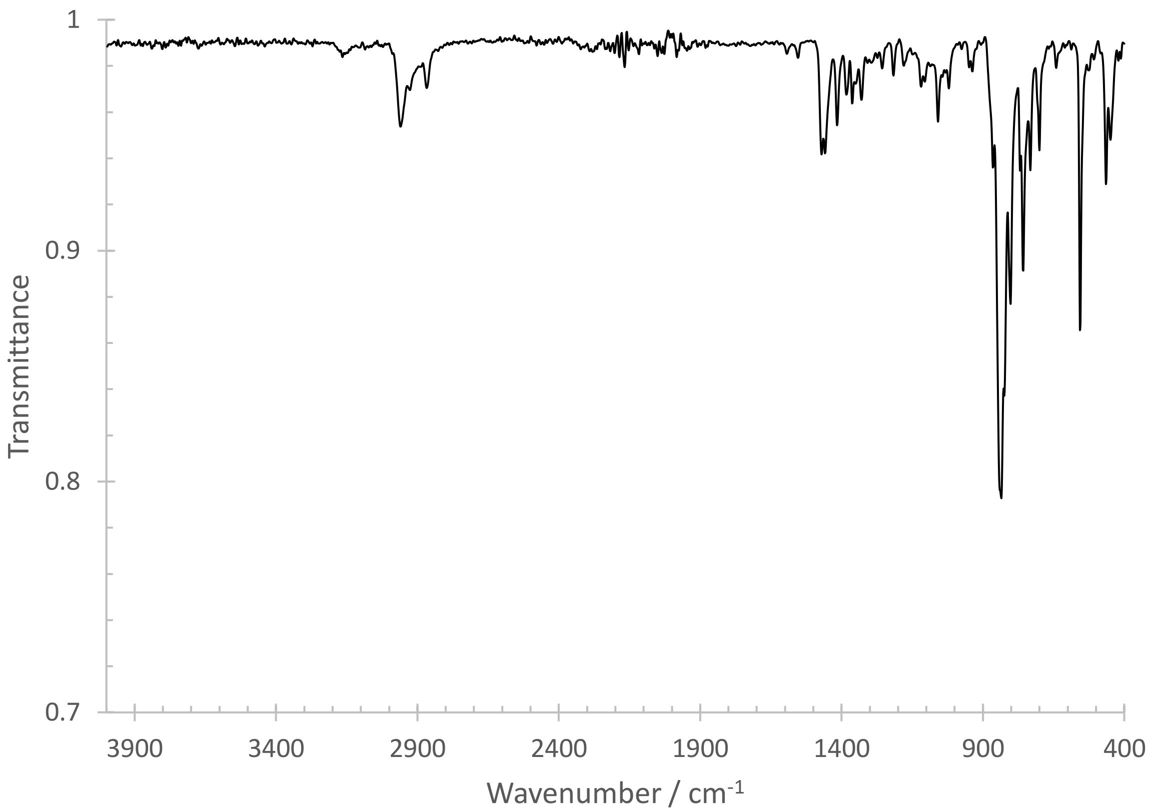

Figure S 54: IR (ATR) ***3b****.*

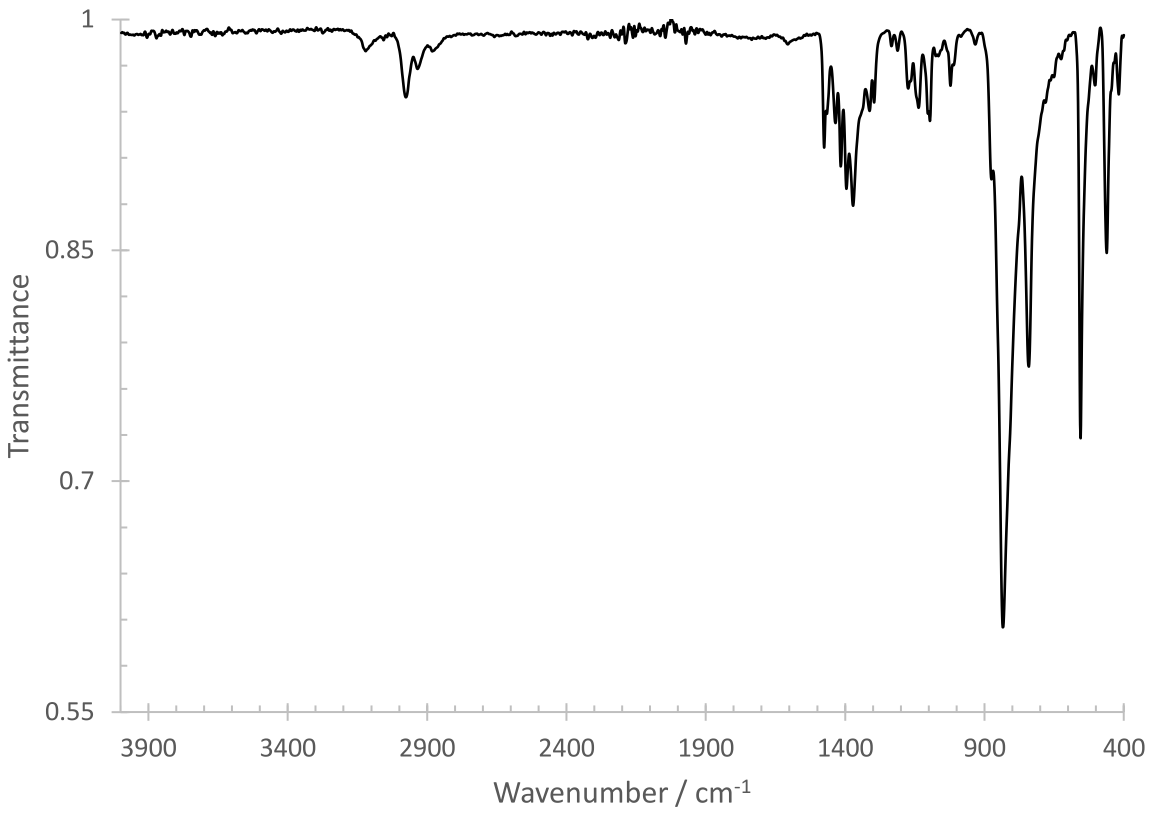

Figure S 55: IR (ATR) ***4a****.*

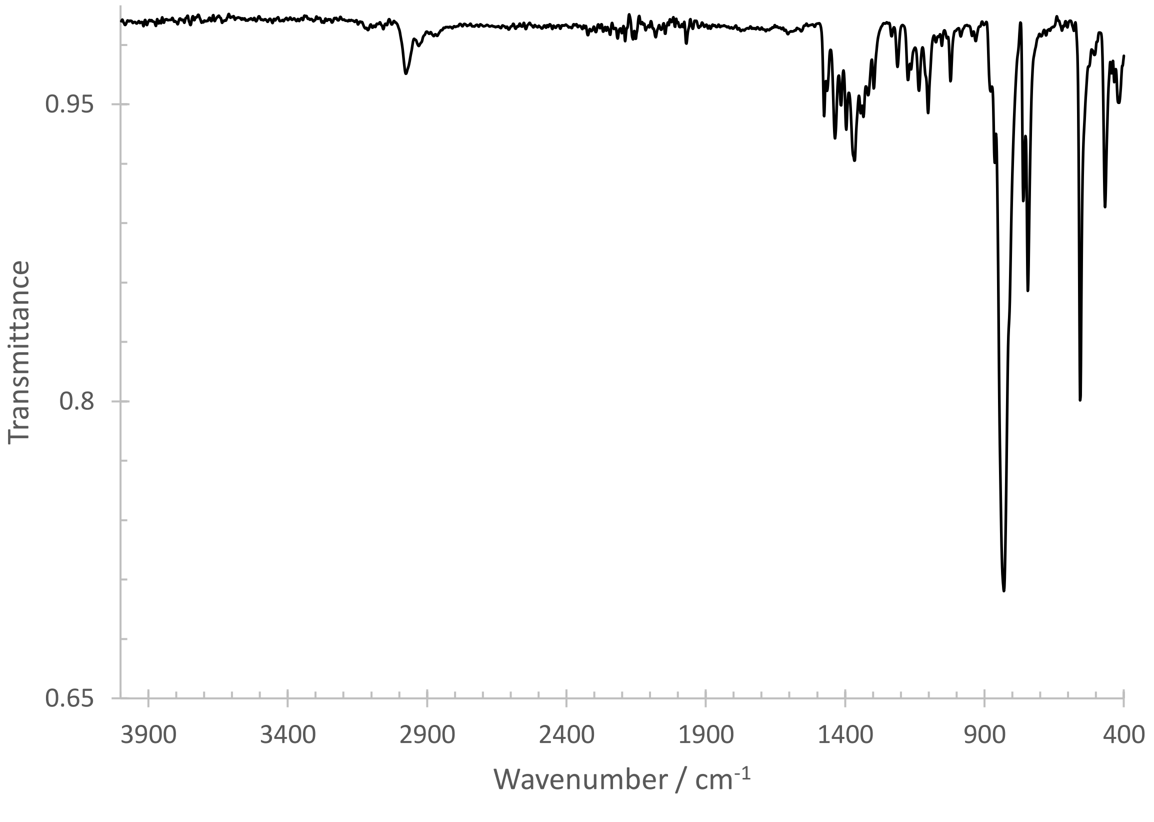

Figure S 56: IR (ATR) ***4b****.*

ESI – MS

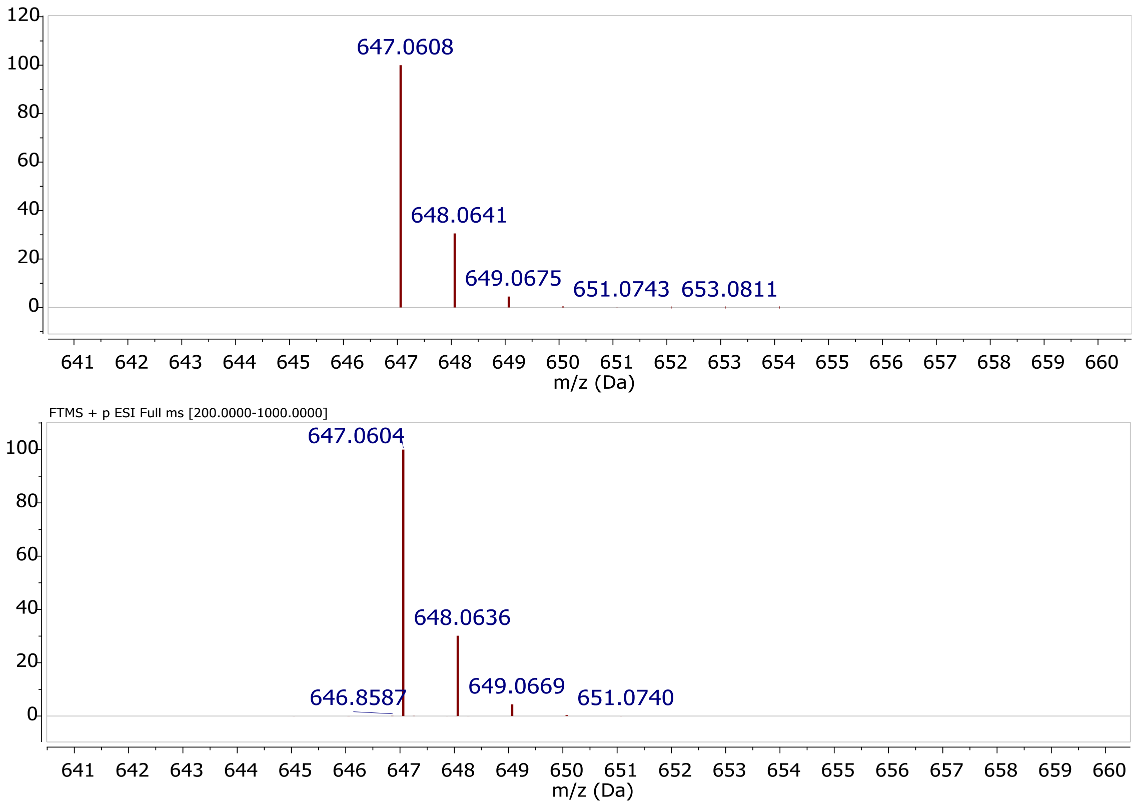

Figure S 57: MS (ESI+) ***2a.*** *Top: simulated.*

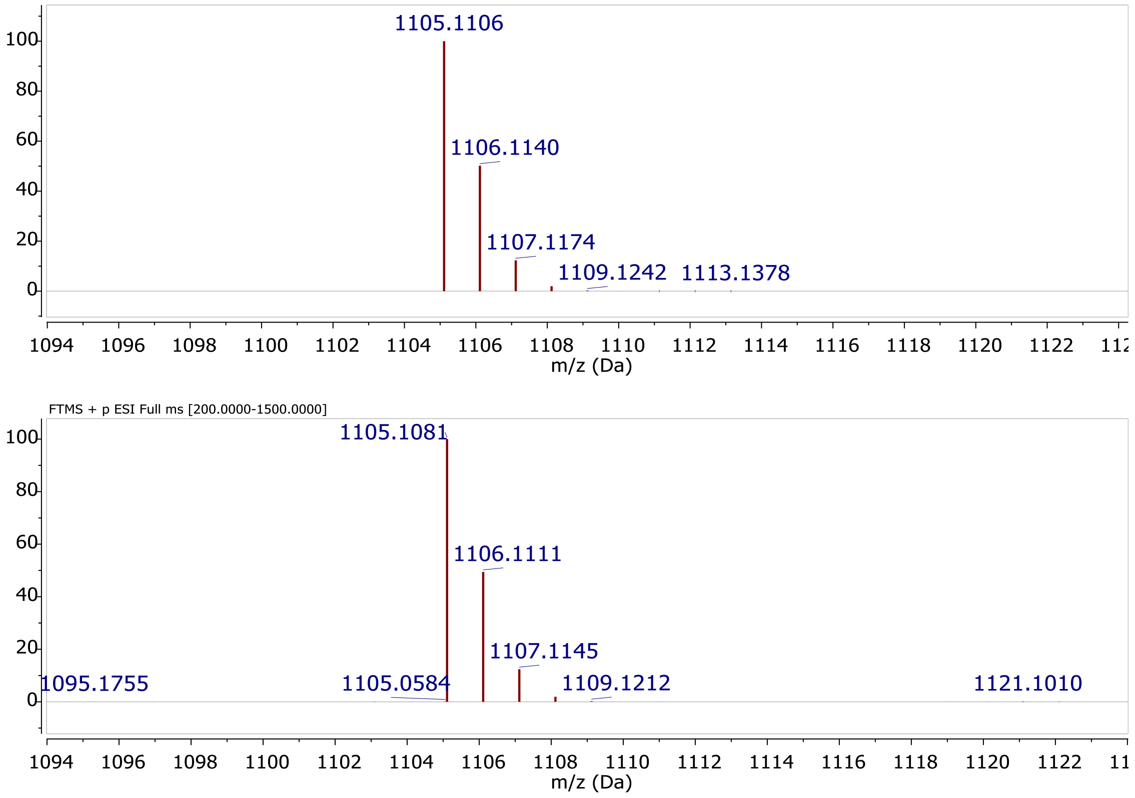

Figure S 58: MS (ESI+) ***2b.*** *Top: simulated.*

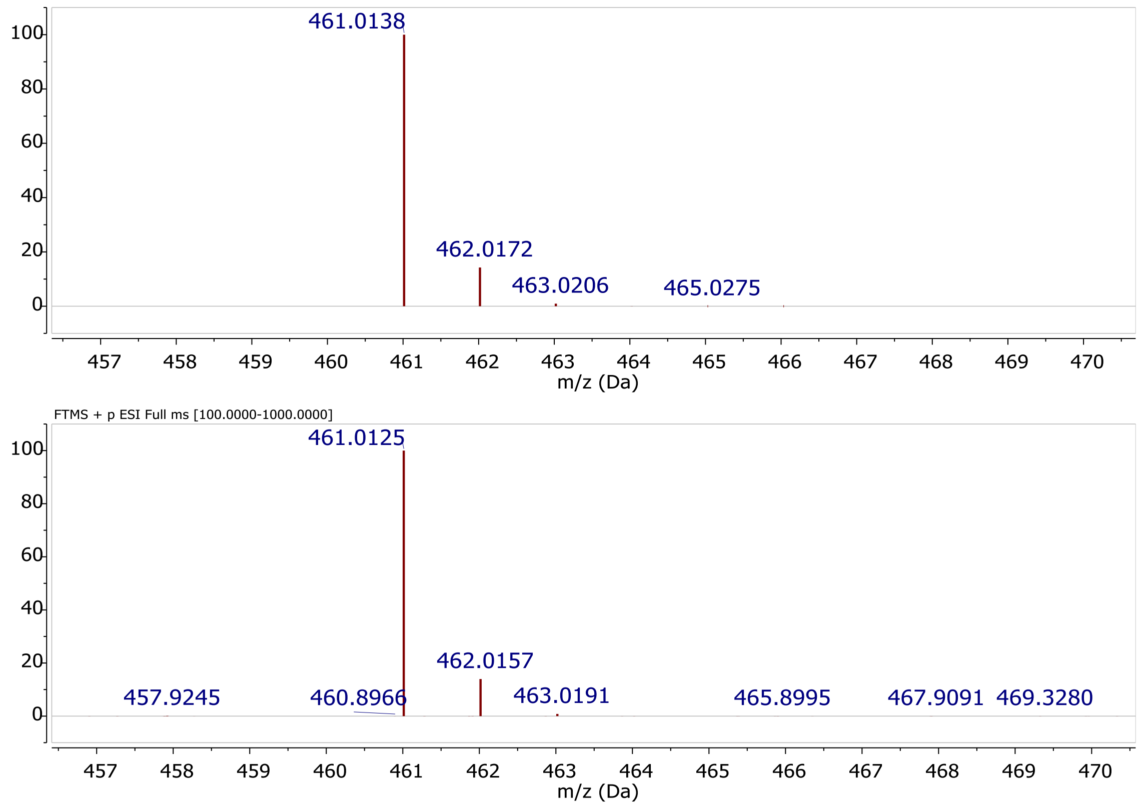

Figure S 59: MS (ESI+) ***3a.*** *Top: simulated.*

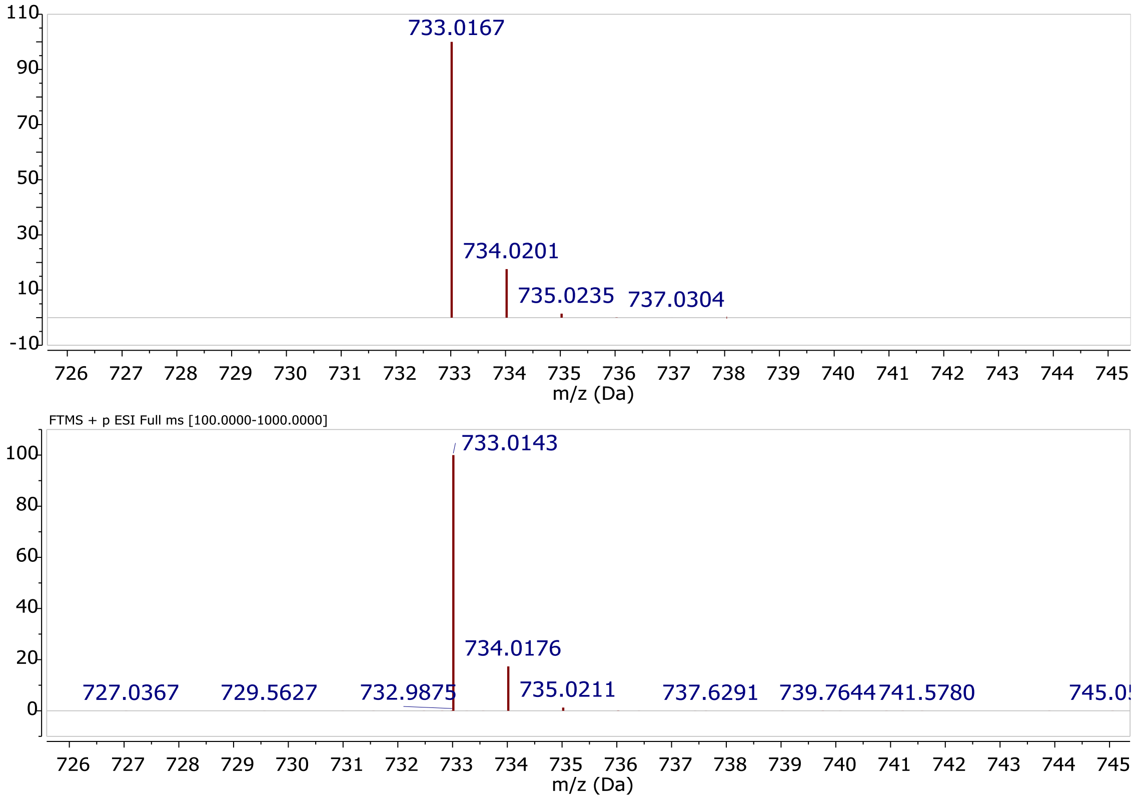

Figure S 60: MS (ESI+) ***3b.*** *Top: simulated.*

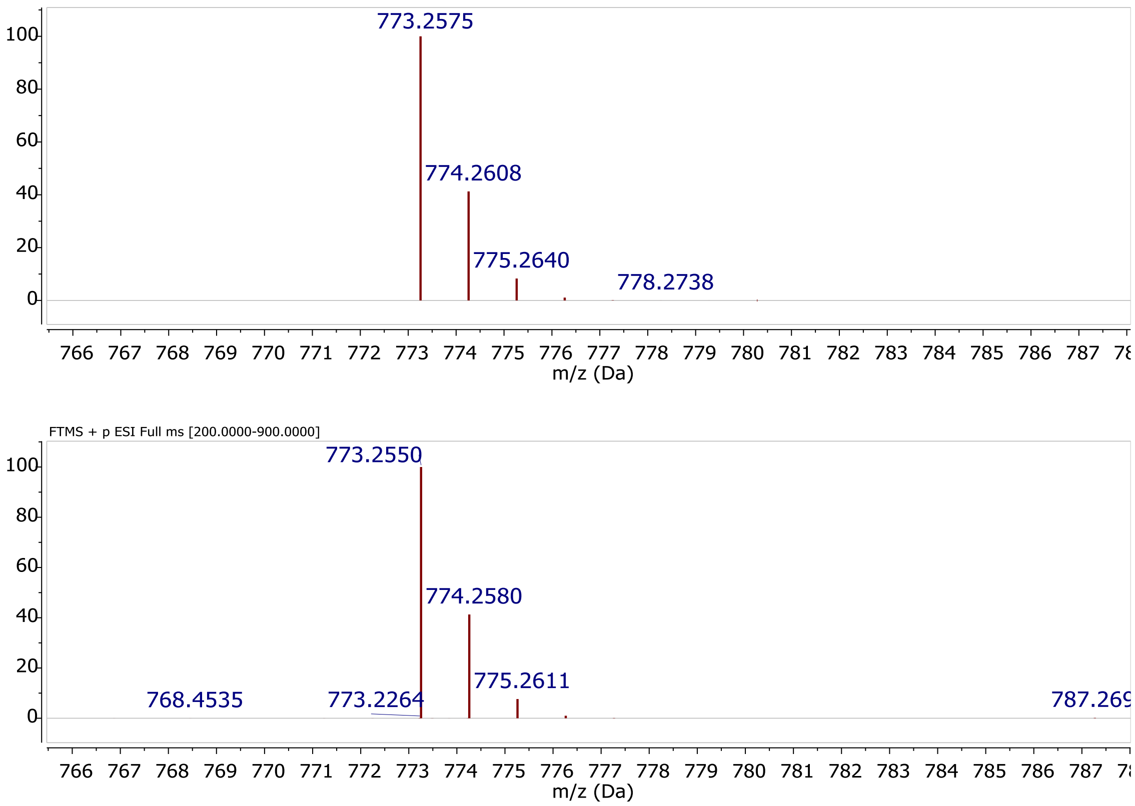

Figure S 61: MS (ESI+) **5a.** Top: simulated.

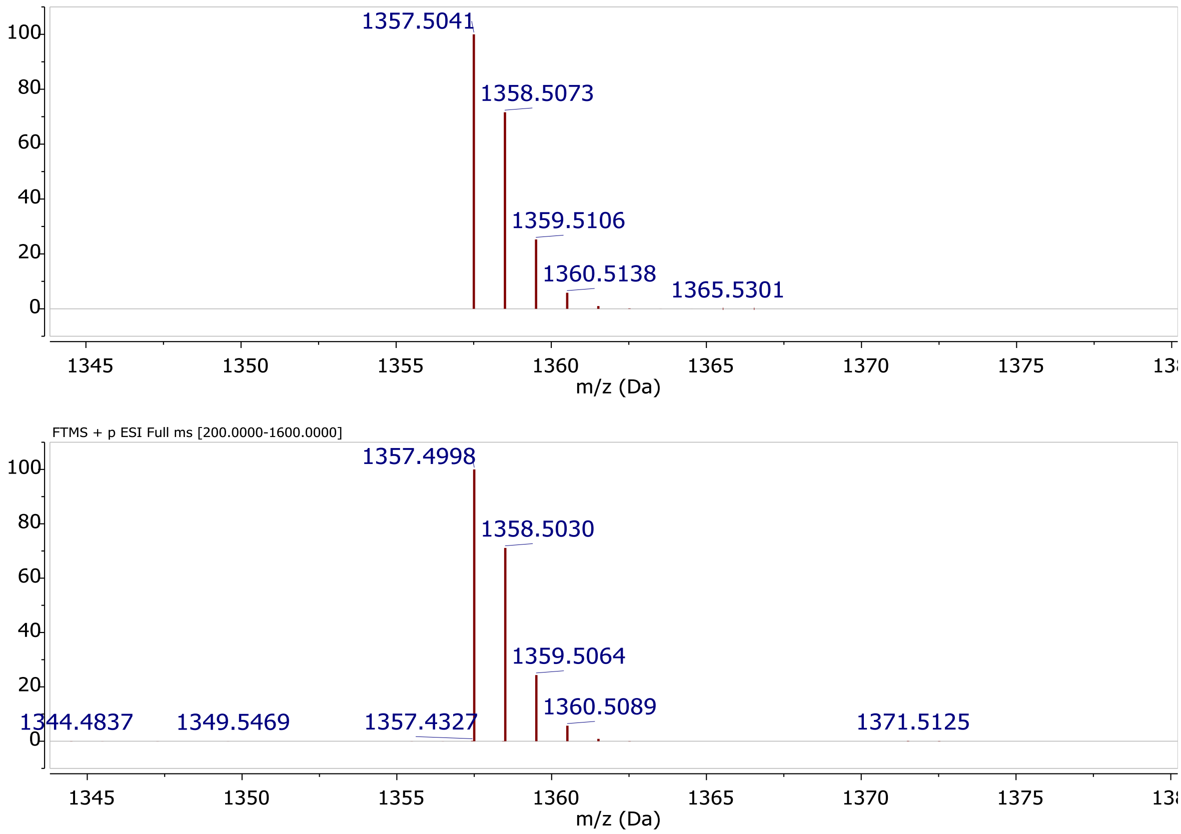

Figure S 62: MS (ESI+) **5b.** Top: simulated.

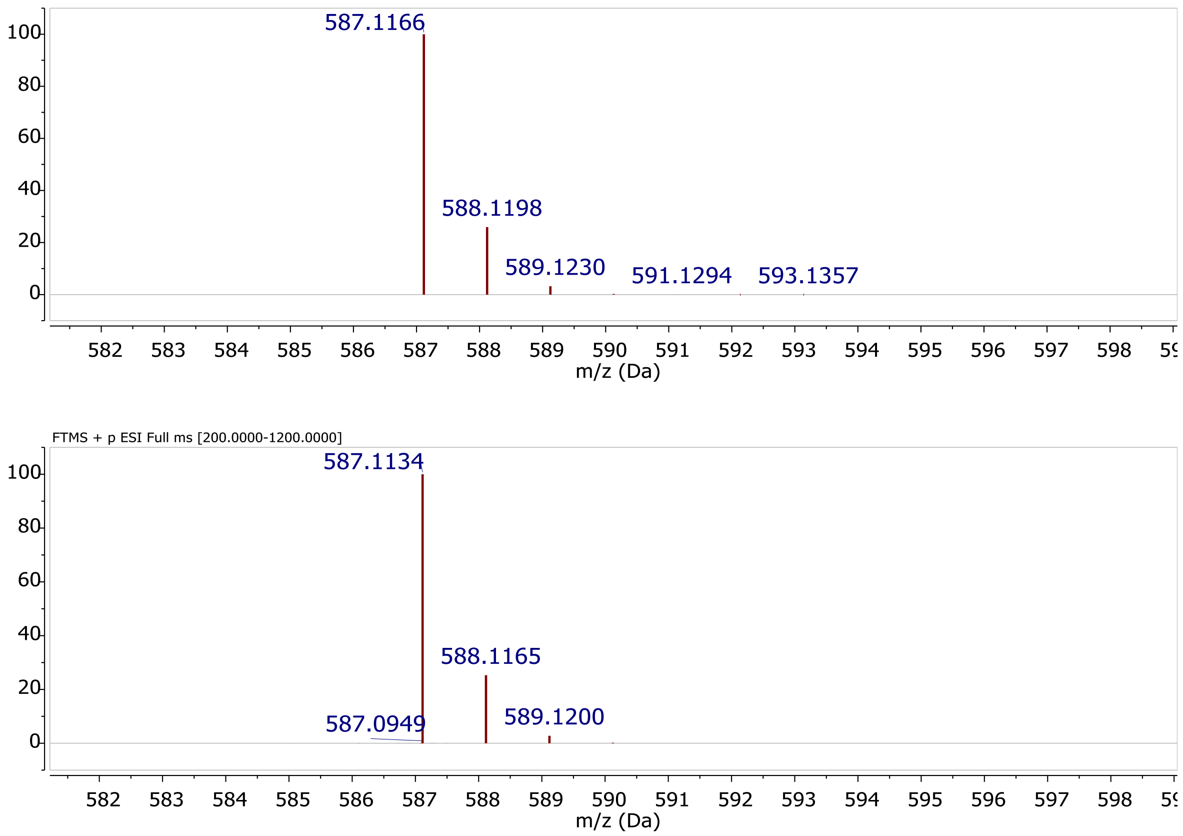

Figure S 63: MS (ESI+) ***4a.*** *Top: simulated.*

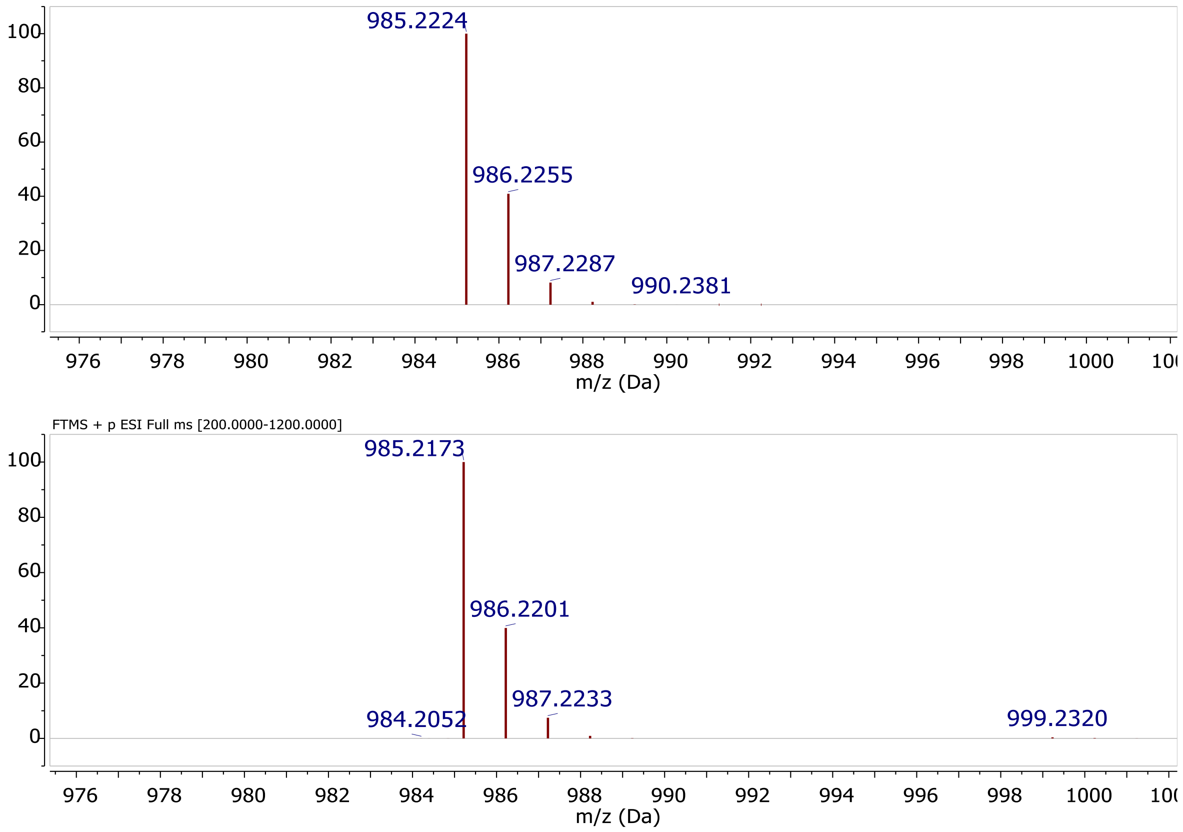

Figure S 64: MS (ESI+) ***4b.*** *Top: simulated.*

Cyclic voltammetry

Cyclic voltammograms were recorded in an argon filled glove box using a BioLogic SP-150 potentiostat and a three-electrode array (working electrode: glass like carbon, counter electrode: platinum wire, pseudo-reference electrode: silver wire). All experiments have been conducted in 0.15 M solutions of NBu_4PF_6 and 0.001 mM solutions of analyte in acetonitrile. Acetonitrile was kept over 3 Å molecular sieves for at least 2 days prior to use. The supporting electrolyte (NBu_4PF_6) was dried using fine vacuum (10^{-3} hPa) at 60 °C for 24 hours. All potentials were calibrated internally against the ferrocene/ferrocenium redox couple.

2a: $E_{1/2}$ = −1.57 V (half step potential of a reversible reductive event Co^{III}/Co^{II}). E_{pc} = −2.54 V (peak potential of an irreversible reductive event at 0.100 V s^{-1} Au^{I}/Au^{0})

2b: $E_{1/2}$ = −1.80 V (half step potential of a reversible reductive event Co^{III}/Co^{II}). E_{pc} = −2.60 V (peak potential of an irreversible reductive event at 0.100 V s^{-1} Au^{I}/Au^{0})

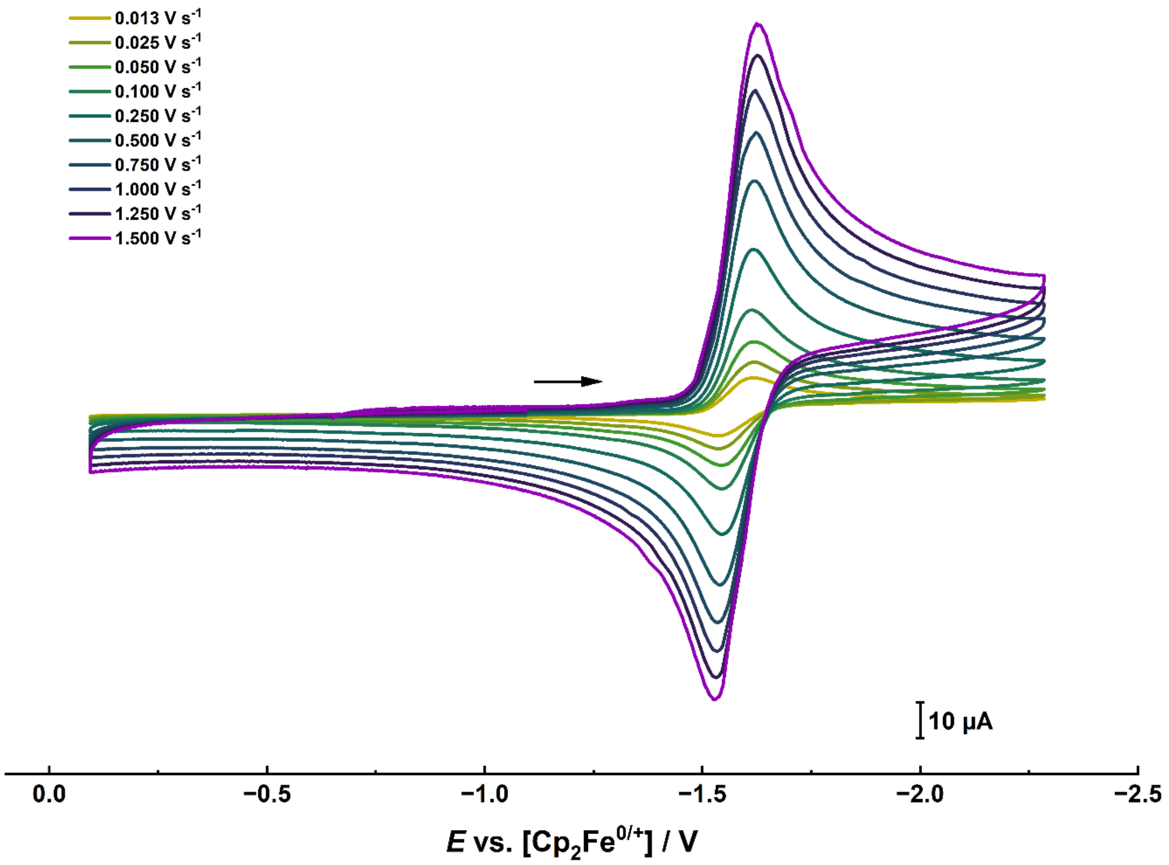

*Figure S 65: Cyclic voltammogram of **2a** at scan rates from 13 mV/s to 1500 mV/s in CH_3CN/0.15 M $NBu_4^+PF_6^-$.*

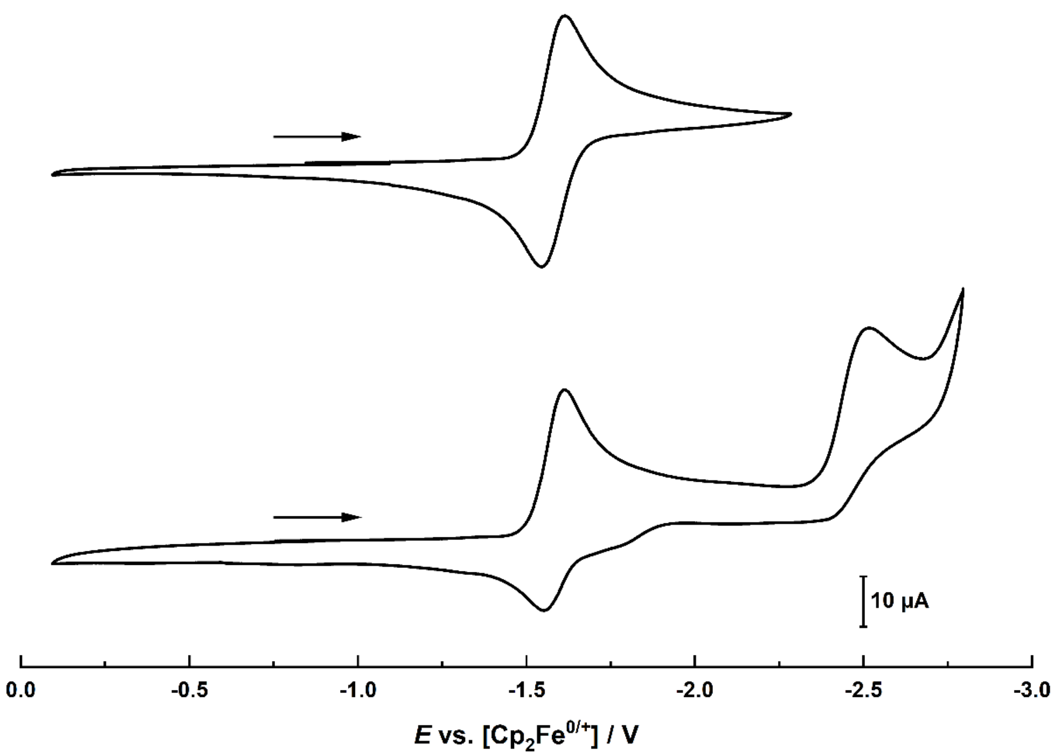

*Figure S 66: Full range cyclic voltammogram of **2a** at a scan rate of 100 mV/s in CH_3CN/0.15 M $NBu_4^+PF_6^-$.*

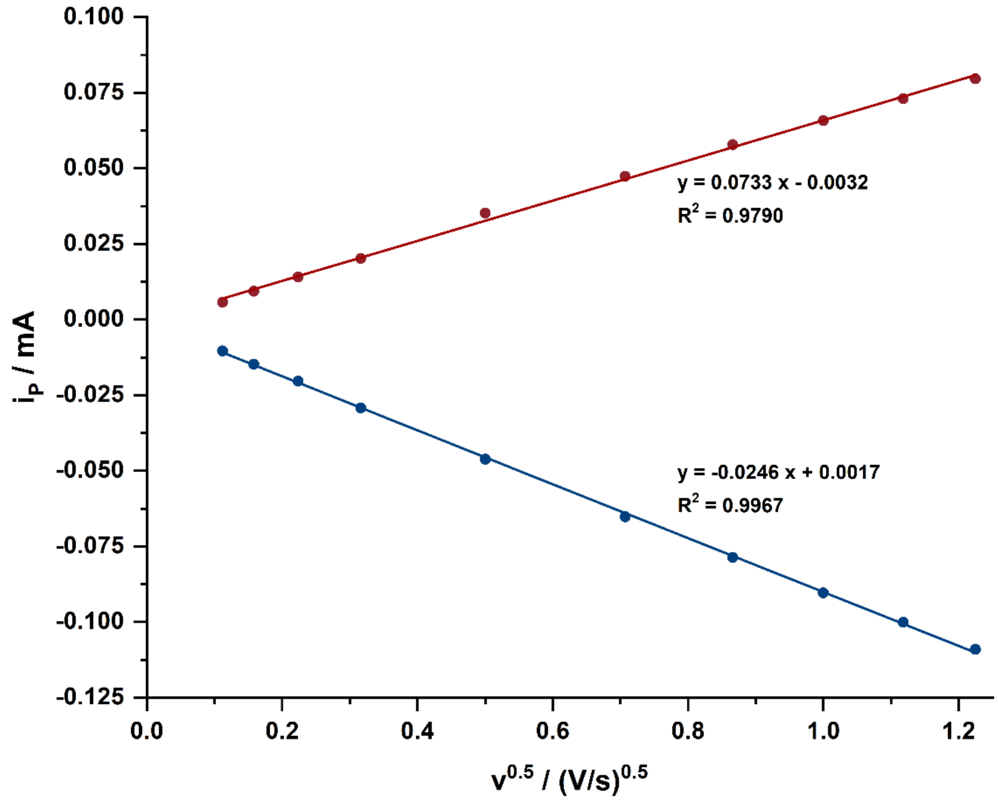

*Figure S 67: Reversibility plot for the first reduction of **2a**.*

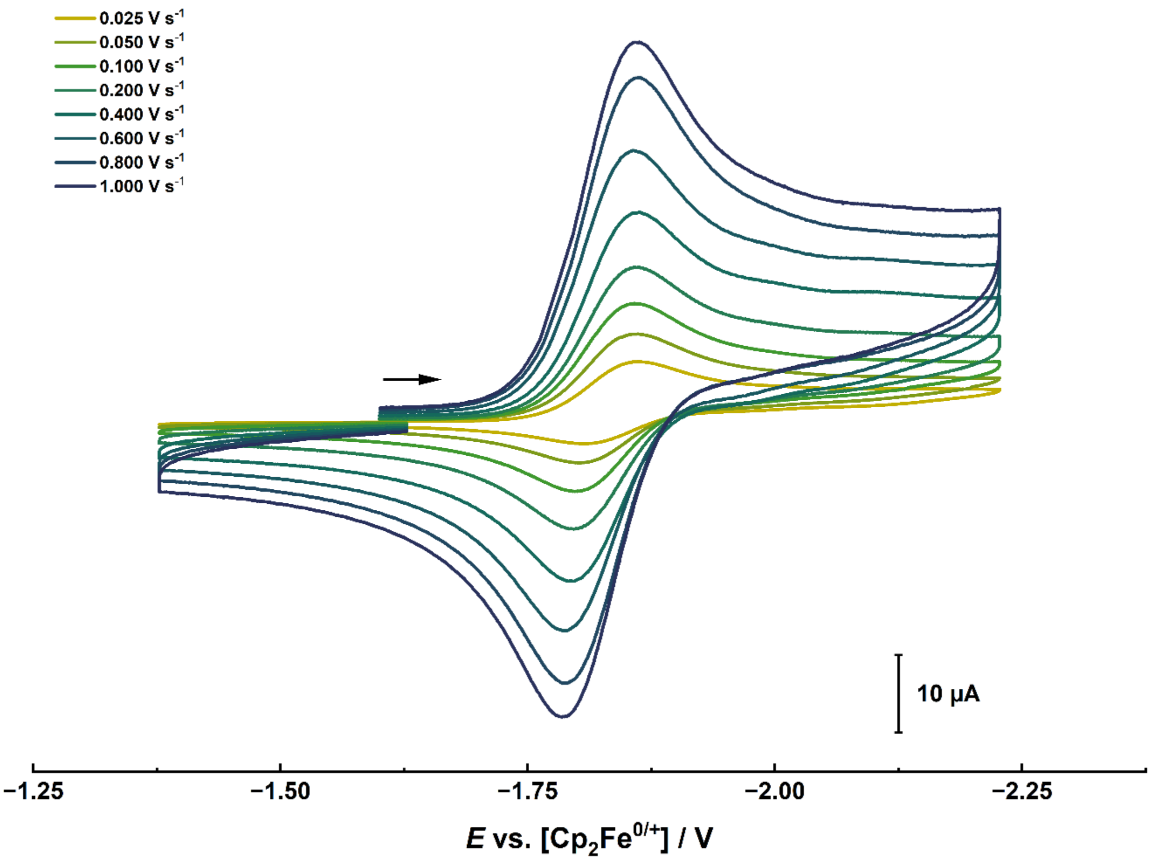

*Figure S 68: Cyclic voltammogram of **2b** at scan rates from 25 mV/s to 1000 mV/s in CH_3CN/0.15 M $NBu_4^+PF_6^-$.*

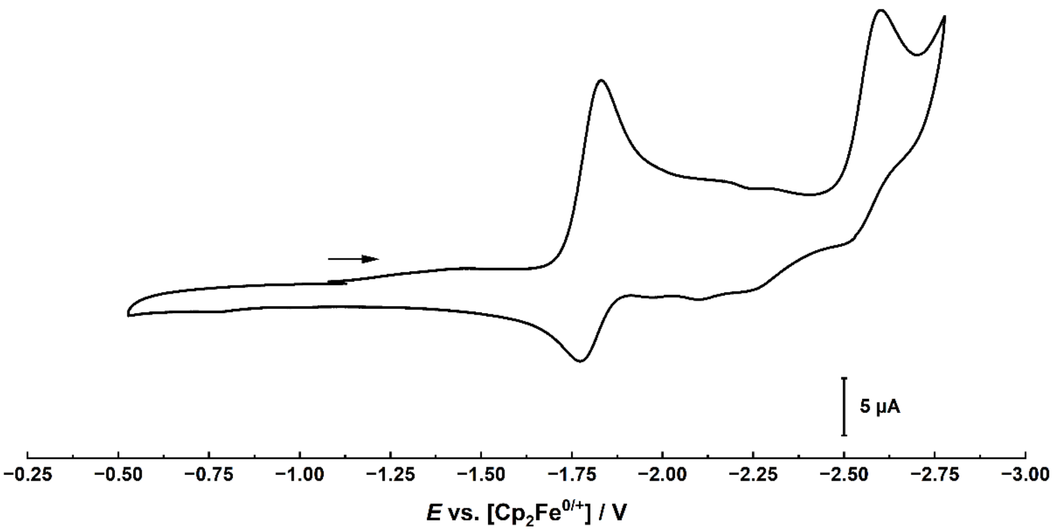

*Figure S 69: Full range cyclic voltammogram of **2b** at a scan rate of 100 mV/s in CH_3CN/0.15 M $NBu_4^+PF_6^-$.*

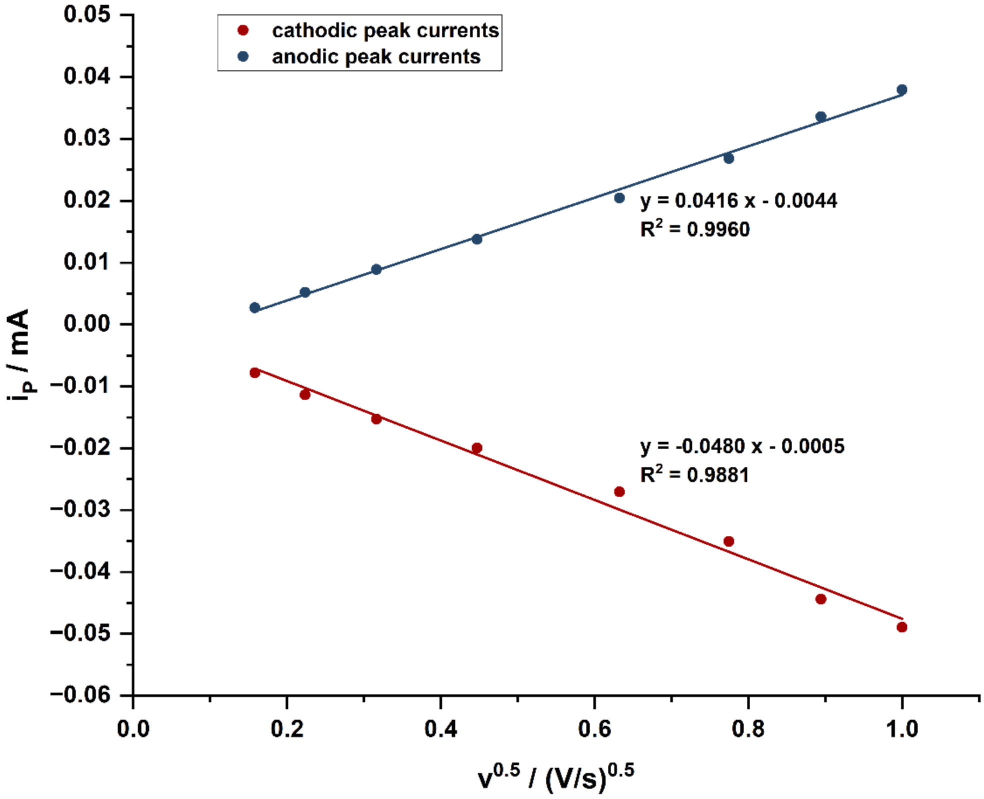

*Figure S 70: Reversibility plot for the first reduction of **2b**.*

Crystallographic data

*Table S1: Crystallographic details on complexes **2a,b** , **3a,b** and **4a,b**.*

	**2a**	**2b**	**3a***	**3b**	**4a**	**4b**	**5a**	**5b**
Chemical formula	$C_{28}H_{24}Co_1Au_1$ P_1F_6	$C_{46}H_{38}P_2Co_1Au_2$ P_1F_6	$C_{13}H_{18}P_1Co_1Au_1$ P_1F_6	$C_{16}H_{26}P_2Co_1Au_2$ P_1F_6	$C_{23}H_{27}N_2Co_1Au_1$ P_1F_6	$C_{36}H_{44}N_4Co_1Au_2$ P_1F_6	$C_{37}H_{45}N_2Co_1Au_1$ P_1F_6	$C_{64}H_{80}N_4Co_1Au_2$ P_1F_6 CH_3CN
M_r	792.31	1250.592	606.11	878.14	732.33	1130.58	918.61	1544.20
Crystal system	Orthorhombic	Monolinic	Triclinic	Triclinic	Monoclinic	Orthorhombic	Monoclinic	Monoclinic
Space group	$P2_12_12_1$	$P2_1$	*P*-1	*P*-1	$P2_1/n$	$P2_12_12_1$	$P2_1/c$	$P2_1/n$
a (Å)	11.0209(4)	10.2471(3)	7.3917(8)	7.5721(3)	10.3167(3)	10.5205(4)	15.3280(7)	18.8509(6)
b (Å)	12.9996(5)	10.8285(3)	10.0138(11)	10.2081(4)	18.8606(6)	16.7597(6)	13.7476(5)	18.2773(6)
c (Å)	19.0417(7)	19.6984(6)	12.8368(14)	16.7183(6)	12.9706(5)	21.5735(9)	19.3100(8)	19.9621(6)
□□(°)	90	90	97.978(4)	89.1070(10)	90	90	90	90
□ (°)	90	100.565(2)	99.284(4)	82.7650(10)	100.5610(10)	90	112.457(1)	107.1050(10)
□ (°)	90	90	110.929(3)	68.7100(10)	90	90	90	90
V (Å^3)	2728.05(18)	2148.69(11)	855.74(16)	1193.86(8)	2481.06(14)	3803.8(3)	3760.5(3)	6573.6(4)
Z	4	2	2	2	4	4	4	4
Densitiy (g cm^{-3})	1.929	1.933	2.352	2.443	1.961	1.974	1.623	1.560
F(000)	1525.8	1192.560	572	812	1416	2168	1821.557	3072
Radiation Type	MoKα	MoKα	MoKα	MoKα	MoKα	MoKα	MoKα	MoKα
μ (mm^{-1})	6.174	7.387	9.769	13.192	6.699	8.228	4.452	4.785
Crystal size	0.18x0.08x0.04	0.18x0.12x0.08	0.17x0.11x0.02	0.16x0.08x0.01	0.24x0.18x0.11	0.18x0.11x0.07	0.18x0.16x0.04	0.21x0.20x0.19
Meas. Refl.	62771	48768	2944	47229	71998	70909	85181	177936
Indep. Refl.	5394	9343	2944	5953	6419	8720	8653	16390
Obsvd. [$I > 2\sigma(I)$]	5180	9167	2893	5168	6063	8393	7178	14666
R_{int}	0.0512	0.0379	-	0.0387	0.0463	0.0379	0.0444	0.0563
R [$F^2 > 2\sigma(F^2)$]	0.0187	0.0142	0.0671	0.0268	0.0166	0.0231	0.0239	0.0191
wR(F^2)	0.0483	0.0330	0.1793	0.0691	0.0395	0.0551	0.0557	0.0460
S	1.0310	0.8168	1.078	1.045	1.050	1.112	1.0197	1.029
$\Delta\rho_{max}$	0.7091	0.4383	3.972	1.077	0.919	1.009	0.9112	0.681
$\Delta\rho_{min}$	-0.7400	-0.5611	-3.338	-1.230	-0.986	-0.495	-0.8604	-0.563
CCDC	2314980	2314979		2476574	2314982	2314981	2314983	2314984

*The crystals of **3a** were heavily twinned and weakly diffracting wherefore no full dataset could be collected. However, the model itself is valid and the connectivity can be unambiguously determined.

*Table S2: Selected bond metrics (lengths and angles) of the complexes **2a,b** , **3b** and **4a,b**.*

	**2a**	**2b**	**3b**	**4a**	**4b**	**5a**	**5b**
C1 – Au1	2.038(3)	2.070(2)	2.039(5)	2.0194(19)	2.016(6)	2.025(3)	2.023(2)
C10 – Au2	-	2.034(2)	2.040(5)	-	2.019(7)	-	2.021(2)
P1 – Au1	2.2941(8)	2.2964(5)	2.2768(14)	-	-	-	-
P2 – Au2	-	2.2831(6)	2.2816(12)	-	-	-	-
C11 – Au1	-	-	-	2.0294(18)	2.028(6)	2.025(3)	2.022(2)
C38/24 – Au2	-	-	-	-	2.041(6)	-	2.021(2)
C1 – C2	1.434(6)	1.439(4)	1.406(8)	1.437(3)	1.448(9)	1.440(5)	1.436(3)
C2 – C3	1.419(6)	1.429(3)	1.437(8)	1.425(3)	1.408(10)	1.420(5)	1.427(5)
C3 – C4	1.398(7)	1.410(4)	1.400(10)	1.412(4)	1.403(11)	1.410(7)	1.408(4)
C4 – C5	1.413(7)	1.428(4)	1.396(9)	1.422(3)	1.411(10)	1.431(5)	1.420(4)
C5 – C1	1.429(6)	1.434(3)	1.439(8)	1.440(3)	1.420(10)	1.420(5)	1.430(3)
C6 – C7	1.400(15)	1.424(4)	1.402(8)	1.413(4)	1.424(9)	1.405(10)	1.425(3)
C7 – C8	1.42(2)	1.420(3)	1.394(10)	1.431(4)	1.411(10)	1.422(11)	1.405(4)
C8 – C9	1.442(13)	1.421(4)	1.422(8)	1.402(4)	1.421(10)	1.416(11)	1.418(3)
C9 – C10	1.389(13)	1.443(3)	1.427(7)	1.391(4)	1.430(10)	1.418(9)	1.440(3)
C10 – C6	1.497(17)	1.446(3)	1.437(8)	1.388(4)	1.430(9)	1.427(9)	1.432(3)
Au1 – Au2	-	4.047(1)	7.509(1)	-	7.180(1)	-	6.328(1)
C1 – Au1 – P1/C11	177.92(13)	174.92(7)	177.36(15)	178.60(7)	175.9(3)	176.82(11	177.15(8)
C10 – Au2 – P2/C38/24	-	176.85(6)	177.10(16)	-	179.1(3)	-	179.03(8)
C1 – Au1 *vs.* C10 – Au2 Torsion angle	-	57.705(2)	147.679(2)	-	155.98(1)	-	108.92(8)

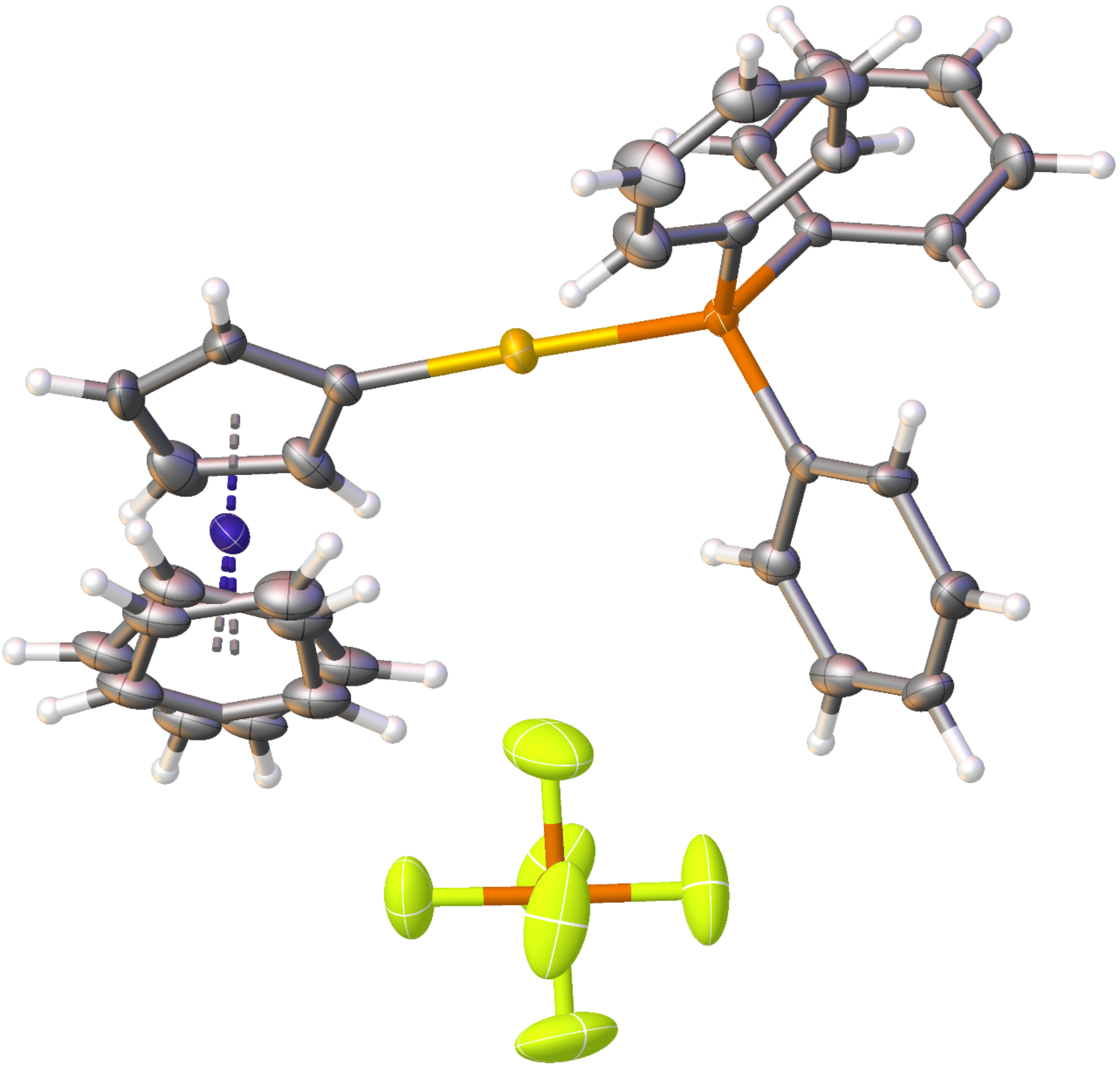

*Figure S 71: Full molecular view of **2a**. Ellipsoids are shown at a probability level of 50%.*

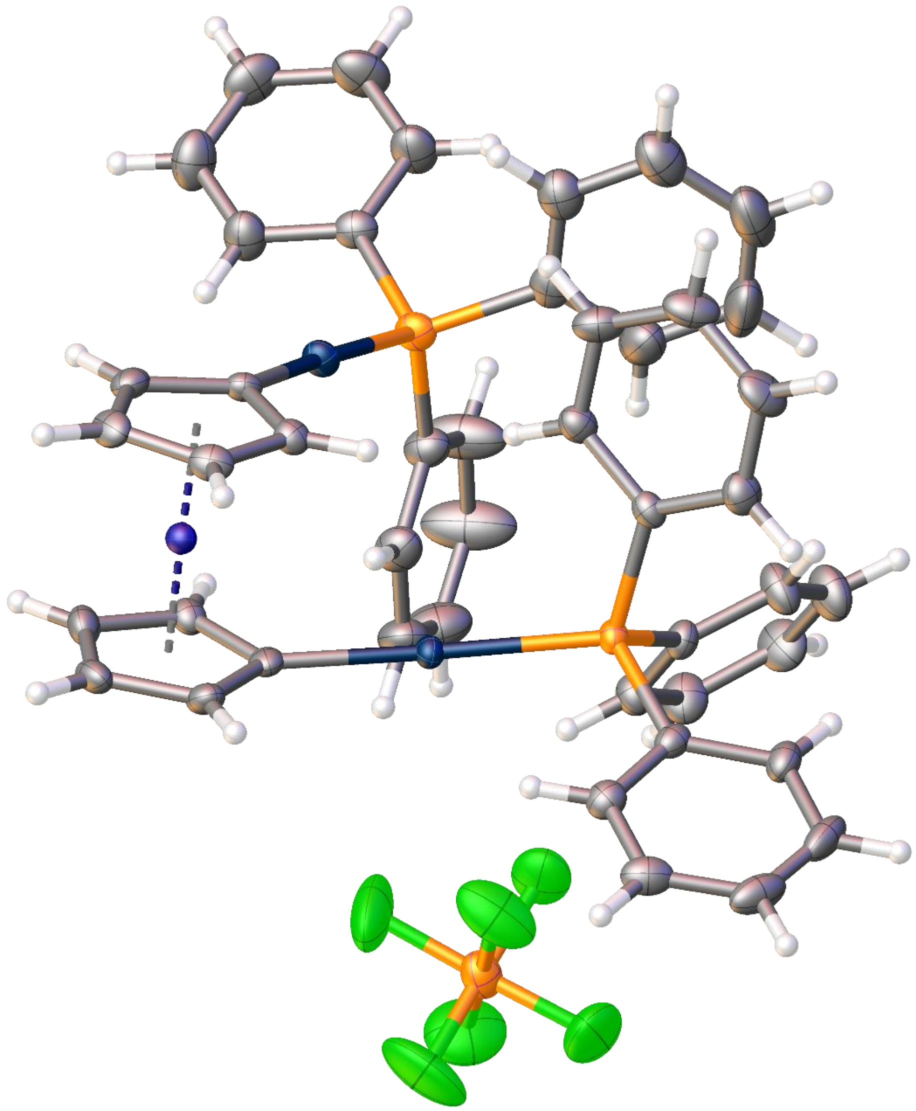

*Figure S 72: Full molecular view of **2b**. Ellipsoids are shown at a probability level of 50%.*

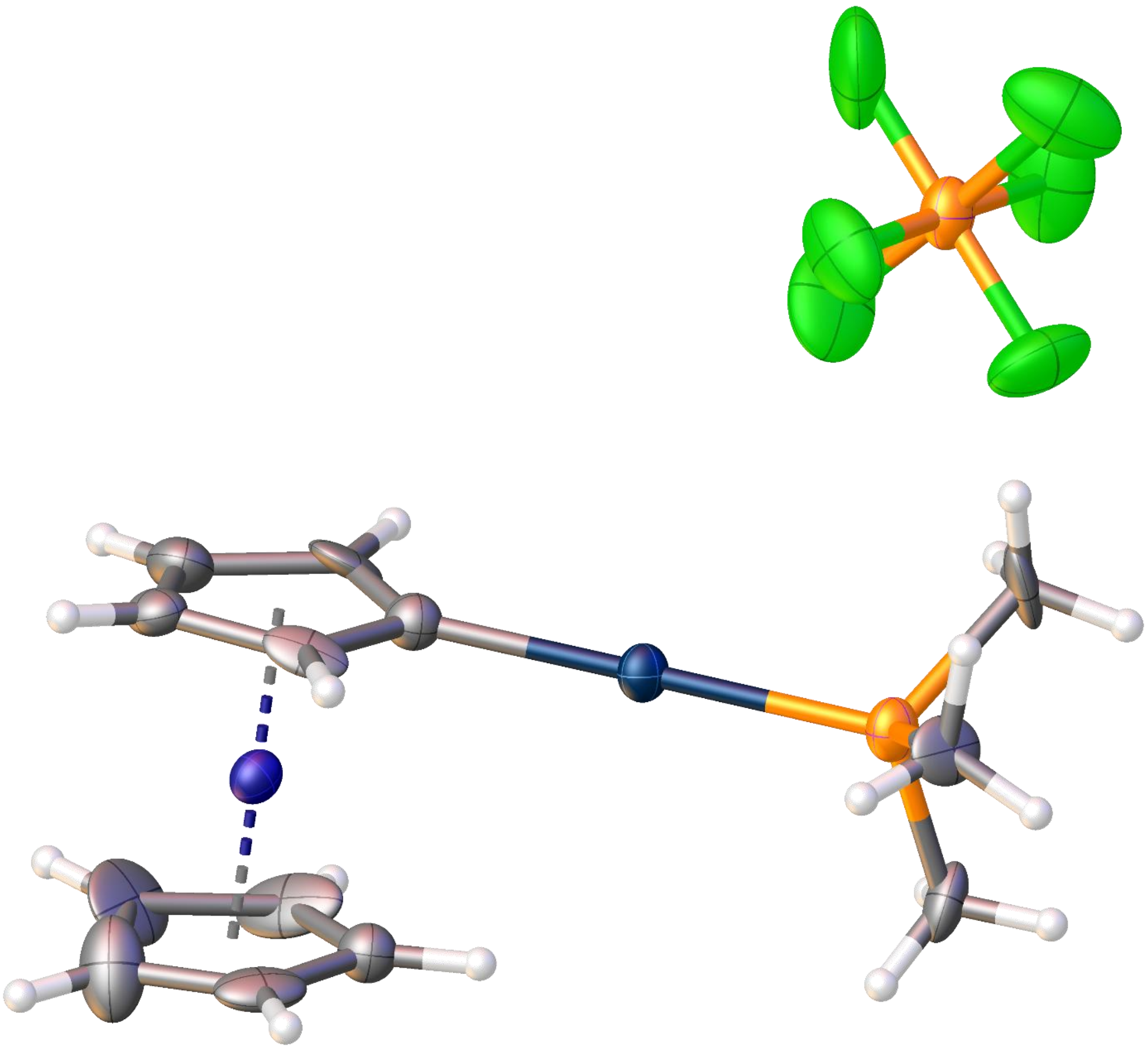

*Figure S 73: Full molecular view of **3a**. Ellipsoids are shown at a probability level of 50%.*

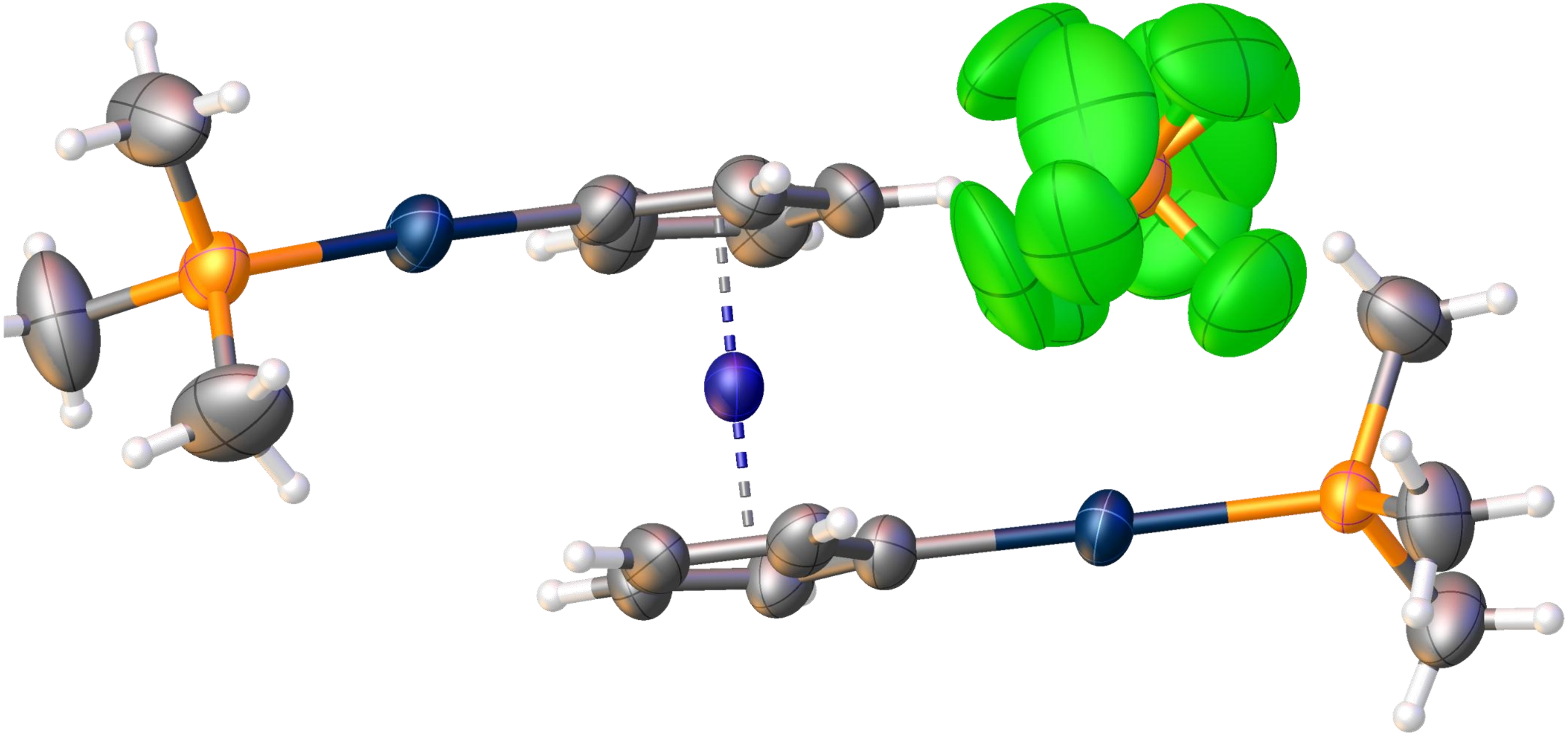

*Figure S 74: Full molecular view of **3b**. Ellipsoids are shown at a probability level of 50%.*

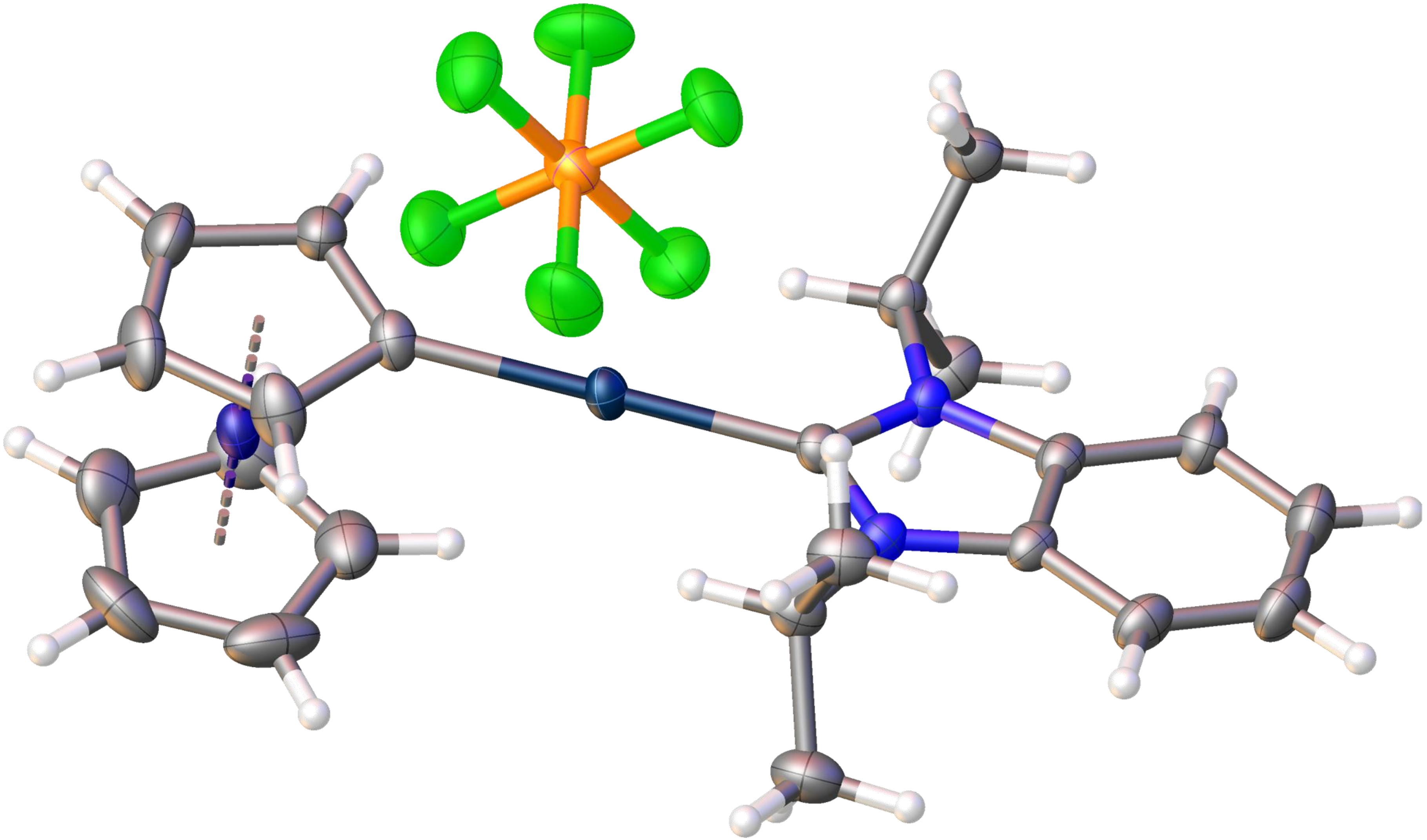

*Figure S 75: Full molecular view of **4a**. Ellipsoids are shown at a probability level of 50%.*

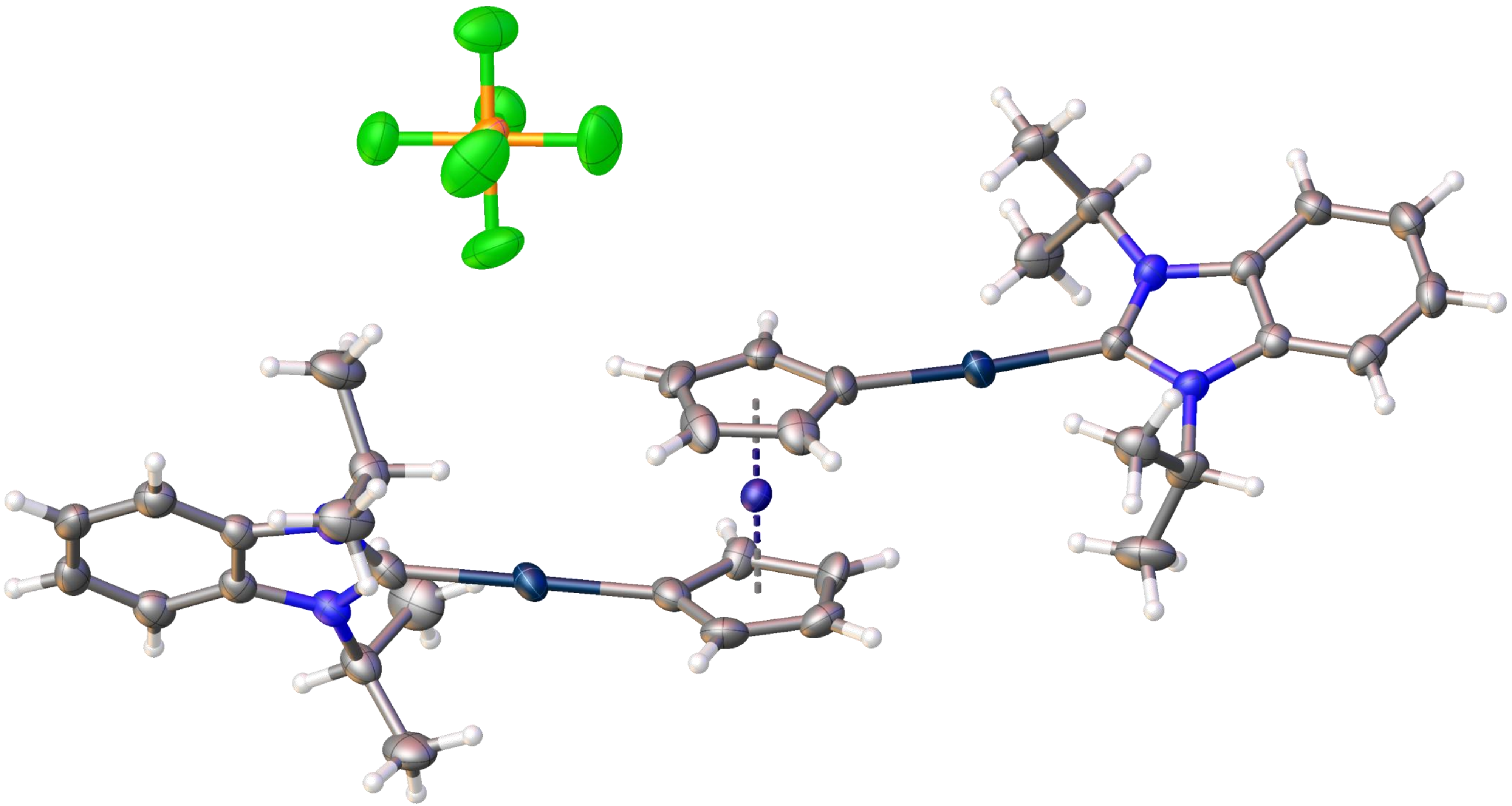

*Figure S 76: Full molecular view of **4b**. Ellipsoids are shown at a probability level of 50%.*

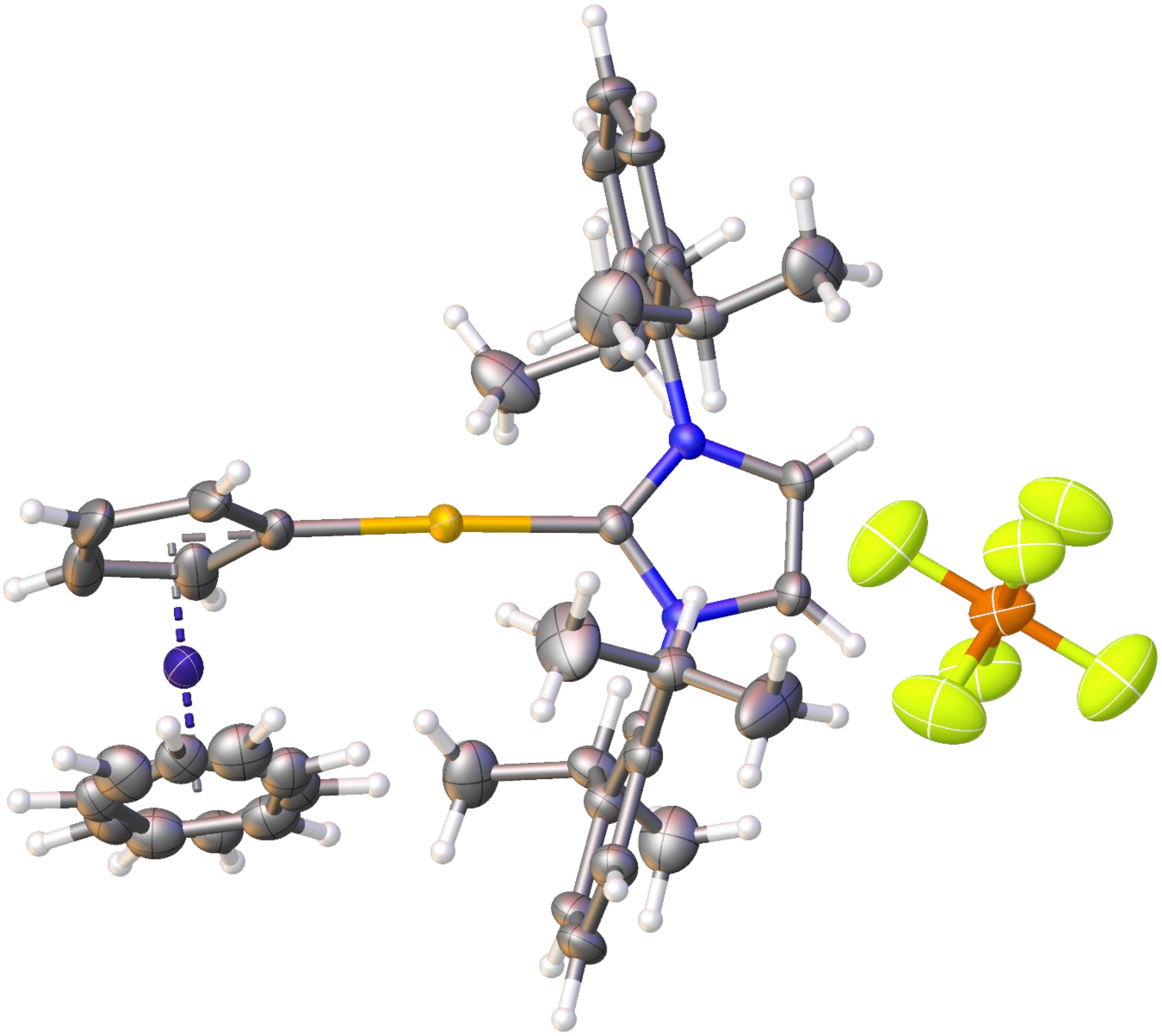

*Figure S 77: Full molecular view of **5a**. Ellipsoids are shown at a probability level of 50%. The positional disorder of the $[PF_6]^-$ anion is omitted for clarity.*

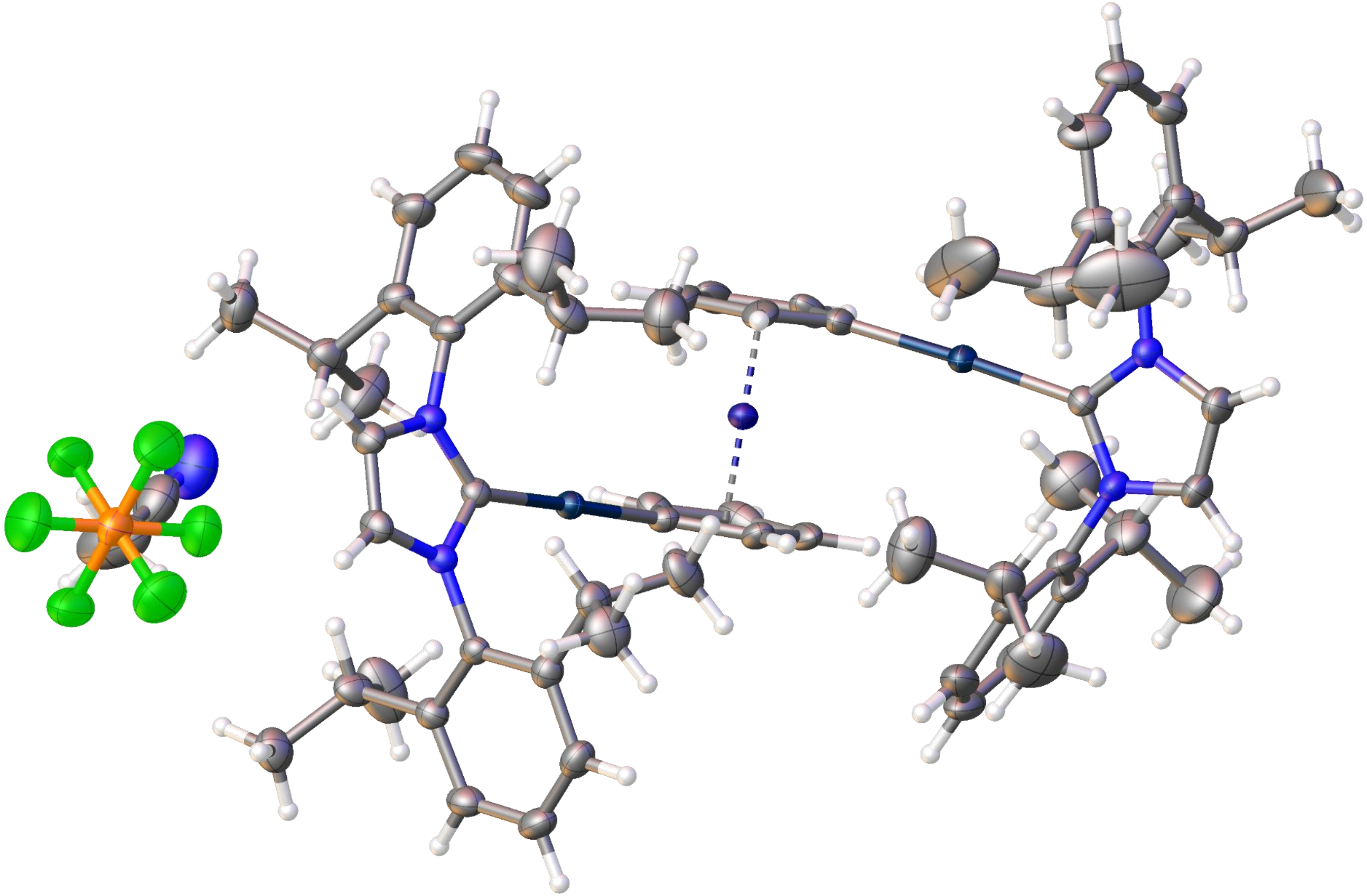

*Figure S 78: Full molecular view of **5b**. Ellipsoids are shown at a probability level of 50%.*

Computational Details

Computational Methodology
X-ray crystal structures of the structures **2a** and **2b** served as starting point for geometry optimizations in implicit solvent, utilizing the BP86 density[8,9] functional and the def2-SVP [10] and def2-TZVPP[10] basis sets in combination with empirical dispersion corrections with Becke-Johnson damping (D3BJ)[11] and an implicit solvent, modelled with a Conductor-Like Screening Model (COSMO) [12,13]as implemented in Turbomole. To account for polar solvent, a dielectric constant of 46.7 was used. Various spin states (singlet and triplet) were accounted for. To be able to compare the results of this study to a previous one, we also performed calculations with the hybrid PBE0 density functional.[14] As full optimizations of the dinuclear gold complex turned out to be computationally too demanding, only single points on the BP86 optimized geometries were calculated. A comparison of the two functionals for the mononuclear Au complex showed, however, that differences between the geometries were minimal. Relative energy differences of **2a** and **2b** as well as of their reduced forms are displayed in Table S 3. Unless noted otherwise, all calculations were performed with Turbomole 7.[15]
The isolated cobaltoceneylidene ligands [1a] and [1b]$^-$ as well as their reduced forms [1a]$^-$ and [1b]$^{2-}$ were also optimized separately using the same methodology as above, see Figure S 84 for structures. To assess the spin density of [1a]$^-$ and [1b]$^{2-}$ The spin density of the two reduced species were calculated with TPSSh/def2-TZVP[16,17] and D4[18] in implicit solvent, which was not available at the time of the initial study.. A molecular electrostatic potential map was calculated for [1b]$^{2-}$ on an isosurface of the density with an isovalue of 0.02 a.u according to the methodology that was used in our previous study (Figure S 85).[19] The Tolman Electronic Parameter (TEP)[20] value was computed according to a literature benchmark[21] with B3LYP/6-311++G**[22–25] for [1a] and [1b]$^-$ as well as for [1a]$^-$ and [1b]^2 using Gaussian16.
To compare with literature data, the spin state splittings as well as HOMO-LUMO energies of [1a] and [1b]$^-$ as well as imidazol-4-ylidene and triazol-5-ylidene (for structures see Figure S 86) were calculated with B3LYP/def2-TZVPP in the gas phase, using Turbomole (Table S 4). The Gaussian B3LYP with VWN3 was used as density functional for full consistency. Structures were visualized with PyMOL,[26] whereas spin-density plots were generated with VMD.[27]

*Table S 3; Relative energies of the singlet and triplet state of **2a** and **2b** as well as of their reduced forms.. Solvent effects were modelled implicitly with a COSMO model (see Methodology for details). Energies are given in kJ/mol.*

**Method**	**2a**		**2b**	
	Singlet	Triplet	Singlet	Triplet
BP86/def2-SVP/D3BJ	0.0	168.9	0.0	179.7
BP86/def2-TZVPP/D3BJ	0.0	170.0	0.0	181.1
PBE0/def2-SVP/D3BJ	0.0	122.4	0.0	122.8
PBE0/def2-TZVPP/BJ//BP86/def2-SVP/D3BJ	0.0	144.5	0.0	150.3
	**2a reduced**		**2b reduced**	
	Doublett	Quartet	Doublet	Quartet
BP86/def2-SVP/D3BJ	0.0	93.7	0.0	99.7
BP86/def2-TZVPP/D3BJ	0.0	92.6	0.0	101.3
PBE0/def2-TZVPP/BJ//BP86/def2-SVP/D3BJ	0.0	31.5	0.0	6.7

Table S 4: Spin-state splitting and HOMO and LUMO energy of [1a] and $[1b]^-$ as well as imidazole-4-ylidene and triazol-5-ylidene. To compare with literature data results were calculated with B3LYP/def2-TZVPP in gas phase in addition to PBE0/def2-TZVPP/D3BJ in DMSO. All structures were fully optimized with the respective method. Energies in kJ/mol kcal/mol values in parenthesis; HOMO and LUMO energy in eV and displayed for the singlet state.

	Method	Singlet	Triplet	HOMO	LUMO
[1a]	PBE0/def2-TZVPP/D3BJ/DMSO	0.0	88.5 (21.1)	-4.526	-4.229
	B3LYP/def2-TZVPP	0.0	44.0 (10.5)	-1.572	-2.639
$[1b]^-$	PBE0/def2-TZVPP/D3BJ/DMSO	0.0	107.6 (25.7)	-4.952	-0.758
	B3LYP/def2-TZVPP	0.0	72.2 (17.3)	-0.172	2.852
Imidazol-4-ylidene	PBE0/def2-TZVPP/D3BJ/DMSO	0.0	295.0 (70.5)	-6.069	0.063
	B3LYP/def2-TZVPP	0.0	250.6 (59.0)	-4.820	-0.150
Triazol-5-ylidene	PBE0/def2-TZVPP/D3BJ/DMSO	0.0	274.8 (65.7)	-6.348	-0.549
	B3LYP/def2-TZVPP	0.0	247.0 (59.0)	-5.230	-0.536

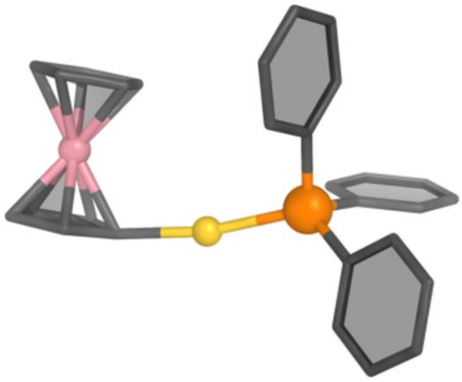

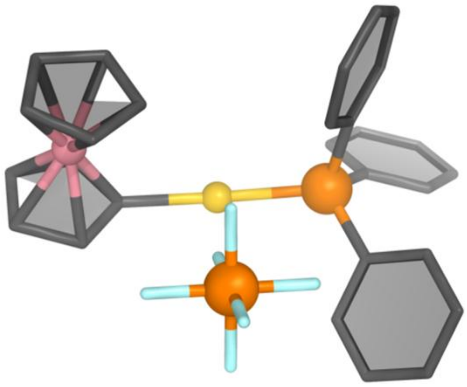

Figure S 79: Optimized structure of ***2a*** *in singlet state without (left) and with the PF_6^- counter ion (right).*

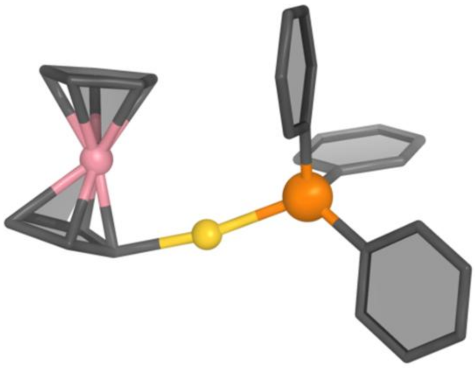

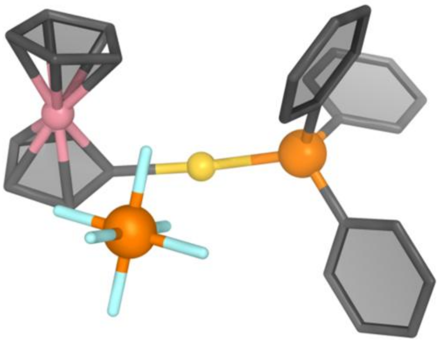

Figure S 80: Optimized structure of ***2a*** *in triplet state without (left) and with the PF_6^- counter ion (right).*

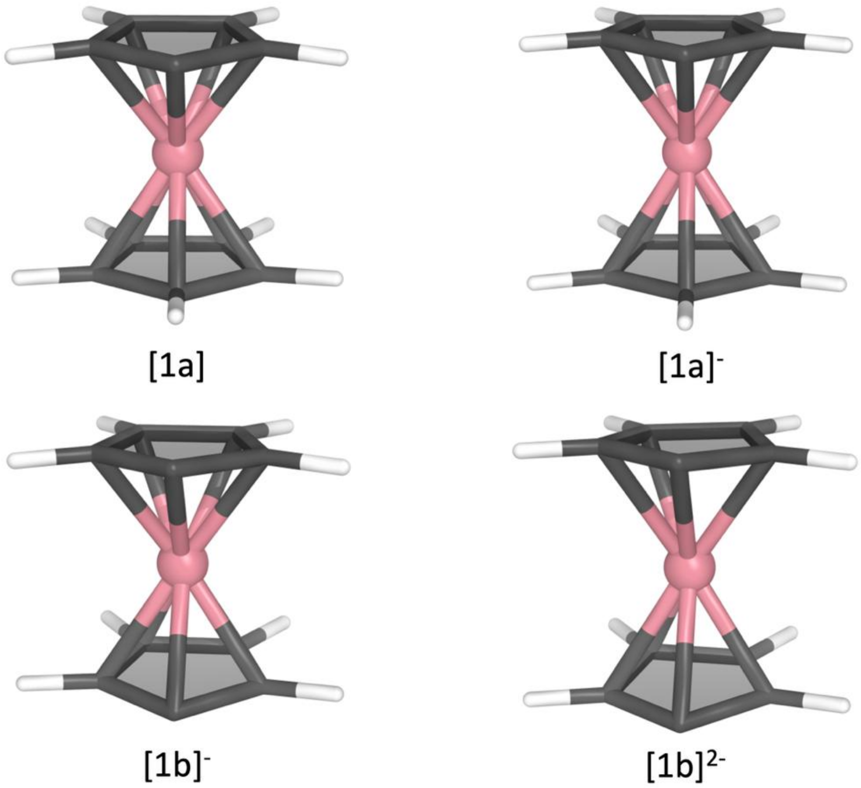

Figure S 81. Optimized structures of [1a], [1a]$^-$, [1b]$^-$, and [1b]$^{2-}$.For all species the most stable singlet or doublet state structure is depicted. Results were obtained with PBE0/def2-TZVPP/D3BJ in DMSO.

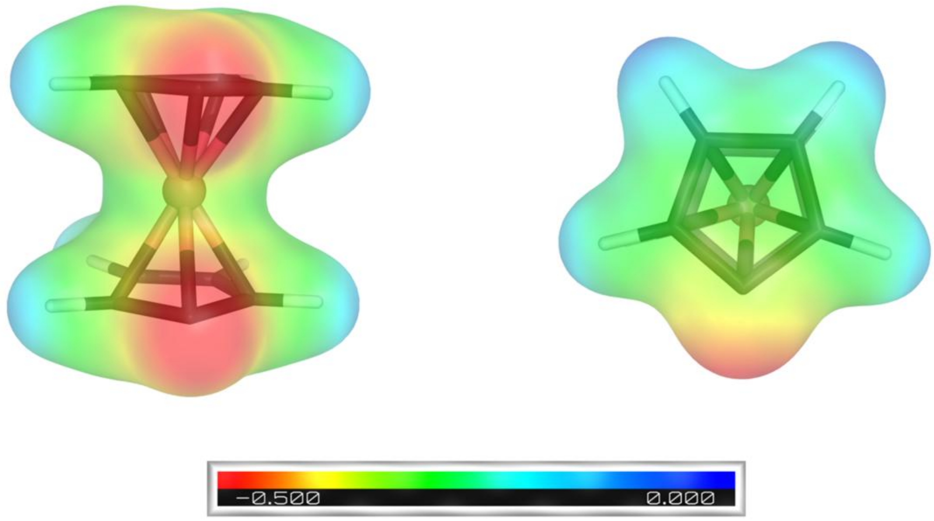

Figure S 82: Molecular electrostatic potential map of the reduced ligand [1b]$^{2-}$. Note that the scale only extends to the negative region.

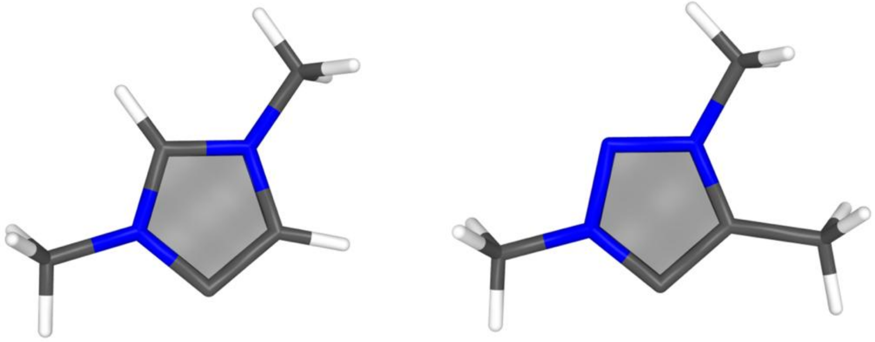

Figure S 83: Optimized structures of two representative N-heterocyclic carbenes, imidazole-4-ylidene and triazol-5-ylidene in their most stable singlet state. Structures were optimized with PBE0/def2-TZVPP/D3BJ in DMSO.

References

[1] J. K. Pagano, E. C. Sylvester, E. P. Warnick, W. G. Dougherty, N. A. Piro, W. S. Kassel, C. Nataro, *J. Organomet. Chem.* **2014**, *750*, 107.

[2] G. M. Sheldrick, *Acta Cryst A* **2015**, *71*, 3.

[3] O. V. Dolomanov, L. J. Bourhis, R. J. Gildea, J. A. K. Howard, H. Puschmann, *J Appl Crystallogr* **2009**, *42*, 339.

[4] G. M. Sheldrick, *Acta Cryst C* **2015**, *71*, 3.

[5] A. L. Spek, *Acta Cryst C* **2015**, *71*, 9.

[6] C. Schmidt, L. Albrecht, S. Balasupramaniam, R. Misgeld, B. Karge, M. Brönstrup, A. Prokop, K. Baumann, S. Reichl, I. Ott, *Metallomics* **2019**, *11*, 533.

[7] I. Wiegand, K. Hilpert, R. E. W. Hancock, *Nature protocols* **2008**, *3*, 163.

[8] A. D. Becke, *Phys. Rev. A* **1988**, *38*, 3098.

[9] J. P. Perdew, *Phys. Rev. B* **1986**, *33*, 8822.

[10] F. Weigend, R. Ahlrichs, *Phys. Chem. Chem. Phys.* **2005**, *7*, 3297.

[11] S. Grimme, S. Ehrlich, L. Goerigk, *J. Comput. Chem.* **2011**, *32*, 1456.

[12] A. Klamt, G. Schüürmann, *J. Chem. Soc., Perkin Trans. 2* **1993**, 799.

[13] A. Schäfer, A. Klamt, D. Sattel, J. C. W. Lohrenz, F. Eckert, *Phys. Chem. Chem. Phys.* **2000**, *2*, 2187.

[14] C. Adamo, V. Barone, *J. Chem. Phys.* **1999**, *110*, 6158.

[15] S. G. Balasubramani, G. P. Chen, S. Coriani, M. Diedenhofen, M. S. Frank, Y. J. Franzke, F. Furche, R. Grotjahn, M. E. Harding, C. Hättig et al., *J. Chem. Phys.* **2020**, *152*, 184107.

[16] V. N. Staroverov, G. E. Scuseria, J. Tao, J. P. Perdew, *J. Chem. Phys.* **2003**, *119*, 12129.

[17] J. Tao, J. P. Perdew, V. N. Staroverov, G. E. Scuseria, *Phys. Rev. Lett.* **2003**, *91*, 146401.

[18] E. Caldeweyher, S. Ehlert, A. Hansen, H. Neugebauer, S. Spicher, C. Bannwarth, S. Grimme, *J. Chem. Phys.* **2019**, *150*, 154122.

[19] S. Vanicek, M. Podewitz, C. Hassenrück, M. Pittracher, H. Kopacka, K. Wurst, T. Müller, K. R. Liedl, R. F. Winter, B. Bildstein, *Chem. Eur. J.* **2018**, *24*, 3165.

[20] D. J. Nelson, S. P. Nolan, *Chem. Soc. Rev.* **2013**, *42*, 6723.

[21] J. Mathew, C. H. Suresh, *Inorg. Chem.* **2010**, *49*, 4665.

[22] A. D. Becke, *J. Chem. Phys.* **1993**, *98*, 5648.

[23] T. Clark, J. Chandrasekhar, G. W. Spitznagel, P. V. R. Schleyer, *J. Comput. Chem.* **1983**, *4*, 294.

[24] R. Krishnan, J. S. Binkley, R. Seeger, J. A. Pople, *J. Chem. Phys.* **1980**, *72*, 650.

[25] K. Raghavachari, G. W. Trucks, *J. Chem. Phys.* **1989**, *91*, 1062.

[26] Schrödinger, L., & DeLano, W. (2020). PyMOL. Retrieved from http://www.pymol.org/pymol.

[27] W. Humphrey, A. Dalke, K. Schulten, *J. Mol. Graph.* **1996**, *14*, 33-8, 27-8.
